# Color-tunable hot-exciton organic glassy supramolecular scintillators enabled by host–guest co-melting

**DOI:** 10.1039/d6sc00159a

**Published:** 2026-03-05

**Authors:** Yuan-Ji Ye, Xiang-Long Wei, Xi Yang, Yu-Dong Chen, Ming-Cen Weng, Hong-Ming Chen, Mei-Jin Lin

**Affiliations:** a College of Chemical Engineering, Fuzhou University Fuzhou 350116 P.R. China meijin_lin@fzu.edu.cn; b College of Chemistry, Fuzhou University Fuzhou 350116 P.R. China; c College of Materials Science and Engineering, Fuzhou University Fuzhou 350116 P.R. China chm@fzu.edu.cn; d Institute of Biology and Chemistry, Fujian University of Technology Fuzhou 350118 P.R. China

## Abstract

Organic glassy scintillators are promising for radiation detection owing to their low cost and facile processability. However, their performance is often constrained by insufficient X-ray absorption and scintillation quenching during vitrification from a single crystal to glass. Herein, we present a co-melting strategy that integrates a heavy-atom-containing fluorescent host (TPABr) with hot-exciton emitters (DTPA2F, TPE4Br and BTHDMF) to construct color-tunable organic glassy supramolecular scintillators. Notably, the TPABr–DTPA2F glass shows pronounced enhancements over pristine DTPA2F glass, including a ∼51% increase in Young's modulus and a ∼41% boost in radioluminescence intensity. These improvements arise from enhanced X-ray absorption and efficient host–guest energy transfer, ensuring high exciton utilization efficiency in co-melted glass. Besides, supramolecular interactions further provide a rigid microenvironment that suppresses nonradiative decay and stabilizes molecular packing, thereby maintaining high scintillation efficiency. The co-melted glass features an ultrafast lifetime of 1.69 ns and a relative light yield of 33 763 photons per MeV and can be processed into a >12 cm^2^ transparent scintillator screen *via* comelt-quenching. The resulting screen achieves 30.0 lp mm^−1^ static X-ray imaging resolution and eliminates afterglow artifacts in dynamic imaging of vascular models and small biological specimens, demonstrating potential applications for advanced X-ray imaging.

## Abstract 2

A host–guest co-melting strategy enables organic glassy supramolecular scintillators (OGSSs) with efficient energy transfer and stable excitons, achieving tunable emission and afterglow-free dynamic X-ray imaging.

## Graphical abstract

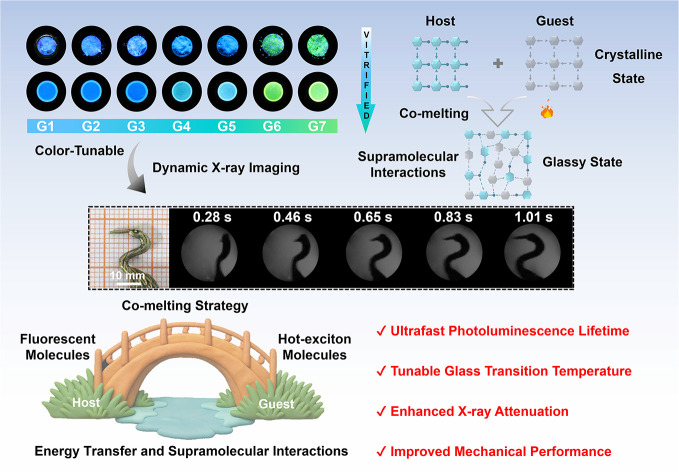

## Introduction

Scintillators are a class of functional materials capable of converting high-energy X-ray photons into visible light, and are widely used in medical diagnostics, industrial non-destructive testing, security screening and high-energy physics applications.^1^ Currently, most commercial scintillators are based on inorganic materials. Despite their excellent X-ray response performance, these materials suffer from high production costs, complex fabrication processes, and poor compatibility with flexible or customizable device platforms, which significantly limit their broader adoption in scalable and personalized application scenarios.^2^ In contrast, organic scintillators have garnered increasing attention as next-generation radiation detection materials due to their tunable molecular structures, tailorable photophysical properties, low-cost synthesis and facile processability.^3^ However, conventional organic scintillators still suffer from several intrinsic limitations, including low exciton utilization, poor radiation resistance, and structural instability under continuous X-ray irradiation. More importantly, traditional imaging modes such as thin-film or polymer-doped organic scintillators often suffer from severe optical reflection and scattering losses, which markedly reduce image resolution and signal-to-noise ratio, thereby hindering their application in high-definition X-ray imaging.^4–6^

Among the various strategies for constructing organic scintillators, vitrification into the glassy state has emerged as a key pathway toward practical implementation and to address the aforementioned challenges. Compared with crystalline or polymeric systems, organic glassy scintillators offer superior large-area processability and morphological control.^7,8^ Their intrinsically disordered molecular packing effectively suppresses grain-boundary scattering, thereby enhancing spatial resolution in X-ray imaging. Moreover, the amorphous nature of glassy materials imparts excellent flexibility and substrate compatibility, enabling integration into complex device architectures and opening avenues for high-resolution, dynamic X-ray imaging applications.^8^ In 2024, our group developed a series of cost-effective, large-area, and highly transparent fluorescent scintillators (PAM-M*X*, *X* = 2, 4, 6) by optimizing the alkyl side-chain length of 1,3-triaryl bismaleimide derivatives, achieving a spatial resolution of 27.0 lp mm^−1^ in static X-ray imaging.^9^ Subsequently, we further introduced host–guest doping^10^ and vitrification of hot-exciton molecules^8^ to enhance triplet exciton utilization in organic glassy scintillators, conceptually related to thermally activated delayed fluorescence (TADF) type triplet-to-singlet up-conversion reported for high-resolution organic X-ray scintillation and imaging.^11,12^ However, these studies have not yet addressed the substantial decrease in radioluminescence (RL) during the transition from crystalline to glassy phases, which arises from increased energetic disorder and the activation of nonradiative decay channels. Moreover, the lack of heavy atoms leads to inefficient X-ray absorption, while their incorporation often requires complex synthesis and causes severe fluorescence quenching.^13–15^ In addition, many organic molecules exhibit limited glass-forming ability and tend to recrystallize under thermal or storage conditions, compromising long-term stability and reusability. Therefore, developing a simple and general strategy to simultaneously enhance X-ray absorption efficiency, RL performance, and environmental stability of organic glassy scintillators is of great significance.

**Scheme 1:**
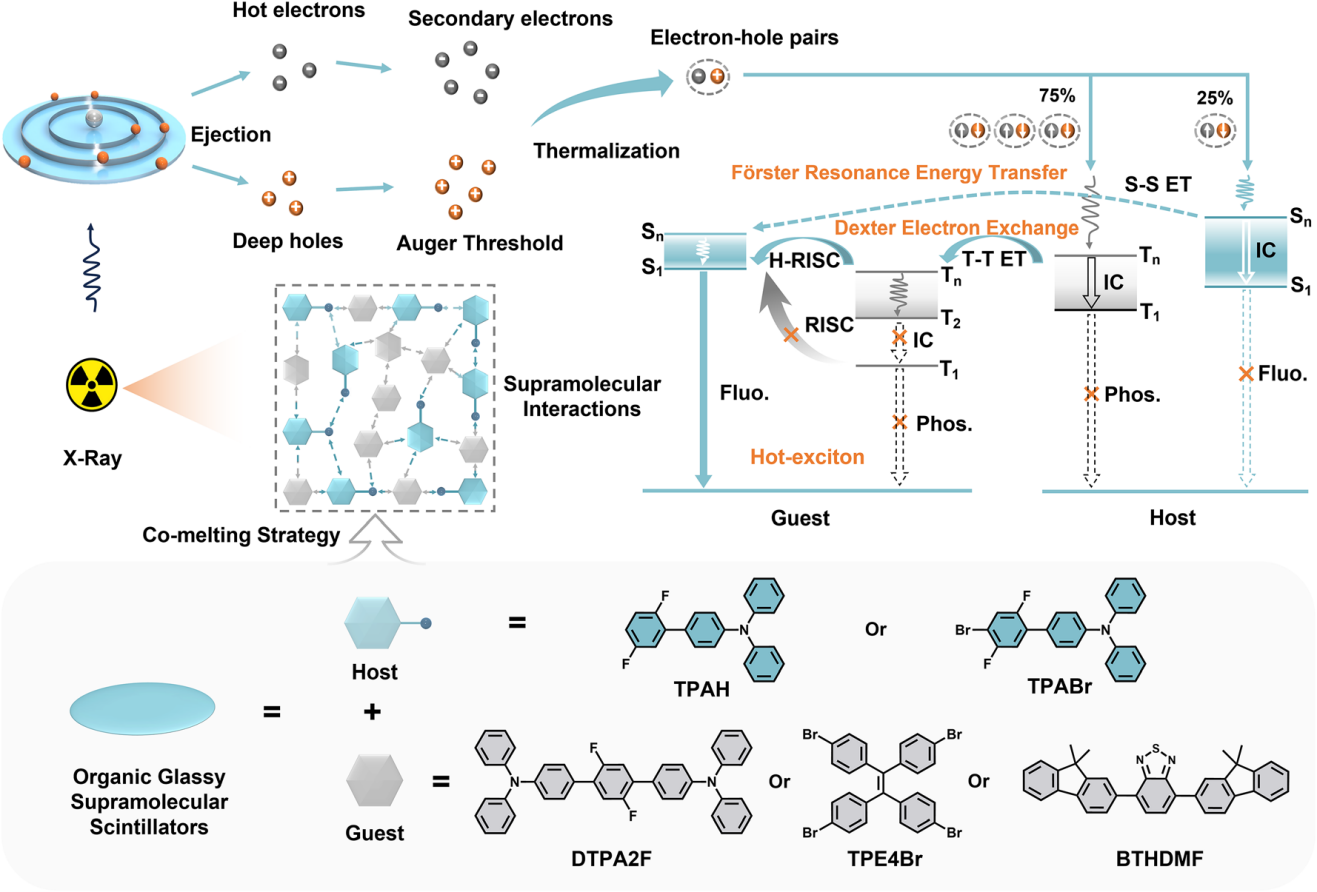
Schematic illustration of the host–guest co-melting strategy. The strategy is used for constructing organic glassy supramolecular scintillators (OGSSs), enabling synergistic exciton regulation *via* Förster resonance energy transfer (FRET), Dexter electron exchange, and hot-exciton harvesting under X-ray excitation.

Against this backdrop, the co-melting strategy has emerged as a promising approach for structural tuning and synergistic performance optimization in organic materials. Unlike conventional glass transition temperature (*T*_g_)-modulation methods that require complex backbone modifications such as long alkyl chains or rigid moieties,^4^ co-melting simply blends two or more small molecules in the molten state to form a thermodynamically stable amorphous phase, offering a more streamlined and cost-effective route.^6,16^ This strategy not only lowers *T*_g_ and improves glass-forming ability, but also provides a versatile platform for incorporating heavy atoms, enhancing X-ray attenuation, and reinforcing mechanical strength. For instance, Carlson *et al.* in 2017 demonstrated that co-melting distinct glass precursors could significantly improve anti-crystallization ability, with the resulting amorphous state maintained for over one month at 60 °C.^17^ Therefore, we envision that introducing heavy-halogen-containing host molecules into a eutectic system with highly radioluminescent guest emitters could enable efficient intermolecular energy transfer, thereby enhancing both X-ray absorption and RL performance. Moreover, in such host–guest architectures, the supramolecular interactions between the two components can construct a rigid intermolecular network, improving the mechanical strength and stability of the glass. These interactions also restrict molecular motion, effectively suppressing nonradiative decay pathways and enabling strong radiative responses in the glassy state (Scheme 1).

Building upon these insights, this study proposes a host–guest co-melting strategy design that integrates co-melting with energy-level alignment and supramolecular interaction engineering. Two asymmetric fluorescent host molecules, 2′,5′-difluoro-*N*,*N*-diphenyl-[1,1′-biphenyl]-4-amine (TPAH) and 4′-bromo-2′,5′-difluoro-*N*,*N*-diphenyl-[1,1′-biphenyl]-4-amine (TPABr), were designed with low *T*_g_ and energy levels compatible with hot-exciton emitters. By co-melting the three emissive guest molecules—2′,5′-difluoro-*N*4,*N*4,*N*4″,*N*4″-tetraphenyl-[1,1′:4′,1″-terphenyl]-4,4″-diamine (DTPA2F), 1,1,2,2-tetra(4-bromophenyl)ethene (TPE4Br), and 4,7-bis(9,9-dimethyl-9*H*-fluoren-2-yl)benzo[*c*][1,2,5]thiadiazole (BTHDMF)—separately with the fluorescent hosts, a series of color-tunable OGSSs (G1–G7) were successfully prepared. Among them, G3 (TPABr–DTPA2F, 1 : 1 mass ratio) exhibited the most balanced and outstanding performance. The heavy-atom-containing host TPABr provides strong X-ray absorption, while the hot-exciton guest DTPA2F ensures rapid and efficient emission. Benefiting from optimal host–guest energy alignment and extensive supramolecular interactions, G3 achieves efficient exciton harvesting and suppressed nonradiative decay. Consequently, it shows a 41% enhancement in RL intensity compared with the pure DTPA2F glass and approximately 10% higher emission than its crystalline counterpart, along with a high photoluminescence quantum yield (PLQY) of 75.6%, an ultrafast lifetime of 1.69 ns, and a relative light yield of 33 763 photons per MeV. The glass exhibits a low *T*_g_ of 50 °C, high optical transparency (>85%), and excellent screen formability (>12 cm^2^). Its Young's modulus and hardness are improved by approximately 51% and 116%, respectively, confirming superior mechanical robustness. Furthermore, G3 delivers high-resolution static X-ray imaging (30.0 lp mm^−1^, modulation transfer function (MTF) = 0.2) and stable dynamic imaging with negligible afterglow artifacts. Overall, this synergistic co-melting strategy, which combines molecular-level energy alignment with supramolecular network reinforcement, effectively addresses the long-standing issues of RL quenching, limited processability and mechanical fragility in OGSSs, providing a promising pathway for next-generation high-performance X-ray imaging materials.

## Results and discussion

### Synthesis, structural characterization and thermal properties

**Fig. 1:**
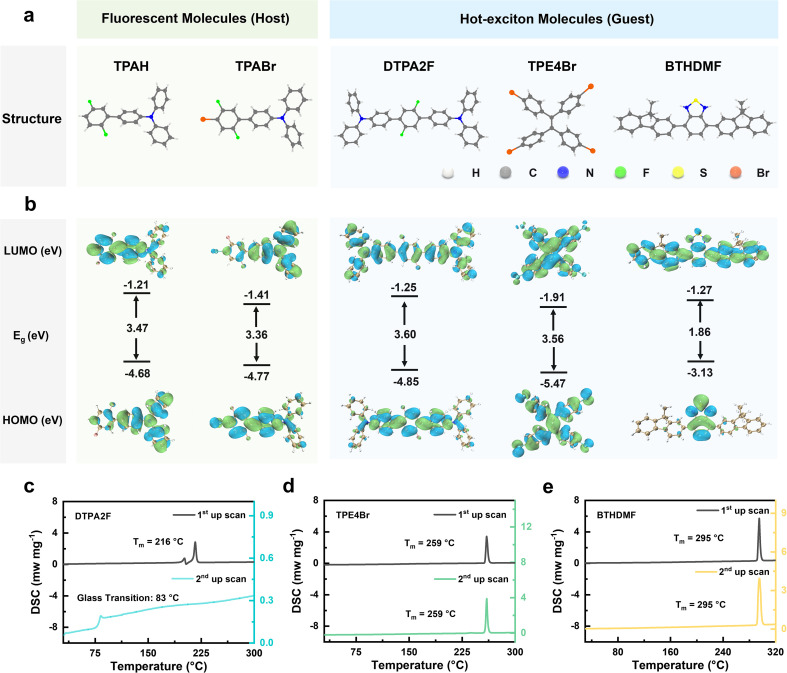
Molecular structures, electronic configurations, and thermal properties of host and guest molecules. (a and b) Chemical structures, HOMO/LUMO orbital distributions, and energy levels of fluorescent host molecules (TPAH and TPABr) and hot-exciton guest molecules (DTPA2F, TPE4Br, and BTHDMF). (c–e) DSC curves plot of DTPA2F, TPE4Br, and BTHDMF crystalline powders.

As shown in Fig. 1a and S1, S2, two rationally designed fluorescent host molecules, TPAH and TPABr, were synthesized *via* Pd-catalyzed Suzuki coupling reactions. These molecules feature asymmetric skeletons and twisted conformations, which are anticipated to inhibit molecular packing and promote glass-state formation. In addition, as shown in Fig. 1a and S3–S5, three hot-exciton molecules were prepared. DTPA2F and BTHDMF were synthesized *via* Pd-catalyzed Suzuki coupling reactions,^6,8,18,19^ while TPE4Br was synthesized from 4,4′-dibromobenzophenone *via* a facile and purified by column chromatography procedure modified from the literature.^20,21^ All compounds were purified by column chromatography. Their chemical structures were confirmed by ^1^H NMR spectroscopy (Fig. S6–S10), and their crystal structures were determined by single-crystal X-ray diffraction (Fig. S11 and S12). The corresponding crystallographic data have been deposited in the Cambridge Crystallographic Data Centre (CCDC) under deposition numbers 2428812, 2428818, 2357920, 2435305, and 2435306 (Table S1).^8,19,20^ Fig. S13 displays the solvent-dependent photoluminescence (PL) behavior of BTHDMF, along with a Lippert–Mataga plot showing a linear increase in Stokes shift with orientation polarizability (*f*). The dual-slope fitting observed in the plot reveals the coexistence of a local excited (LE) state and a charge transfer (CT) state, thereby confirming the hybridized local and charge-transfer (HLCT) excited-state character of BTHDMF.

Density functional theory (DFT) calculations were performed to elucidate the electronic structures of the host and guest molecules, as visualized in Fig. 1b.^22–24^ The fluorescent host TPABr exhibits a highest occupied molecular orbital (HOMO) and a lowest unoccupied molecular orbital (LUMO) energy of −4.77 and −1.41 eV, respectively, yielding a moderate bandgap of 3.36 eV. In comparison, its analogue TPAH shows a slightly wider gap of 3.47 eV. Notably, for the designed HLCT guest, the HOMO and LUMO show a partial spatial overlap, which is beneficial for balancing charge-transfer character with a high radiative rate. Among the guest molecules, DTPA2F presents a deeper HOMO (−4.85 eV) and a LUMO of −1.25 eV, resulting in a bandgap of 3.60 eV, indicative of strong electron-accepting ability and favorable energetic alignment with TPABr. TPE4Br displays a much deeper HOMO energy (−5.47 eV) and the lowest LUMO level (−1.91 eV) among all five molecules, corresponding to a bandgap of 3.56 eV. The pronounced orbital delocalization and significant dipole characteristics of TPE4Br suggest an enhanced ability to participate in through-space interactions, such as halogen bonding or dipole–dipole coupling. In contrast, BTHDMF shows the narrowest bandgap of 1.86 eV, with a HOMO level at −3.13 eV and a LUMO at −1.27 eV, implying higher exciton mobility and a different charge transport nature. These energetic and spatial orbital features collectively reveal distinct electronic characteristics between host and guest molecules, which not only influence exciton behavior but may also play a role in determining their thermal and phase-transition properties.

Thermal stability of the synthesized compounds was evaluated by thermogravimetric analysis (TGA), as shown in Fig. S14 and S15. The decomposition temperatures (*T*_d_) of TPAH, TPABr, DTPA2F, TPE4Br and BTHDMF were determined to be 249, 253, 417, 344 and 386 °C, respectively, indicating good thermal stability. In addition, differential scanning calorimetry (DSC) measurements revealed that the melting temperatures (*T*_m_) of TPAH, TPABr and DTPA2F were 70, 103 and 216 °C, respectively, as determined from the first heating scan (Fig. S16 and 1c). The corresponding *T*_g_ values obtained from the second heating scan were 16, 25 and 83 °C, respectively. Considering that a *T*_g_/*T*_m_ ratio of approximately 2/3 is generally regarded as the lower threshold for good glass-forming ability, the relatively high *T*_g_/*T*_m_ values of 0.84 (289.15 K/343.15 K), 0.79 (298.15 K/376.15 K) and 0.73 (356.15 K/489.15 K) for TPAH, TPABr and DTPA2F, respectively, suggest that these compounds are likely to form stable glassy materials.^25^ In contrast, as shown in Fig. 1d and e, the DSC curves of TPE4Br and BTHDMF exhibit only a melting transition (*T*_m_ = 259 °C and 295 °C, respectively), indicating poor glass-forming ability, likely due to their high molecular rigidity or symmetry.^26^

### Host–guest co-melting strategy for enabling glass formation

**Fig. 2:**
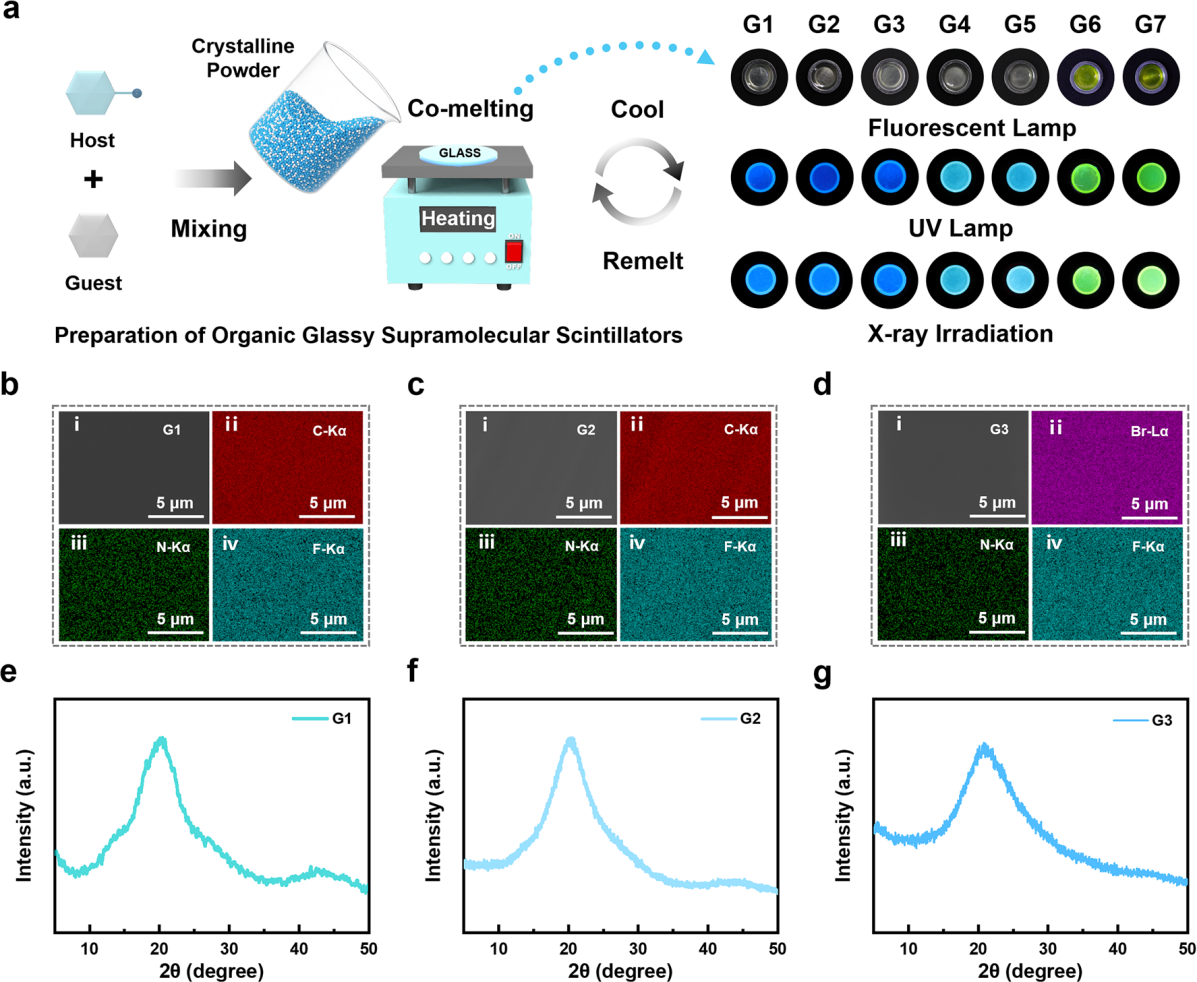
Co-melting preparation and characterization of OGSSs. (a) Schematic illustration of the co-melting strategy for preparing OGSSs *via* thermal vitrification of physically mixed crystalline host–guest powders. The right panel shows representative optical images of the resulting samples (G1–G7) under a fluorescent lamp, UV lamp, and X-ray irradiation. (b–d) SEM and EDS elemental mapping images of G1, G2, and G3. (e–g) XRD pattern of G1, G2, and G3.

To address the challenge of high glass transition temperatures and poor glass-forming ability in hot-exciton materials, a facile and generalizable co-melting strategy was developed by incorporating low-*T*_g_ fluorescent molecules (TPAH or TPABr) into hot-exciton matrices to promote vitrification. As shown in Fig. 2a, the fluorescent molecules (TPAH and TPABr) and hot-exciton emitters (DTPA2F, TPE4Br, and BTHDMF) co-melt to form homogeneous molten phases, which spontaneously vitrify into glassy supramolecular materials upon cooling. Based on this strategy, seven OGSSs, designated as G1–G7, were prepared *via* melt-quenching or co-melting at defined mass ratios (Table S2). TPAH, TPABr, and DTPA2F all exhibit clear glass transition behavior, enabling co-melting at arbitrary mass ratios. In this study, G2 and G3 were fabricated using a 1 : 1 mass ratio. In contrast, vitrification of TPE4Br- and BTHDMF-based systems required higher proportions of the glass-forming co-formers TPAH or TPABr, with threshold loadings of ≥50% and ≥67%, respectively. Accordingly, G4 and G5 were formulated at a 1 : 1 ratio, while G6 and G7 were prepared using a 2 : 1 ratio. As shown on the right side of Fig. 2a, the prepared glasses exhibit high transparency and emit bright luminescence under both ultraviolet (UV) lamp and X-ray irradiation. Notably, they can be remelted or annealed repeatedly without any degradation in morphology or optical performance.

To validate the structural uniformity and elemental integration resulting from this vitrification process, scanning electron microscopy (SEM) combined with energy-dispersive X-ray spectroscopy (EDS) elemental mapping of G1–G7 revealed a uniform distribution of key elements, including C, N, F, Br, and S, throughout the glass matrices (Fig. 2b–d and S17–S21). This confirms both the successful incorporation of heavy atoms and the compositional homogeneity achieved *via* the co-melting strategy. Their amorphous nature was confirmed by X-ray diffraction (XRD; Fig. 2e–g and S22), including in systems without an intrinsic glass transition, highlighting the effectiveness of the co-melting strategy in enabling vitrification across structurally diverse hot-exciton materials.

### Thermal, mechanical and supramolecular characteristics of OGSSs

**Fig. 3:**
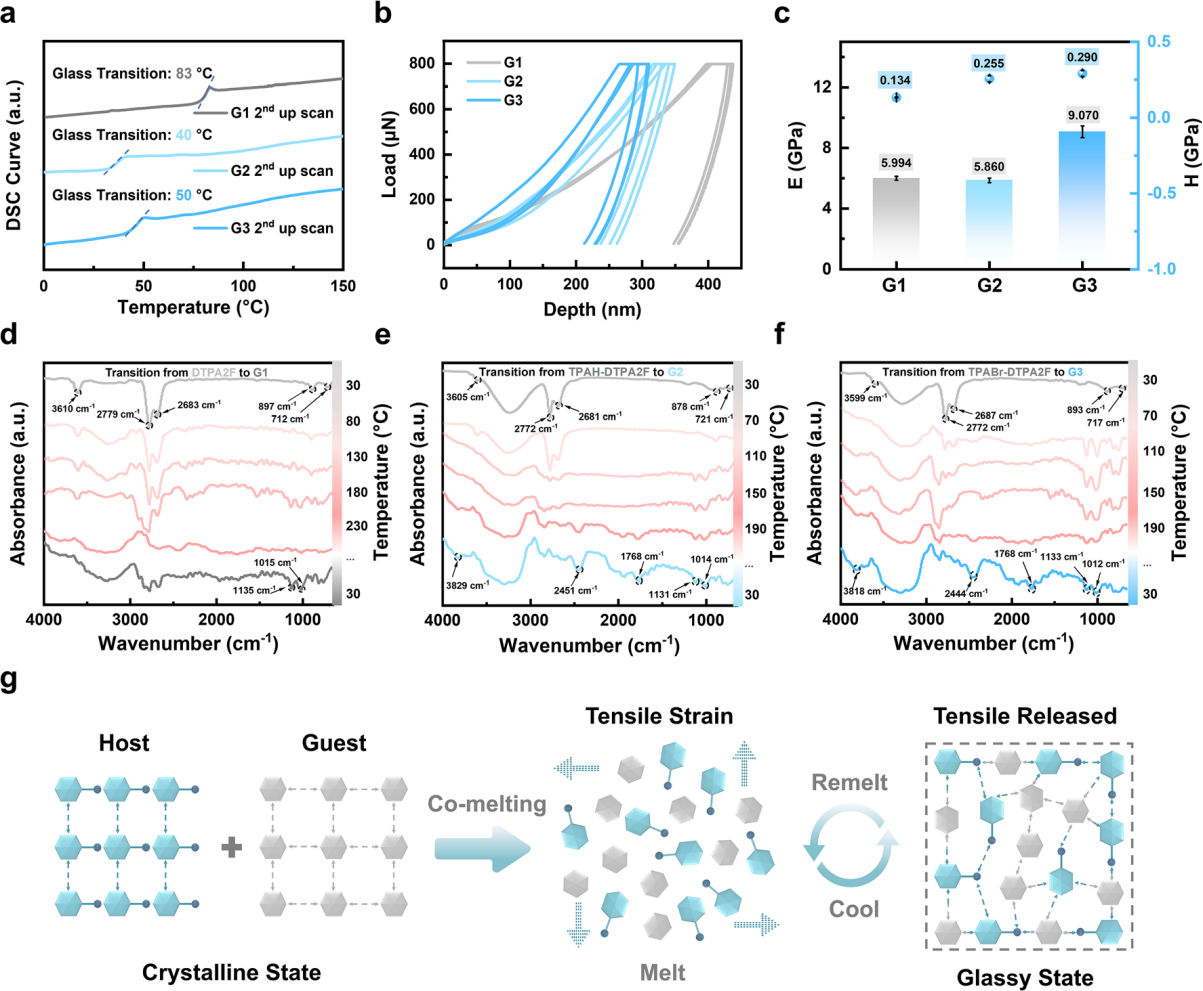
Thermal, mechanical and formation mechanism of OGSSs. (a) DSC curves of G1–G3 showing their respective glass transition temperatures. (b) Nanoindentation load–displacement curves of G1–G3. (c) The charts of Young's modulus (*E*) and hardness (*H*) values for G1–G3. (d) *In situ* FTIR spectra of G1 during heating (red) and cooling (cyan). (e and f) *In situ* FTIR spectra of G2 and G3 during heating (red) and cooling (blue). (g) Schematic illustration of the co-melting process and the formation mechanism of supramolecular glasses involving molecular rearrangement and tensile strain relaxation.

As shown in Fig. 3a and S23, all seven samples (G1–G7) exhibit clear glass transition behaviors in the second upward scan of DSC, confirming the successful vitrification of both pure and co-melting systems. Notably, compared to the pure hot-exciton glass G1 (*T*_g_ = 83 °C), the glass transition temperatures of G2 and G3 are significantly reduced to 40 and 50 °C, respectively. This reduction closely aligns with the intrinsic *T*_g_ values of the added fluorescent molecules TPAH and TPABr, suggesting their dominant role in modulating thermal behavior through co-melting. Furthermore, G4–G7, which are based on originally non-glass-forming hot-exciton matrices (TPE4Br and BTHDMF), also exhibit clear *T*_g_ values ranging from 14 to 28 °C. This observation demonstrates the success of the co-melting strategy in enabling vitrification, with the resulting *T*_g_ values largely governed by the fluorescent molecule component. These results collectively indicate that TPAH and TPABr serve as glass formers that facilitate the crystalline-to-amorphous phase transition. Complementary TGA (Fig. S24 and S25) shows that all glasses maintain good thermal stability with *T*_d_ ranging from 262 to 416 °C. In the first DSC up-scan (Fig. S26 and S27), *T*_m_ values were recorded from 33 to 216 °C. All samples satisfy the empirical glass-forming criterion of *T*_g_/*T*_m_ ≥ 2/3, further confirming their excellent glass-forming abilities.

As shown in Fig. 3b and S28, the load–displacement curves of G1–G7 obtained from multiple parallel nanoindentation tests are smooth and continuous without sudden breaks or turning points, indicating that these glasses possess homogeneous microstructures, which effectively suppress crack propagation and local collapse under concentrated stress.^6^ The corresponding elastic modulus (*E*) and hardness (*H*) values are summarized in Fig. 3c, S29 and Tables S3, S4. The pristine hot-exciton glass G1 exhibits an *E* of 5.994 ± 0.129 GPa and an *H* of 0.134 ± 0.003 GPa. In contrast, the co-melted glass G3 achieves the highest mechanical performance, with *E* = 9.070 ± 0.391 GPa and *H* = 0.290 ± 0.019 GPa, representing significant enhancements of ∼51% in stiffness and ∼116% in hardness compared with G1. Moreover, co-melting of TPAH or TPABr with TPE4Br or BTHDMF (G4–G7) yielded vitrified glasses with well-defined mechanical properties. As shown in Fig. S29 and Tables S3, S4, their *E* values range from 5.46 ± 0.07 GPa (G6) to 6.80 ± 0.33 GPa (G5), while the corresponding *H* values vary from 0.193 ± 0.001 GPa (G6) to 0.218 ± 0.017 GPa (G5). To benchmark their performance, the obtained *E* and *H* values were compared with those of other reported materials (Table S5).^6,27,28^ Notably, the co-melted organic glass G3 exhibits mechanical properties comparable to or even surpassing those of previously reported organic–inorganic hybrid glasses, while positioning itself between polymer glasses and inorganic glasses in terms of stiffness and hardness. These results further confirm the broad applicability of this strategy in tailoring the mechanical performance of OGSSs.

The mechanical enhancement of OGSSs can be attributed to denser molecular packing and stronger intermolecular interactions induced by the incorporation of rigid fluorescent co-formers, which simultaneously promote vitrification and reinforce the glassy network. In the case of TPABr, the presence of bromine may further stabilize the supramolecular structure through potential halogen bonding or π⋯Br interactions.^29,30^ These effects are supported by *in situ* Fourier-transform infrared (FTIR) spectroscopy, which reveals progressive spectral shifts from crystalline powders of DTPA2F, TPAH–DTPA2F, and TPABr–DTPA2F to their corresponding glassy forms (G1–G3). As shown in Fig. 3d–f, a series of characteristic vibrational changes emerged during the melting and cooling processes, providing molecular-level insights into the supramolecular reorganization induced by co-melting. At room temperature (∼30 °C), all samples (DTPA2F, G2, and G3) exhibited common vibrational bands at approximately 3610, 2779, 2683, 897, and 712 cm^−1^. These bands correspond to loosely associated N–H stretching (∼3610 cm^−1^), weak aromatic C–H stretching (2779 and 2683 cm^−1^), and ring out-of-plane deformations (897 and 712 cm^−1^), indicating that the initial state features relatively relaxed molecular packing and limited intermolecular coupling. Upon heating, new bands emerged near 1135 and 1015 cm^−1^, which are assigned to vibrational modes of C–N or C–Br bonds. These modes are typically IR-inactive or very weak in the crystalline state but become active due to changes in local dipole moments. Their appearance suggests enhanced vibrational polarizability, which is consistent with the formation of short-range dipole–dipole interactions involving nitrogen- and bromine-containing moieties during vitrification. Compared to G1, both G2 and G3 exhibited a redshift of the ∼712 cm^−1^ band, implying perturbed π–π stacking and increased asymmetry in aromatic ring environments, consistent with irreversible molecular reorganization upon co-melting.

Notably, co-melted systems G2 and G3 displayed a broad and intensified absorption band around 3200–3300 cm^−1^ that widened with increasing temperature. This feature is attributed to stronger N–H⋯Br or N–H⋯N hydrogen bonding, supported by the coexistence of nitrogen-rich donor sites and bromide acceptors in TPAH or TPABr. In G3, the appearance of a shoulder near 2451 cm^−1^ provides further evidence of polarized interactions, possibly involving N–H⋯Br bonding, which can shift vibrational frequencies into atypical regions. The peak at 1768 cm^−1^, which is absent in the initial state, is assigned to polarized C=N or C=C stretching modes influenced by charge redistribution and increased electronic coupling, further supporting the formation of an electronically heterogeneous glassy matrix.^31,32^ These observations collectively demonstrate that the co-melting process induces dipole reorientation, intermolecular polarization, and irreversible molecular reorganization. The activation of new vibrational modes and the emergence of redshifted or broadened peaks reflect the formation of short-range dipole–dipole interactions and halogen bonding between TPAH or TPABr and DTPA2F. These supramolecular interactions stabilize the amorphous network and likely underpin the enhanced thermal processability and mechanical robustness observed in G2 and G3, consistent with the stabilizing role of halogen bonding and related noncovalent interactions in supramolecular assemblies.^33^

X-ray photoelectron spectroscopy (XPS) analysis of the Br 3d region was conducted to further support this, as shown in Fig. S30. In crystalline TPABr, the Br 3d_5/2_ and Br 3d_3/2_ peaks appear at 70.89 and 71.82 eV, respectively. In contrast, in the co-melted glassy G3, these peaks are positively shifted to 71.12 and 72.10 eV, respectively, with a consistent spin–orbit splitting of approximately 0.98 eV. This upshift indicates a reduction in electron density around Br atoms in the glassy matrix, likely resulting from specific supramolecular interactions formed during co-melting, such as halogen bonding (*e.g.*, Br⋯F or Br⋯π) with the electron-deficient regions of DTPA2F, as well as enhanced dipole–dipole coupling. These interactions promote partial electron transfer from Br atoms in TPABr to the surrounding π-conjugated framework, thereby accounting for the observed increase in binding energy.^34^ These findings are consistent with the *in situ* FTIR results and further confirm that the co-melting process induces irreversible molecular reorganization and strengthens intermolecular coupling, which together contribute to the stabilized amorphous structure and improved thermal and mechanical robustness of G3.

Based on the above analysis, we propose a molecular-level construction mechanism for the vitrification process induced by host–guest co-melting, further elucidating the underlying molecular mechanism through analysis of the thermodynamic behavior and supramolecular interactions within these systems. As illustrated in Fig. 3g, the host and guest molecules co-melt to form a thermally mixed state in which local packing mismatches generate tensile strain.^35^ During the cooling process, these strains are progressively released as supramolecular interactions such as noncovalent binding and steric hindrance begin to dominate, resulting in a disordered yet mechanically robust amorphous network. This supramolecularly stabilized structure not only lowers the glass transition temperature or imparts glass-forming ability to otherwise non-glassy systems, but also enhances mechanical performance and enables excellent thermal reprocessability through reversible melting and remelting cycles.

### X-ray scintillation performance and stability of OGSSs

**Fig. 4:**
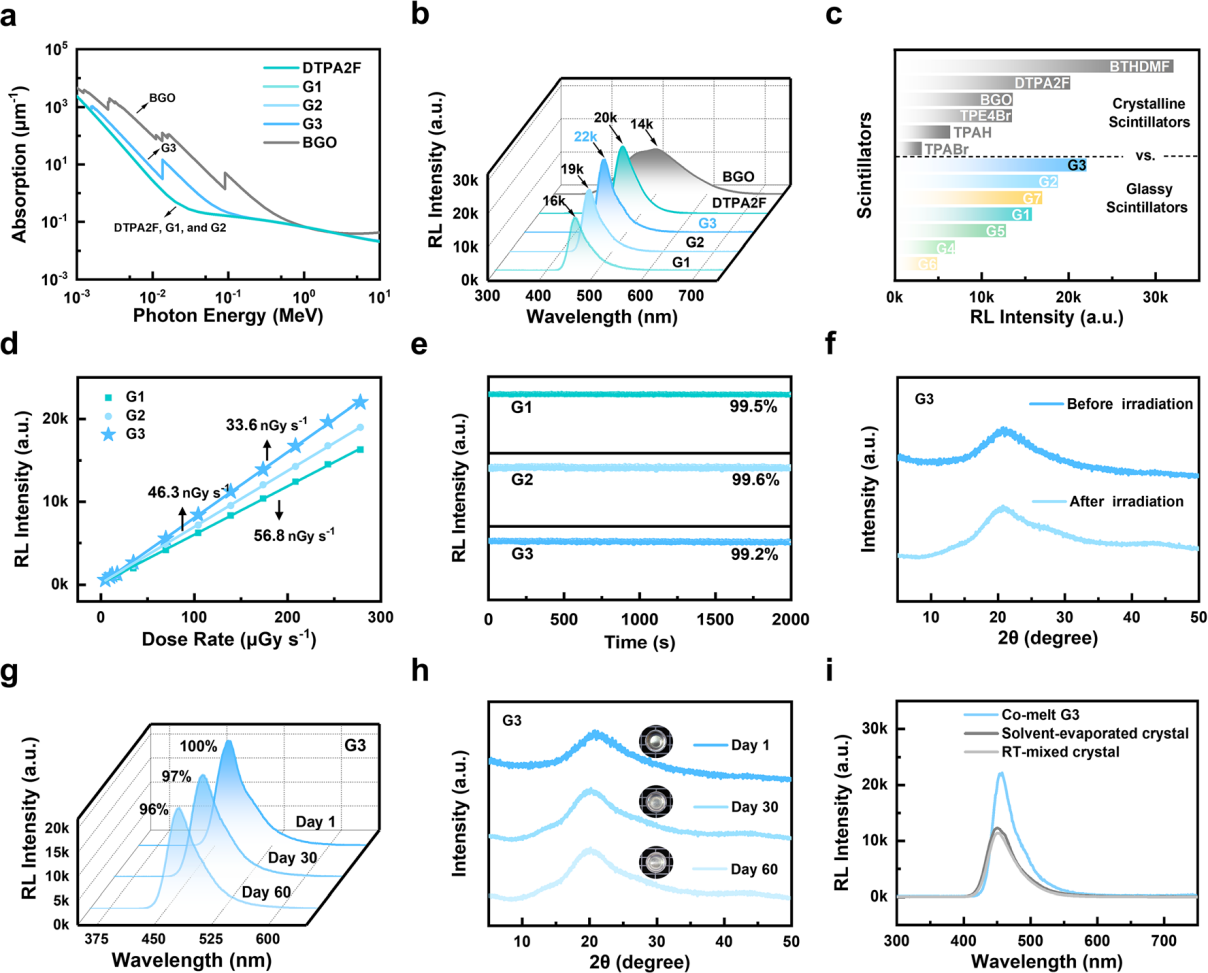
RL performance and stability of OGSSs. (a) X-ray absorption coefficients of DTPA2F, BGO, and G1–G3 as a function of photon energy. (b) RL spectra of G1–G3 and DTPA2F crystalline powder at an X-ray dose of 278 µGy s^−1^, with BGO as a reference. (c) Maximum RL intensities of crystalline samples and OGSSs including G1–G7. (d) Dose-rate-dependent RL intensities of G1–G3 with corresponding detection limits within an X-ray dose range of 4.58 to 278 µGy s^−1^. (e) RL intensity retention of G1–G3 during continuous X-ray exposure for 2000 s at an X-ray dose of 278 µGy s^−1^. (f) XRD patterns of G3 before and after X-ray irradiation. (g) RL spectra of G3 after storage for 1, 30, and 60 days at 35 °C and 80% RH at an X-ray dose of 278 µGy s^−1^. (h) XRD patterns of G3 after storage for 1, 30, and 60 days. (i) RL spectra of co-melted G3, physically mixed G3, and solvent-evaporated G3 at an X-ray dose of 278 µGy s^−1^.

Building on the structurally stabilized glass network and enhanced supramolecular interactions observed in the co-melted systems, the X-ray scintillation performance of the materials was subsequently investigated. The co-melting strategy enabled the efficient integration of heavy atoms into hot-exciton matrices without the need for complex synthetic modifications, thereby improving X-ray absorption and enabling tunable RL behavior. As shown in Fig. S31, the brominated compounds TPABr and TPE4Br exhibited X-ray attenuation nearly comparable to that of the commercial scintillator Bi_4_Ge_3_O_12_ (BGO), while TPAH, DTPA2F, and BTHDMF showed markedly weaker absorption. When TPABr was co-melted with either DTPA2F or BTHDMF, the resulting glasses G3 and G7 demonstrated significantly enhanced X-ray attenuation (Fig. 4a and S32). RL measurements at an X-ray dose of 278 µGy s^−1^ (Fig. S33) further showed that the hot-exciton molecules DTPA2F (20 200 a.u.), TPE4Br (13 500 a.u.), and BTHDMF (32 100 a.u.) exhibited higher luminescence than BGO (13 600 a.u.) and TPAH (6400 a.u.). In contrast, TPABr generated a relatively weak RL signal (3100 a.u.) despite its strong X-ray absorption, likely due to fluorescence quenching induced by bromine atoms.^36^ To achieve an optimal balance between X-ray absorption and RL, various mixing ratios were investigated. Although TPAH and TPABr can co-melt with DTPA2F at arbitrary mass ratios due to their compatible glass-forming abilities, RL performance depends strongly on composition. Using G3 as a representative example (Fig. S34), the highest RL intensity was observed at a 1 : 1 mass ratio of TPABr to DTPA2F; therefore, the same ratio was adopted for G2 to enable a meaningful comparison.

As shown in Fig. S35, the optimized RL intensities followed the trend: G3 (22 100 a.u.) > G2 (18 800 a.u.) > G7 (17 000 a.u.) > G1 (15 800 a.u.) > G5 (12 800 a.u.) > G4 (6930 a.u.) > G6 (4900 a.u.). The RL intensities of the hot-exciton crystals DTPA2F, TPE4Br, and BTHDMF were compared with those of their corresponding glassy forms (G1–G7). As shown in Fig. 4b, vitrification led to a 22% reduction in RL intensity for G1 compared to its crystalline counterpart, highlighting the luminescence loss typically associated with glass formation. Remarkably, G2 preserved 93% of the crystalline emission, while G3 not only avoided any performance degradation but also surpassed the crystalline DTPA2F, achieving 110% of its RL intensity. This represents a 41% enhancement relative to the vitrified G1, demonstrating that the co-melting strategy enables glass-state luminescence superior even to the original crystalline emitter. The TPE4Br- and BTHDMF-based systems exhibited varying degrees of RL retention after vitrification (Fig. S36). For the TPE4Br series, G5 retained 94% of the crystalline RL intensity, whereas G4 showed a more pronounced decrease, retaining only 51%. In the BTHDMF-based systems, G7 maintained 52% of the crystalline value, while G6 experienced a substantial drop, preserving just 15%. A complete summary of the maximum RL intensities for all crystalline and glassy samples is provided in Fig. 4c. To further assess scintillation performance, the detection limits of DTPA2F-based glasses were measured. As shown in Fig. 4d, Table S6 and Fig. S37, G1, G2, and G3 exhibited detection limits of 56.8, 46.3, and 33.6 nGy s^−1^, respectively, with G3 showing an approximate 40% reduction compared to G1. All values are well below the clinical X-ray diagnostic dose rate of 5.50 µGy s^−1^. Notably, the detection limit of G3 is only 1/164 of this clinical threshold and 1/15 of that of the benchmark commercial scintillator BGO (508.29 nGy s^−1^).^37^ In addition, the relative light yields of G1, G2, and G3, as determined in the SI and shown in Fig. S38, were calculated to be 28 004, 32 852, and 33 763 photons per MeV, respectively. Notably, the light yield of G3 is 4.22 times that of BGO (8000 photons per MeV), demonstrating its strong potential to replace conventional inorganic scintillators.

Additionally, the RL intensities of G1, G2, and G3 remained stable, retaining nearly 100% of their initial values after continuous X-ray irradiation for 2000 s at a dose rate of 278 µGy s^−1^ (Fig. 4e). And the environmental stability of the co-melted glasses was further examined. As shown in Fig. S39, optical photographs of representative samples (G1–G3) under daylight illumination after storage for 1, 30, and 60 days at 35 °C and 80% relative humidity (RH) reveal no evidence of crystallization or phase separation. The glasses maintain their transparency and appearance throughout the aging process, demonstrating excellent long-term stability under harsh environmental conditions. Further stability evaluations were conducted for the best-performing sample, G3. XRD analysis revealed negligible structural changes before and after X-ray irradiation (Fig. 4f). Moreover, as shown in Fig. 4g and h, G3 retained over 96% of its initial RL intensity after 60 days of ambient storage, and its XRD patterns consistently exhibited a broad halo without the emergence of crystalline peaks. These results demonstrate the outstanding RL stability and structural robustness of the OGSSs under long-term environmental exposure.^38^ Control experiments were also performed by either physically mixing DTPA2F and TPABr at room temperature or forming mixed crystals *via* solvent evaporation. As shown in Fig. 4i, neither approach resulted in any significant enhancement in RL intensity compared to the co-melted G3 sample, indicating that effective energy coupling between the two components requires high-temperature co-melting to be successfully established.

### Photophysical characterization and density functional theory mechanistic analysis

**Fig. 5:**
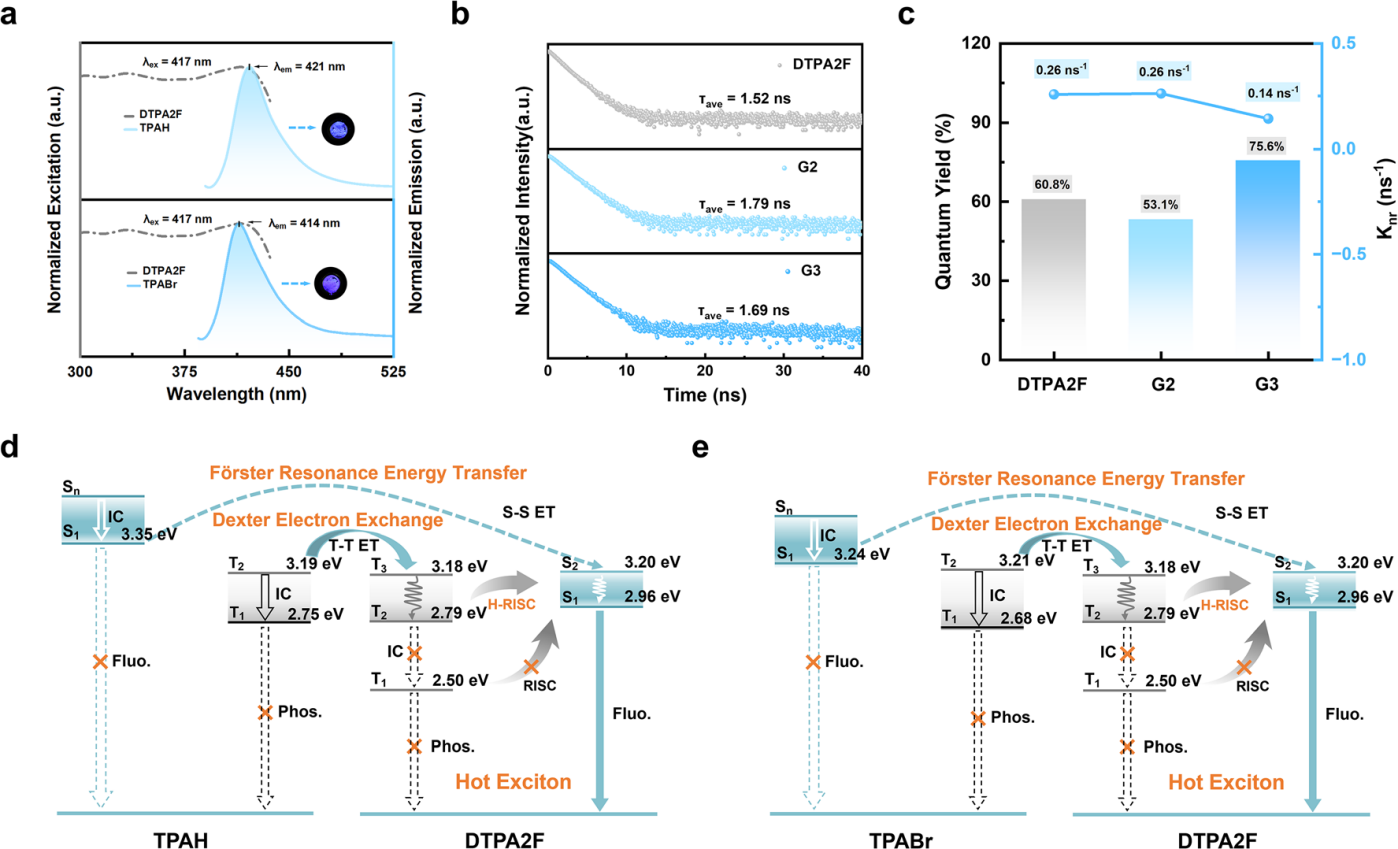
Photophysical processes and energy transfer of OGSSs. (a) Normalized excitation spectrum of DTPA2F and emission spectra of TPAH and TPABr. Insets show optical images of TPAH and TPABr powders under 365 nm UV light. (b) Time-resolved PL lifetime curves of DTPA2F, G2, and G3. (c) PLQY and *K*_nr_ of DTPA2F, G2, and G3. (d and e) Energy level diagrams and proposed photophysical processes in the TPAH–DTPA2F (G2) and TPABr–DTPA2F (G3) co-melting systems, involving FRET, Dexter electron exchange, reverse intersystem crossing (RISC), and hot-exciton channels.

The photophysical properties of the OGSSs were analyzed to elucidate the origins of their RL performance differences. As shown in Fig. 5a, the emission peaks of TPAH and TPABr are centered at 421 nm and 414 nm, respectively, while DTPA2F exhibits a strong excitation band at 417 nm. This pronounced spectral overlap facilitates favorable conditions for both FRET and Dexter exchange, which rely on dipole coupling and orbital overlap, respectively.^39–42^ The observed overlap suggests that efficient non-radiative energy transfer is feasible in the co-melted glass matrices, potentially contributing to their enhanced luminescence efficiency. In contrast, as shown in Fig. S40, the spectral overlap in G4–G7 systems is significantly weaker. In the TPE4Br-based systems (G4, G5), the host excitation occurs at 397 nm, which only partially overlaps with the emission bands of TPAH (421 nm) and TPABr (414 nm). In the BTHDMF-based systems (G6, G7), the situation is further exacerbated: although BTHDMF emits at 467 nm, this emission poorly aligns with the excitation bands of the guest molecules, resulting in suboptimal energy transfer conditions. As shown in Fig. S41–S43, G1–G3 exhibit red-shifted emission relative to pure DTPA2F, G4 and G5 are red-shifted compared to TPE4Br, and G6 and G7 show red-shifted emission relative to BTHDMF. In all cases, the emission profiles remain highly consistent with those of the corresponding hot-exciton molecules, supporting the occurrence of intermolecular energy transfer within the co-melting glass matrices.^43–46^

Further insight into the excited-state dynamics was obtained through fluorescence lifetime measurements. As shown in Fig. 5b, DTPA2F exhibits a lifetime of 1.52 ns, while the co-melting glasses G2 and G3 show slightly longer lifetimes of 1.79 and 1.69 ns, respectively, comparable to that of G1 (1.66 ns; Fig. S44). The fluorescent hosts TPAH and TPABr display lifetimes of 1.88 and 0.67 ns, respectively (Fig. S45). In the TPE4Br system (Fig. S46a), G4 and G5 exhibit extended lifetimes of 3.87 and 3.34 ns, both longer than that of pristine TPE4Br (2.15 ns). In contrast, for the BTHDMF system (Fig. S46b), the lifetimes of G6 and G7 decrease to 4.21 and 3.83 ns, compared to 5.67 ns for pure BTHDMF. These results confirm that all co-melting glasses retain nanosecond-scale emission lifetimes, thus preserving the characteristic excited-state behavior of hot-exciton materials. Notably, the short PL lifetime of the scintillator is a key advantage, making it particularly promising for PET-related imaging applications where fast temporal resolution is crucial.^47^

Meanwhile, to confirm the role of energy transfer in enhancing RL performance, the PLQY and non-radiative decay rate (*K*_nr_) of each component and its corresponding co-melting OGSS were systematically compared. As shown in Fig. S47, the PLQY of crystalline DTPA2F is 60.8%, which decreases to 49.8% upon vitrification into G1, indicating a decline in excited-state stability due to structural disorder. Remarkably, after co-melting with low-*T*_g_ fluorescent molecules TPAH and TPABr, the PLQYs of G2 and G3 increase to 53.1% and 75.6%, respectively (Fig. 5c and S48), with G3 showing a significant enhancement. Correspondingly, the *K*_nr_ decreases from 0.26 ns^−1^ (G1) to 0.14 ns^−1^ (G3), clearly demonstrating that the co-melting strategy facilitates efficient energy transfer while suppressing non-radiative pathways, thereby boosting radiative efficiency. It is noteworthy that the PLQYs of TPAH and TPABr alone are only 33.5% and 3.47% (Fig. S49), respectively. Their incorporation into the co-melting glass markedly enhances overall luminescence, further supporting their role as energy hosts that transfer excitation energy to DTPA2F, the hot-exciton acceptor. Similarly, in the TPE4Br-based system, G4 and G5 exhibit increased PLQYs of 62.3% and 69.4%, respectively, with *K*_nr_ values reduced from 0.18 to 0.10 and 0.09 ns^−1^ (Fig. S50 and S51), confirming the broad applicability of the strategy. In contrast, BTHDMF exhibits an intrinsically high PLQY of 95.2%, which slightly decreases to 78.4% and 79.2% in G6 and G7 (Fig. S52 and S53), with *K*_nr_ remaining low (0.05–0.06 ns^−1^), suggesting limited contribution from energy transfer in this case.

DFT calculations were further carried out to analyze the electronic structures of TPAH, TPABr, DTPA2F, TPE4Br, and BTHDMF, with the goal of elucidating the origin and directionality of these transfer processes. Correspondingly, the calculated singlet and triplet energy levels of these molecules are summarized in Tables S7 and S8, providing further insight into the exciton dynamics. As shown in the singlet and triplet energy diagrams (Fig. 5d and e), the lowest excited singlet states (S_1_) of TPAH (3.35 eV) and TPABr (3.24 eV) are 0.39 and 0.28 eV higher than that of DTPA2F (2.96 eV), respectively. This moderate energy offset provides the driving force for singlet–singlet FRET from the host to the guest.^48^ Meanwhile, the triplet states (T) of TPAH (T_1_ = 2.75 eV) and TPABr (T_1_ = 2.68 eV) also lie above that of DTPA2F (T_1_ = 2.50 eV), enabling Dexter-type triplet–triplet energy transfer (T–T ET).^40^ In addition, the participation of higher-lying triplet states (T_2_/T_3_) in DTPA2F and their efficient hot-exciton reverse intersystem crossing (H-RISC) pathways further facilitates triplet harvesting and exciton utilization, thereby ensuring effective energy transfer and radiative recombination in the co-melting systems. For TPE4Br, the T_1_ energy (2.53 eV) is lower than those of TPAH (2.75 eV) and TPABr (2.68 eV), establishing a favorable energetic alignment for triplet-mediated Dexter electron exchange (T–T ET) (Fig. S54). By comparison, BTHDMF shows much lower excited-state energies, with S_1_ = 1.79 eV and T_1_ = 1.33 eV, approximately 1.4 eV below those of the host molecules (TPAH/TPABr). Although this large offset formally satisfies the driving-force requirement for energy transfer, it simultaneously increases the probability of nonradiative decay, thereby limiting the overall transfer efficiency (Fig. S55). By leveraging these favorable energetic alignments and the participation of higher-lying triplet states, the OGSSs are expected to promote hot-exciton harvesting *via* H-RISC, which helps reduce energy losses through conventional triplet-state decay channels.

**Fig. 6:**
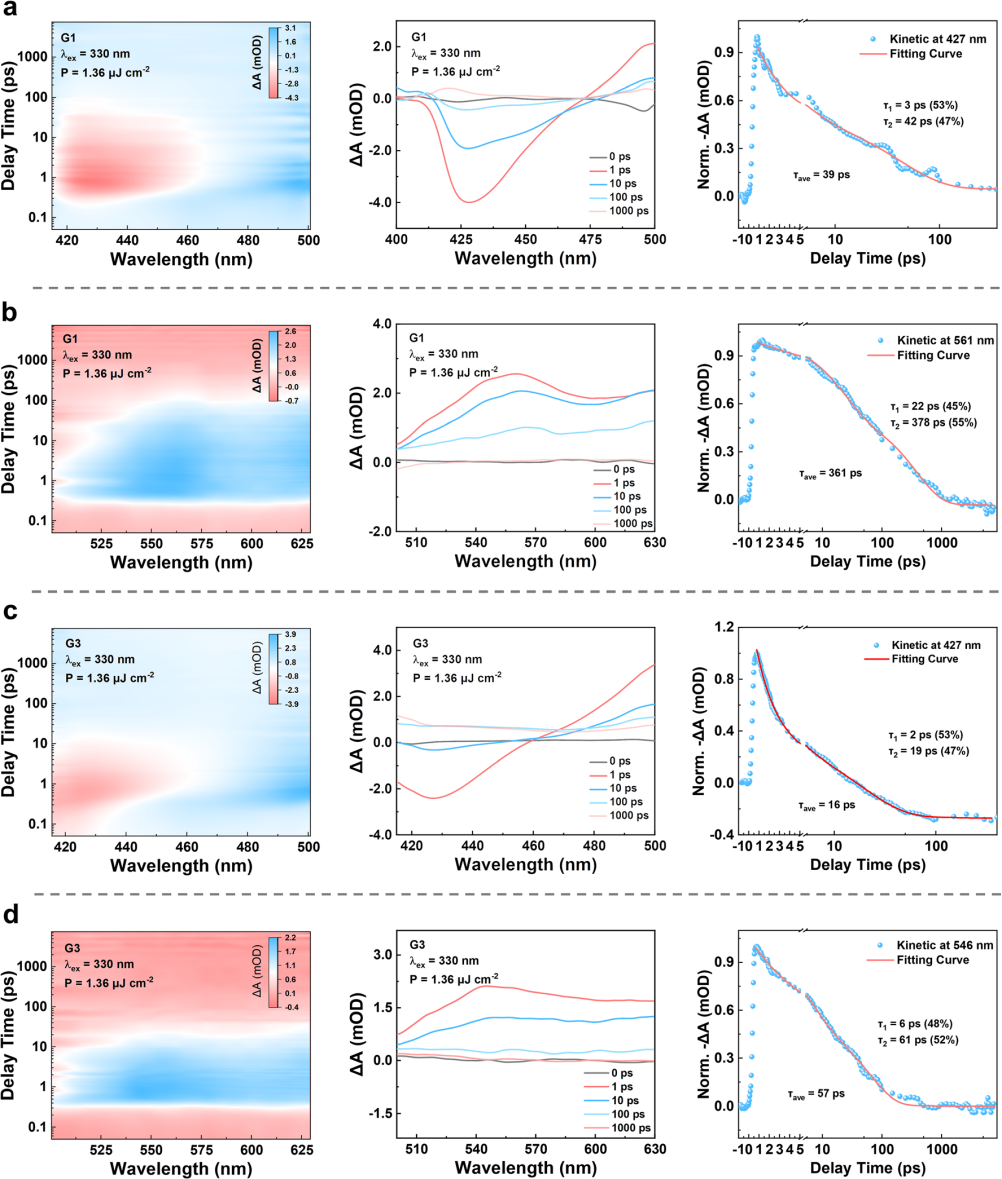
Transient absorption processes of G1 and G3. (a and b) Transient absorption of G1 in the 415–625 nm region, including the pseudocolor TA plots, GSB at 427 nm, and ESA at 561 nm with corresponding kinetic decays. (c and d) Transient absorption of G3 in the 415–625 nm region, including the pseudocolor TA plots, GSB at 427 nm and ESA at 546 nm with corresponding kinetic decays. All measurements were performed under 330 nm excitation with a fluence of 1.36 µJ cm^−2^.

To experimentally validate the exciton dynamics inferred from DFT calculations, particularly the feasibility of host-to-guest energy transfer and hot-exciton harvesting through higher triplet states, we performed femtosecond transient absorption (fs-TA) spectroscopy on the representative co-melting systems G1 and G3.^49^ As shown in Fig. 6, the measurements were conducted over the 415 to 625 nm spectral range under 330 nm excitation with a fluence of 1.36 µJ cm^−2^, enabling real-time tracking of singlet and triplet state transitions.^50^ The kinetic traces were fitted using a biexponential decay model to capture both ultrafast and intermediate deactivation processes commonly observed in hot-exciton systems. In the case of G1, a prominent ground-state bleach (GSB) signal at 427 nm, corresponding to the depopulation of the S_0_ → S_1_ transition, rises within one picosecond and decays with a lifetime of 39 ps, reflecting the deactivation of the S_1_ state. Simultaneously, an excited-state absorption (ESA) band at 561 nm, attributed to T_1_ → T_*n*_ transitions, shows biexponential decay with fitted lifetimes of 361 ps. The presence of a longer decay component suggests limited triplet accumulation. However, no extended ESA signals beyond the nanosecond regime were detected, indicating that the S_1_ → T_1_ intersystem crossing remains insignificant.

In G3, both the GSB and ESA features exhibit significantly shorter lifetimes. The decay at 427 nm is reduced to 17 ps, and the ESA at 546 nm fades with a fitted lifetime of 57 ps. Meanwhile, the ESA maximum shows a 15 nm blue shift from 561 nm in G1 to 546 nm in G3, indicating a higher-energy excited-state absorption process. These spectral and kinetic evolutions indicate accelerated relaxation and radiative recombination of excitons in G3, accompanied by reduced involvement of intermediate trap states or delayed emission pathways. This behavior can be explained by the efficient energy transfer from the host to the guest, which enables direct population of the S_1_ state in the guest while avoiding losses through low-energy traps or delayed hot-exciton recombination. Furthermore, the absence of persistent ESA signals supports the proposed hot-exciton mechanism in which high-lying triplet states undergo fast reverse intersystem crossing back to S_1_ without being funneled into the T_1_ state. Together with the calculated energy alignments, these transient results confirm that the S_1_ → T_1_ intersystem crossing is effectively suppressed in both systems and demonstrate that G3 retains the hot-exciton characteristics.

Moreover, the accuracy of the energy level analysis was validated by measuring the PL spectra of the five organic molecules at room temperature (298 K; Fig. S56) and at low temperature (77 K; Fig. S57).^7^ At 298 K, the emission peak energies of TPAH, TPABr, DTPA2F, TPE4Br, and BTHDMF were 3.10, 3.09, 2.97, 3.11, and 2.58 eV, respectively. At 77 K, the corresponding values shifted slightly to 3.22, 3.13, 2.93, 3.06, and 2.56 eV. These experimental results are consistent with the DFT-calculated energy levels and exhibit parallel trends across all compounds, confirming the reliability of the energy-level alignment. The PL data further validate the predicted excited-state configurations and provide strong experimental evidence for efficient host-to-guest energy transfer in the co-melting glass systems, where the fluorescent molecules serve as hosts and the hot-exciton emitters serve as guests. Within the amorphous glassy matrix, supramolecular interactions create close spatial proximity and favorable electronic coupling between the two components. These interactions facilitate exciton migration and help suppress non-radiative losses, thereby enhancing overall luminescence performance.

### X-ray imaging performance and applications of the G3 scintillator screen

**Fig. 7:**
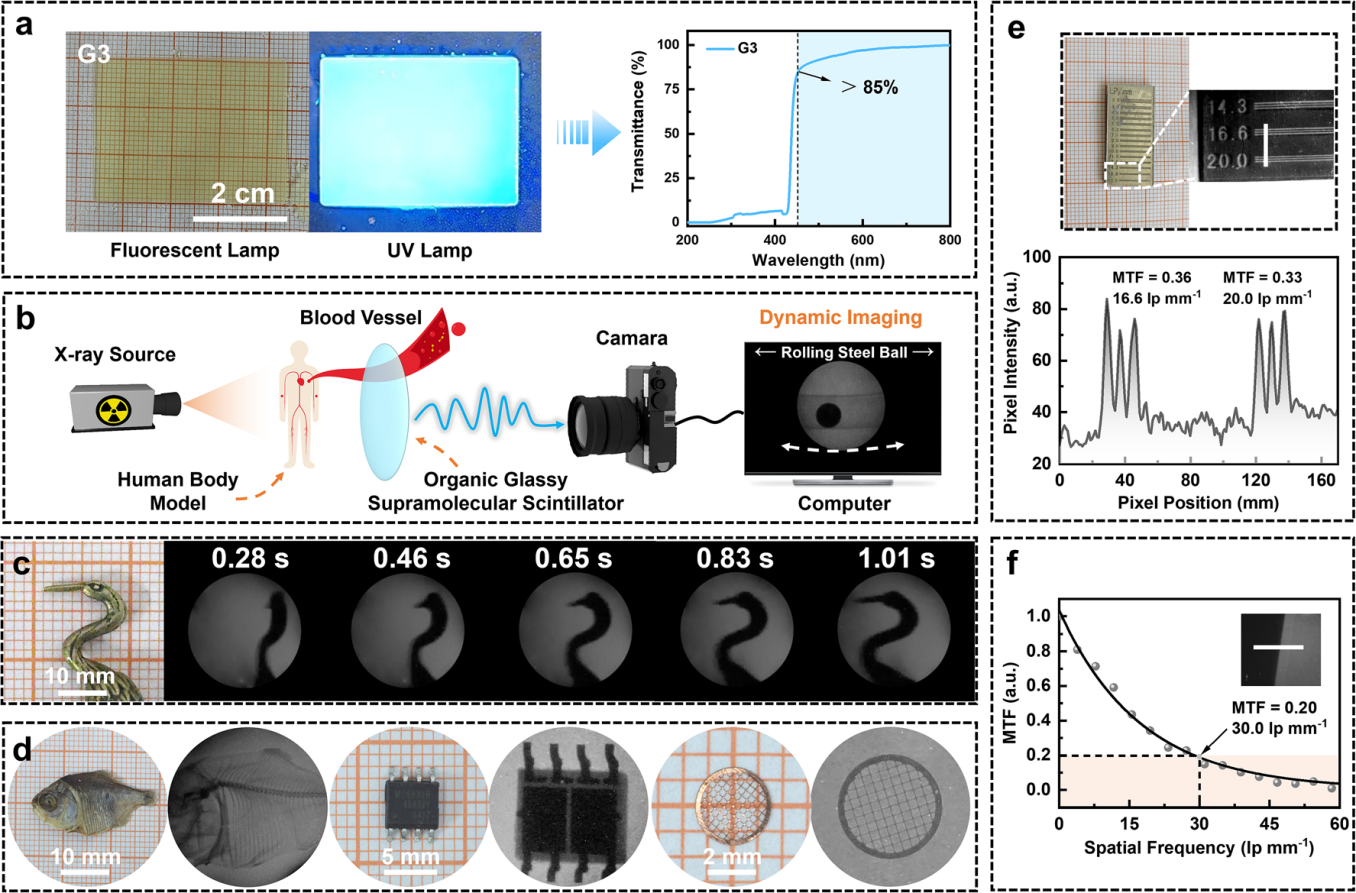
Applications of the G3 scintillator screen in high-resolution and dynamic X-ray imaging. (a) Photographs of the G3 scintillator screen under ambient and UV light, and its transmittance spectrum. (b) Schematic diagram of the dynamic X-ray imaging setup using a glassy scintillator and a human-body blood flow model. (c) Time-sequenced dynamic X-ray images of a bird-shaped model. (d) Static X-ray images of representative biological and electronic samples, including a dried fish, a circuit board, and copper mesh. (e) X-ray image of a resolution test pattern and the corresponding pixel intensity profile. (f) Modulation transfer function (MTF) curve obtained by the edge method. Unless otherwise noted, X-ray imaging was performed at an X-ray dose of 278 µGy s^−1^.

To evaluate the X-ray imaging performance of the best-performing organic glassy supramolecular scintillator G3, a large-area glassy screen with a surface area of 12 cm^2^ was fabricated *via* a facile melt-quenching process, in which the molten co-melting material was directly cast into a mold (Fig. 7a). The resulting screen exhibited a smooth and uniform surface morphology, with exceptional flatness that helps reduce optical crosstalk during image acquisition and thereby enhances imaging clarity. As confirmed in Fig. 7a, the screen shows an optical transmittance exceeding 85% across the visible spectrum. This high transparency minimizes light scattering, significantly improves light output efficiency, and enhances the suitability of materials for advanced photonic applications.^51^

Building upon these favorable optical properties, the X-ray imaging performance of the G3-based glass scintillator was systematically evaluated through both dynamic and static imaging experiments. As illustrated in Fig. 7b and S58, a custom-designed dynamic imaging setup was developed to simulate physiological processes such as blood flow within a human body model. The computer screen shows dynamic X-ray images of a steel ball moving through a water-filled tube, serving as a surrogate for thrombus motion (Movie S1). Benefiting from the ultrafast response time of the scintillator, this setup enables accurate tracking of fluid-like dynamics. Fig. 7c and S59 present a time-sequential X-ray imaging series of a dancing bird-shaped object, with each frame clearly resolved and free of motion blur or ghosting artifacts (Movie S2).

In addition to dynamic tracking, high-resolution static imaging was also investigated. As shown in Fig. 7d, the G3 scintillator enables clear visualization of fine internal features in diverse specimens, including the skeletal structure of a dried fish, intricate circuitry in an electronic chip, and the microstructure of a copper mesh. To further assess its spatial resolution, a line-pair phantom test was performed, as shown in Fig. 7e, where a clear resolution of 20 lp mm^−1^ was achieved. Pixel intensity profiles extracted using ImageJ revealed MTF values of 0.356 and 0.326 at 16.6 and 20 lp mm^−1^, respectively, indicating excellent spatial resolution. In addition, quantitative analysis using the edge method (Fig. 7f and S60) yielded an MTF = 0.2 spatial resolution of 30.0 lp mm^−1^, outperforming most previously reported organic scintillators (Table S9). These results collectively demonstrate the excellent imaging fidelity, high spatial resolution, and fast scintillation response of the co-melted hot-exciton OGSSs, positioning them as strong candidates for dynamic biomedical diagnostics and high-precision radiographic imaging.

## Conclusions

We have developed a host–guest co-melting strategy for constructing color-tunable OGSSs with excellent processability, stability, and mechanical integrity. By integrating heavy-atom-containing fluorescent host molecules (TPABr) with hot-exciton guest emitters (DTPA2F, TPE4Br, and BTHDMF), this solvent-free approach enables the direct fabrication of large-area transparent scintillator screens through a simple melt-quenching process. The resulting materials exhibit tunable emission colors, homogeneous glassy morphology, and strong compatibility with screen-level integration, making them highly suitable for both static and dynamic X-ray imaging applications. Notably, the optimized co-melted glass (G3) demonstrates ∼51% higher Young's modulus, ∼116% greater hardness, ∼41% stronger RL intensity, a high PLQY of 75.6%, an ultrafast PL lifetime of 1.69 ns, and a relative light yield of 33 763 photons per MeV. The enhanced performance originates from efficient X-ray absorption and optimized host–guest energy transfer. In addition, supramolecular interactions stabilize molecular packing within a rigid microenvironment, effectively suppressing nonradiative decay. Furthermore, the co-melted glass achieves a high imaging resolution of 30.0 lp mm^−1^ and effectively eliminates afterglow artifacts during dynamic X-ray imaging, such as in vascular models and small biological specimens. The co-melting strategy eliminates the need for complex synthesis or post-treatment, offering a scalable and generalizable route to high-performance scintillators. Overall, this work demonstrates a practical supramolecular design pathway toward next-generation organic scintillation materials with strong potential for commercialization and deployment in diverse radiation imaging and detection scenarios.

## Author contributions

Yuan-Ji Ye: conceptualization, data curation, formal analysis, investigation, methodology, software, visualization, original draft preparation, review & editing. Xiang-Long Wei, Xi Yang, Yu-Dong Chen and Ming-Cen Weng: data curation. Hong-Ming Chen and Mei-Jin Lin: conceptualization, funding acquisition, resources, supervision, validation, original draft preparation, review & editing.

## Conflicts of interest

There are no conflicts to declare.

## Supplementary Material

SC-OLF-D6SC00159A-s001

SC-OLF-D6SC00159A-s002

## Data Availability

The data that support the plots within this paper and other findings of this study are available from the corresponding author upon reasonable request. CCDC Structures 2428812 (TPAH) and 2428818 (TPABr) are available in the supplementary information (SI).^52*a*,*b*^ CCDC Structure 2357920 (DTPA2F) was previously published in ref. 8. CCDC Structure 2435305 (TPE4Br) was previously deposited as CCDC Structure 1948856 and published in ref. 20. Crystal data was recollected for this compound and calculations are based on the recollected structure.^52*c*^ CCDC Structure 2435306 (BTHDMF) was previously published as CCDC Structure 1856032 and published in ref. 19. Crystal data was recollected for this compound and calculations are based on the recollected structure.^52*d*^ Supplementary information (SI): experimental procedures, characterization, and additional spectra and other relevant data. See DOI: https://doi.org/10.1039/d6sc00159a.
