# Supplementary material for: Color-tunable hot-exciton organic glassy supramolecular scintillators enabled by host–guest co-melting
Source: Chem Sci. 2026 Mar 5;17(18):9297–310. doi: 10.1039/d6sc00159a (PMC13007790; doi:10.1039/d6sc00159a)
Supplement: SC-017-D6SC00159A-s001 [file SC-017-D6SC00159A-s001.pdf]

Supplementary Information (SI) for Chemical Science.
This journal is © The Royal Society of Chemistry 2026

SUPPORTING INFORMATION

Color-Tunable Hot-Exciton Organic Glassy Supramolecular Scintillators Enabled by Host-Guest Co-Melting

Yuan-Ji Ye,[a] Xiang-Long Wei,[c] Xi Yang,[c] Yu-Dong Chen,[b] Ming-Cen Weng,[d] Hong-Ming Chen[c] and Mei-Jin Lin*[abc]*

[a] *College of Chemical Engineering, Fuzhou University, Fuzhou, 350116, P.R. China.*
E-mail: meijin_lin@fzu.edu.cn.

[b] *College of Chemistry, Fuzhou University, Fuzhou, 350116, P.R. China.*

[c] *College of Materials Science and Engineering, Fuzhou University, Fuzhou, 350116, P.R. China.*
E-mail: chm@fzu.edu.cn.

[d] *Institute of Biology and Chemistry, Fujian University of Technology, Fuzhou, 350118, P.R. China.*

Table of Contents

1. Materials and Synthesis 3
2. Preparation 6
3. Characterizations 7
4. Single-crystal Structure Analysis 9
5. Quantum Chemical Calculations 9
6. Correlation Formulas and Analysis Methods 10
7. Related Figures and Tables 11
8. References 49

1. Materials and Synthesis

Materials and General Methods: All reagents and solvents were purchased from Energy Chemical or Innochem and used without further purification unless otherwise stated. Solvents used for chromatographic separation and post-reaction workup were of reagent grade or higher. All synthetic procedures were performed under an argon atmosphere in oven-dried glassware unless otherwise specified. Reaction temperatures were monitored using external thermometers.

Synthesis of 2',5'-difluoro-N,N-diphenyl-[1,1'-biphenyl]-4-amine (TPAH): In a two-neck round-bottom flask, 4-bromodiphenylamine (2.91 g, 10 mmol), diphenylamine boronic acid (2.95 g, 11 mmol), and tetrakis(triphenylphosphine)palladium (0.58 g, 0.5 mmol) were combined. A 2 M aqueous potassium carbonate solution (25 mL) and toluene (100 mL) were added under an argon atmosphere. The reaction mixture was refluxed for 12 h, cooled to room temperature, and extracted with dichloromethane (3 × 50 mL). The combined organic layers were dried over anhydrous magnesium sulfate, filtered, and concentrated under reduced pressure. The crude product was purified by column chromatography on silica gel using dichloromethane/petroleum ether (1:8, v/v) as the eluent. The final product was obtained as colorless crystals after recrystallization, yielding 4.02 g (98%). The synthesis employed a solvent mixture of dichloromethane and petroleum ether in a (1/8, v/v) ratio. ^{1}H NMR (400 MHz, $CDCl_3$) δ 7.45 (d, J = 8.4 Hz, 2H), 7.32 (t, J = 7.3 Hz, 4H), 7.19 (d, J = 7.9 Hz, 7H), 7.10 (t, J = 7.2 Hz, 3H), 7.04 – 6.92 (m, 1H).

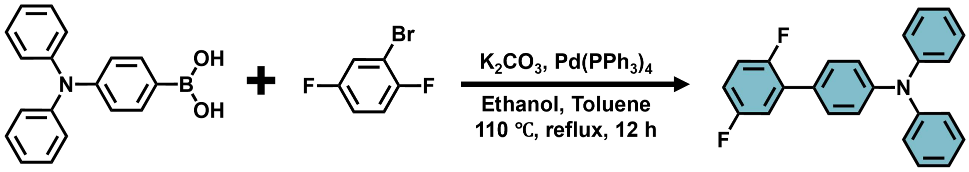

Figure S1. Synthesis route of TPAH.

Synthesis of 4'-bromo-2',5'-difluoro-N,N-diphenyl-[1,1'-biphenyl]-4-amine (TPABr): The compound TPABr was synthesized following a procedure similar to that used for TPAH. In a two-neck round-bottom flask, 1,4-dibromo-2,5-difluorobenzene (2.97g, 11 mmol), (4-(diphenylamino)phenyl) boronic acid (2.89 g, 10 mmol) and tetrakis(triphenylphosphine)palladium (0.58 g, 0.5 mmol) were combined. Subsequently, a 2M potassium carbonate solution (25 mL), and toluene

(100 mL) were added to the flask using syringes. The reaction mixture was refluxed for 12 h and then allowed to cool to room temperature. The mixture was then extracted three times with dichloromethane, and the combined organic layers were dried over anhydrous magnesium sulfate. After filtration and evaporation, the crude product was further purified by silica-gel column chromatography using dichloromethane/petroleum ether (1/8, v/v) as the eluent. The final product was obtained by recrystallization from a mixed solvent of dichloromethane and ethanol, yielding a colorless crystal (5.2 g, 86% combined yield). ^{1}H NMR (400 MHz, $CDCl_3$) δ 7.44 – 7.35 (m, 3H), 7.34 – 7.27 (m, 5H), 7.23 (t, J = 7.2 Hz, 1H), 7.20 – 7.14 (m, 4H), 7.13 – 7.06 (m, 3H).

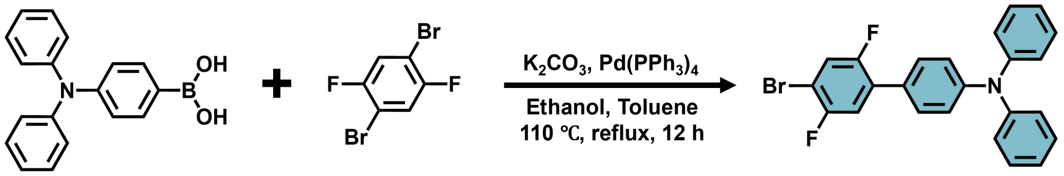

Figure S2. Synthesis route of TPABr.

Synthesis of 2',5'-difluoro-N4,N4, N4",N4"-tetraphenyl-[1,1':4',1"-terphenyl]-4,4"-diamine (DTPA2F): In a two-neck round-bottom flask, 1,4-dibromo-2,5-difluorobenzene (2.7g, 10 mmol), (4-(diphenylamino)phenyl) boronic acid (6.36 g, 22 mmol) and tetrakis(triphenylphosphine)palladium (0.58 g, 0.5 mmol) were combined. Subsequently, a 2M potassium carbonate solution (16 mL), ethanol (16 mL), and toluene (50 mL) were added to the flask using syringes. The reaction mixture was refluxed for 24 h and then allowed to cool to room temperature. The mixture was then extracted three times with dichloromethane, and the combined organic layers were dried over anhydrous magnesium sulfate. After filtration and evaporation, the crude product was further purified by silica-gel column chromatography using dichloromethane/petroleum ether (1/8, v/v) as the eluent. The final product was obtained by recrystallization from a mixed solvent of dichloromethane and ethanol, yielding a white solid (5.2 g, 86% combined yield). ^{1}H NMR (400 MHz, $CDCl_3$) δ 7.45 (d, J = 8.9 Hz, 3H), 7.32 – 7.26 (m, 10H), 7.22 (t, J = 9.0 Hz, 2H), 7.18 – 7.11 (m, 10H), 7.06 (tt, J = 7.0, 1.2 Hz, 4H).

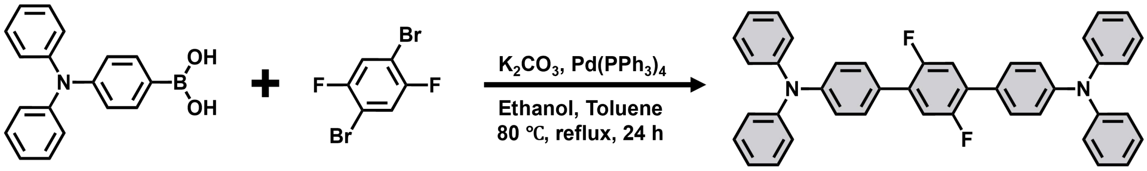

Figure S3. Synthesis route of DTPA2F.

Synthesis of 4,7-bis (9,9-dimethyl-9H-fluoren-2-yl) benzo [c] [1,2,5] thiadiazole (BTHDMF): 9,9-Dimethyl-9H-fluoren-2-ylboronic acid (524 mg, 2.2 mmol), 4,7-dibromobenzo[c][1,2,5]thiadiazole (294 mg, 1.0 mmol), and tetrakis(triphenylphosphine)palladium (16 mg, 0.0144 mmol) were added to a 100 mL three-neck round-bottom flask under a nitrogen atmosphere. A mixture of ethanol and toluene (1:2, v/v; total volume 21 mL) and a 2 M aqueous solution of potassium carbonate (6 mL) were introduced sequentially. The reaction mixture was stirred and refluxed at 110 °C for 5 h. After cooling to room temperature, the mixture was diluted with diethyl ether and washed with brine. The organic layer was dried over anhydrous magnesium sulfate, filtered, and concentrated under reduced pressure. The crude product was purified by silica gel flash column chromatography (dichloromethane/petroleum ether = 1:10, v/v), followed by recrystallization from dichloromethane/ethanol to afford the target compound as a green solid. Yield: 72%. ^{1}H NMR (500 MHz, CHLOROFORM-D) δ 8.06 (dd, J = 1.6, 0.6 Hz, 2H), 8.02 (dd, J = 7.9, 1.7 Hz, 2H), 7.91 (dd, J = 7.9, 0.6 Hz, 2H), 7.90 (s, 2H), 7.83 – 7.79 (m, 2H), 7.51 – 7.48 (m, 2H), 7.42 – 7.35 (m, 4H), 1.61 (s, 12H).

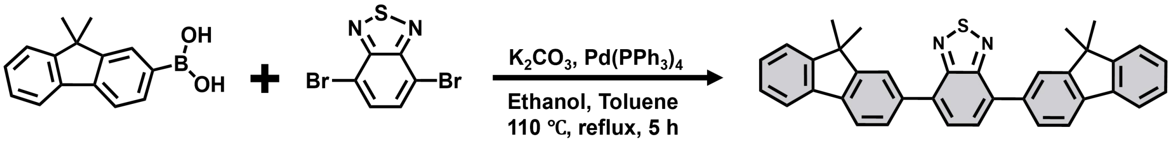

Figure S4. Synthesis route of BTHDMF.

Synthesis of 1,1,2,2-Tetra(4-bromophenyl)ethene (TPE4Br): TPE4Br was synthesized following a modified literature procedure using 4,4'-dibromobenzophenone as the starting material. After completion of the reaction, the crude product was purified by column chromatography to afford a white solid in 80% yield. ^{1}H NMR (400 MHz, $CDCl_3$) δ 7.27 (s, 4H), 6.84 (d, J = 8.0 Hz, 9H), 1.26 (s, 1H).

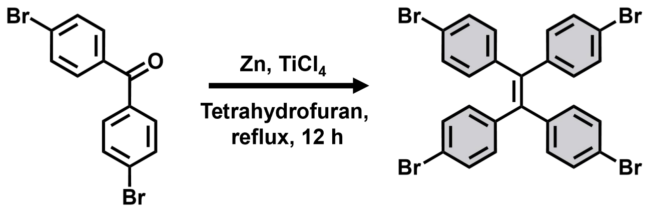

Figure S5. Synthesis route of TPE4Br.

2. Preparation

Growth of single crystals: Single crystals of TPAH, TPABr, DTPA2F, TPE4Br, and BTHDMF were grown via the saturated solution evaporation method. Each compound was first dissolved in dichloromethane to form a saturated solution, followed by the slow layering of ethanol. Crystallization occurred under ambient temperature and pressure. The resulting crystals of TPAH, TPABr, DTPA2F, and TPE4Br were colorless and transparent, while BTHDMF crystals appeared yellowish green. Morphologically, the crystals of TPAH, TPABr, and BTHDMF were block-shaped, whereas DTPA2F and TPE4Br formed rod-like crystals.

Preparation of DTPA2F (G1) glassy supramolecular scintillators: The G1 transparent screen was prepared using a simple melt-quenching technique. 100 mg of DTPA2F compound were placed in a beaker and heated to 220 °C until the material melted and all bubbles disappeared. The molten compound was then poured into a mold, and the DTPA2F transparent screen was formed by natural cooling at room temperature.

Preparation of TPAH-DTPA2F (G2) glassy supramolecular scintillators: The G2 transparent screen was prepared via a melt-quenching method. A 1:1 mass mixture of TPAH and DTPA2F (total 100 mg) was placed in a beaker and heated to 150 °C until fully molten and free of bubbles. The melt was then poured into a mold and cooled naturally to room temperature to form the transparent glass.

Preparation of TPABr-DTPA2F (G3) glassy supramolecular scintillators: A 1:1 mass mixture of TPABr and DTPA2F (total 100 mg) was melted at 150 °C in a beaker until clear and homogeneous. The molten mixture was then cast into a mold and allowed to cool at room temperature to afford the G3 transparent screen.

Preparation of TPAH-TPE4Br (G4) glassy supramolecular scintillators: The G4 transparent screen was obtained by melting a 1:1 mass mixture of TPAH and TPE4Br (100 mg total) at 200 °C. After complete melting and degassing, the material was poured into a mold and cooled to room temperature to yield a transparent glass.

Preparation of TPABr-TPE4Br (G5) glassy supramolecular scintillators: A mixture of TPABr and TPE4Br (1:1 mass ratio, total 100 mg) was heated to 200 °C until fully molten and bubble-free. The melt was poured into a mold and allowed to cool naturally at room temperature to form the transparent screen G5.

Preparation of TPAH-BTHDMF (G6) glassy supramolecular scintillators: The G6 transparent screen was prepared from a 2:1 mass mixture of TPAH and BTHDMF (total 100 mg) by heating at 200 °C until melted and clear. The resulting melt was poured into a mold and cooled to room temperature to afford a yellow transparent glass.

Preparation of TPABr-BTHDMF (G7) glassy supramolecular scintillators: A 2:1 mass mixture of TPABr and BTHDMF (100 mg total) was melted at 200 °C in a beaker. After removing bubbles, the melt was cast into a mold and cooled at room temperature to form the transparent G7 glass.

Preparation of TPABr–DTPA2F (G3) glassy supramolecular scintillator screen: A 1:1 mass mixture of TPABr and DTPA2F (totaling 800 mg) was heated to 150 °C in a glass beaker until fully melted into a clear and homogeneous liquid. The molten mixture was then cast into a flat mold and allowed to cool naturally at room temperature, forming a uniform, transparent glassy scintillator screen with a surface area of approximately 12 cm^2.

3. Characterizations

^{1}H NMR measurements were carried out on a Bruker Avance 400 spectrometer at resonant frequencies of 400 MHz or a JEOL JNM-ECZ500R/S1 spectrometer at resonant frequencies of 500 MHz, using Chloroform-d as solvent.

The crystallography data of TPAH, TPABr, DTPA2F, TPE4Br, and BTHDMF were collected by Rigaku-AFC11 equipped with a Rigaku Saturn CCD area detector (Bruker-SMART). The X-ray source was a graphic-monochromatized Mo-Kα radiation (λ = 0. 71073 Å) operating at 293 K under a flow of liquid nitrogen. Crystallographic data are available as publication numbers CCDC no. 2428812 (TPAH), no.2428818 (TPABr), no. 2357920 (DTPA2F), no. 2435305 (TPE4Br) and 2435306 (BTHDMF) from the Cambridge Crystallographic Data Centre, which can be accessed free of charge at: www.ccdc.cam.ac.uk/data_request/cif.

Powder X-ray diffraction (PXRD) patterns were recorded by a Rigaku Mini Flex-II X-ray diffractometer with Cu-Kα radiation (λ = 1.54184 Å).

Thermal stability was assessed using thermogravimetric analyzer (TGA) operated at 10 °C min^{-1} from 30 to 600 °C under a nitrogen atmosphere on a NETZSCH STA 449F5.

Differential scanning calorimetry (DSC) analysis was conducted on a NETZSCH DSC214 at a heating rate of 10 °C min^{-1} under nitrogen atmosphere from 20 to 300 °C, with the glass transition temperature (T_g) determined from the midpoint of the thermal transition in the second heating cycle.

X-ray photoelectron spectroscopy (XPS) measurements were performed using a Thermo Scientific™ K-Alpha spectrometer.

Energy dispersive X-ray spectroscopy (EDS) of single crystals was characterized with a SU8000 scanning electron microscope (SEM).

Nanoindentation measurements were performed using a TIPREMIER nanoindenter. All tests were conducted under ambient conditions with a predefined loading rate, hold segment, and unloading sequence. The indentation depth and load–displacement curves were recorded and analyzed using the Multiple Curve Analysis module.

In situ Fourier-transform infrared (FT-IR) spectra were recorded on a Thermo Fisher Scientific iS50 spectrometer in the range of 500–4000 cm^{-1} during the heating and subsequent cooling process.

Photoluminescence (PL) measurements were conducted on an Edinburgh Instruments FLS980 spectrofluorometer.

The photoluminescence quantum yield (PLQY) for crystalline powders was measured using a Horiba FluoroMax-4 spectrometer. The PLQY for single crystals glass or co-melt glass was determined using a Hamamatsu Absolute PL Quantum Yield Spectrometer C11347 Quantaurus-QY, equipped with an integrating sphere (Hamamatsu, Japan).

RL Intensity Measurements, X-Ray Photos Collection and Process: The RL spectra were measured by using a commercial miniature X-ray source, combined with an Edinburgh Fs5 fluorescence spectrophotometer (Edinburgh Instruments Ltd.). The X-ray source was a commercial miniature silver (Ag) target X-ray tube (AMPEK, Inc.), with a maximum tube current/power light output of 80 μA/4 W.

Crystal photographs and glass photographs under white light, UV light, and X-ray illumination were captured using a Canon EOS R5 Mark II camera equipped with a handheld UV lamp and a commercial X-ray tube (tungsten target, Moxtek). X-ray

imaging measurement: X-ray photos were taken by a Canon EOS R5 Mark II camera coupled with a commercial X-ray tube (tungsten target, Moxtek). All imaging tests are performed in a sealed lead box. By placing the samples between the X-ray source and the film with a distance of 3 cm. Images were obtained by taking photographs through the scintillator film.

Femtosecond transient absorption (fs-TA) measurements were conducted using a Yb:KGW amplifier (Pharos, Light Conversion) combined with a pump–probe system (Harpia, Light Conversion). The fundamental laser (1030 nm, ~190 fs, 89 kHz) from the amplifier was split into two beams. One beam was used to pump an optical parametric amplifier (Orpheus, Light Conversion) and a second harmonic generator (Lyra, Light Conversion) to generate tunable pump pulse. The other 1030 nm beam was focused into a sapphire crystal to produce white light continuum as probe pulse. The pump and probe pulses were focused and overlapped onto the sample. The transmitted probe pulse was dispersed by a monochromator (Andor Kymera 193i) and detected by a silicon-based array detector. The pump-probe delay can be up to ~8 ns controlled by a delay line.

4. Single-crystal Structure Analysis

Diffraction data were processed using the Crystal Clear 1.4.0 software, which includes symmetry-dependent data averaging and Lp-factor correction. The initial structural model of the crystals was determined using the SHELXT program.[1, 2] Non-hydrogen atoms were located from difference Fourier maps and refined anisotropically.[3, 4] The positions of hydrogen atoms were determined using a theoretical hydrogenation procedure. Although all hydrogen atoms were considered in the structural calculations, they were not included in the structure refinement. Main structural refinement data for the resulting crystals are presented in Table S1.

5. Quantum Chemical Calculations

Density functional theory (DFT) calculations were performed at the O3LYP/6-31G(d,p) level, as O3LYP has been reported to provide improved accuracy over B3LYP in describing supramolecular and weakly interacting organic systems, especially for π-conjugated and halogen-containing molecules.[5] The calculations were performed with the Gaussian 16 Revision A.03.[6] Visualization of the HOMO/LUMO orbitals was conducted using Multiwfn 3.8 and VMD.[7, 8]

6. Correlation Formulas and Analysis Methods

The X-ray attenuation efficiency (AE) was determined using the following Equation S1.

$$AE(\varepsilon, d) = \left(1 - e^{-c(\varepsilon)\rho d}\right) \times 100\%$$ Equation S1

where $c(\varepsilon)$ is the photon cross section function obtained from the XCOM database of National Institute of Standards and Technology (NIST), ε is the photon energy, ρ is the density of scintillator and d is the thickness.

The detection limit is calculated according to Equation S2.

$$LOD = \frac{3\sigma}{slope}$$ Equation S2

where σ is the instrumental average noise and slope is the slope of the fitted line.

The light yield of the sample was determined using a reference method, with a BGO crystal serving as the standard reference.[9] The thickness of the sample was measured using the glassy scintillators with the measured thickness of the standard sample (d = 0.19 cm). The light yield of the BGO crystal was set at 8,000 photons MeV^{-1}. Both the test sample and the standard sample were exposed to the same X-ray source. Emitted photons from the sample were collected using an Edinburgh FS5 fluorescence spectrophotometer, and the light yield was calculated using the Equation S3:

$$LY_{sample} = LY_{BGO} \times \frac{\int I_{sample}(\lambda) d\lambda \times AE_{BGO}}{\int I_{BGO}(\lambda) d\lambda \times AE_{sample}}$$ Equation S3

$I(\lambda)$ is the radioluminescence spectrum of the scintillator measured by the Edinburgh FS5 fluorescence spectrophotometer.

Crystalline powders and glassy samples were analyzed using the FLS980 spectrometer with excitation, and the fluorescence decay times were calculated using Equation S4:

$$I(t) = I_0 + A_1 \exp\left(-t/\tau_1\right) + A_2 \exp\left(-t/\tau_2\right)$$ Equation S4

where $I(t)$, I_0 and A_1 are the photoluminescence intensity at time, background and amplitude, respectively. And the average lifetime (τ_{ave})[9] is calculated as Equation S5.

$$\tau_{ave} = (A_1\tau_1^2 + A_2\tau_1^2)/(A_1\tau_1 + A_2\tau_2)$$ Equation S5

The non-radiative transition rate is calculated by Equation S6.

$$k(nr) = \frac{1-\Phi_f}{\tau_1}$$ Equation S6

where Φ_f is the PLQY and τ is the lifetime.

The spatial resolution of X-ray imaging was determined using Modulation Transfer Function (MTF) measurements. The line-pair card captured in the X-ray image was analyzed using ImageJ software to obtain a curve of pixel intensity versus pixel position. MTF[10] was then calculated using Equation S7.

$$MTF = \frac{I_{max}-I_{min}}{I_{max}+I_{min}}$$ Equation S7

where I_{min} and I_{max} are the minimum and maximum values of the function used to quantify the contrast, respectively.

To further refine the resolution measurement, the slanted edge method was applied. Sharp-edge X-ray imaging was conducted on a standard tungsten sheet with an approximate thickness of 0.5 mm. The MTF was computed using ImageJ software, first, the edge spread function (ESF) was derived from the edge image, then the line spread function (LSF) was obtained by differentiating the ESF[11]. Finally, the MTF was defined as the Fourier transform of the LSF, as described in Equation S8.

$$MTF(v) = F[LSF(x)] = F\left[\frac{dLSF(x)}{dx}\right]$$ Equation S8

where v was the spatial frequency and x is the position of pixels.

7. Related Figures and Tables

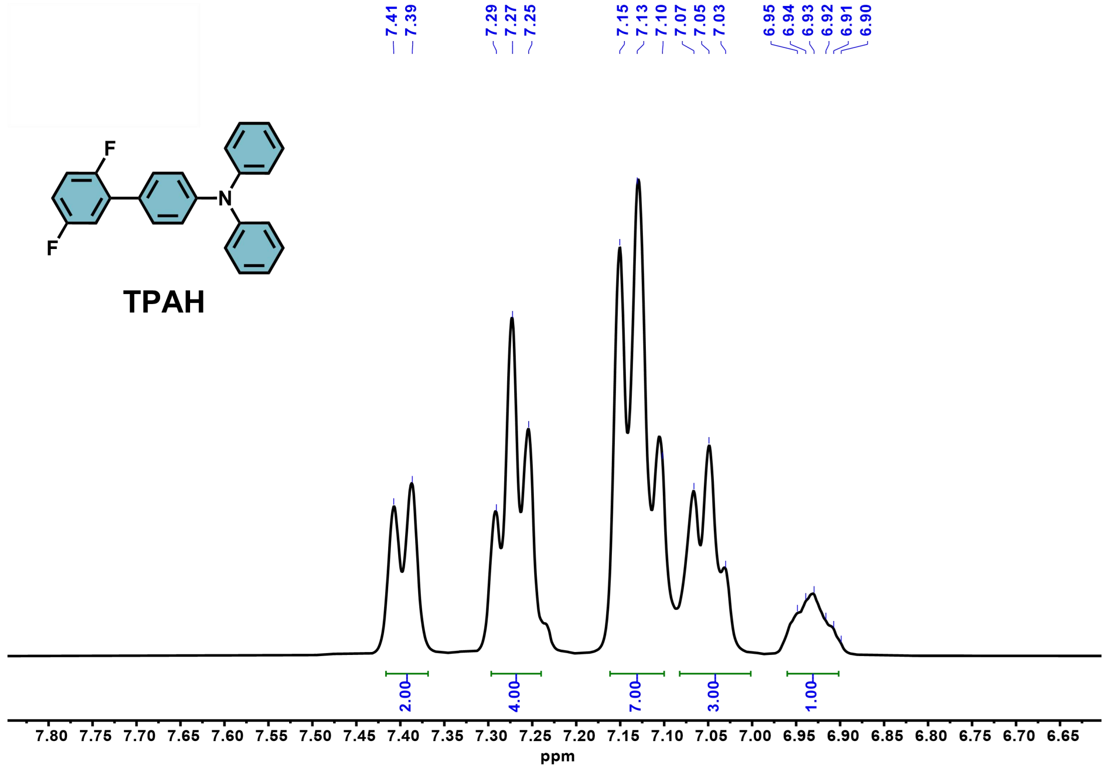

Figure S6. ^{1}H NMR spectrum of TPAH in $CDCl_3$ (25 °C, 400 MHz).

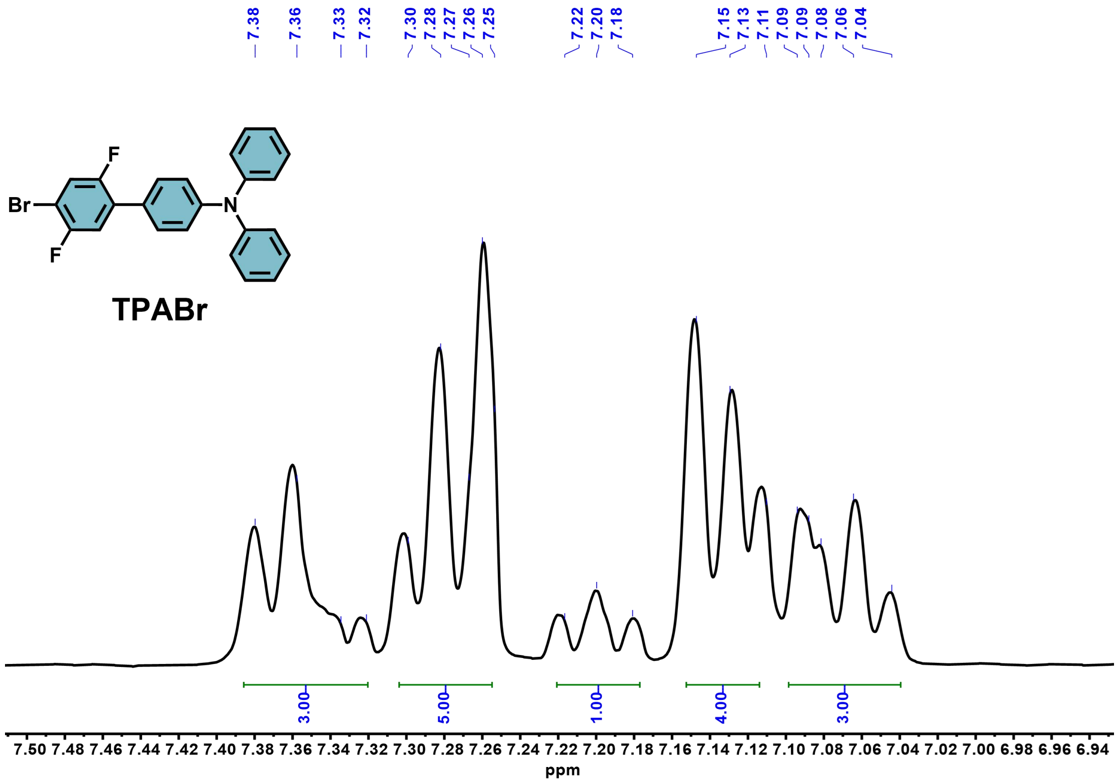

Figure S7. ^{1}H NMR spectrum of TPABr in $CDCl_3$ (25 °C, 400 MHz).

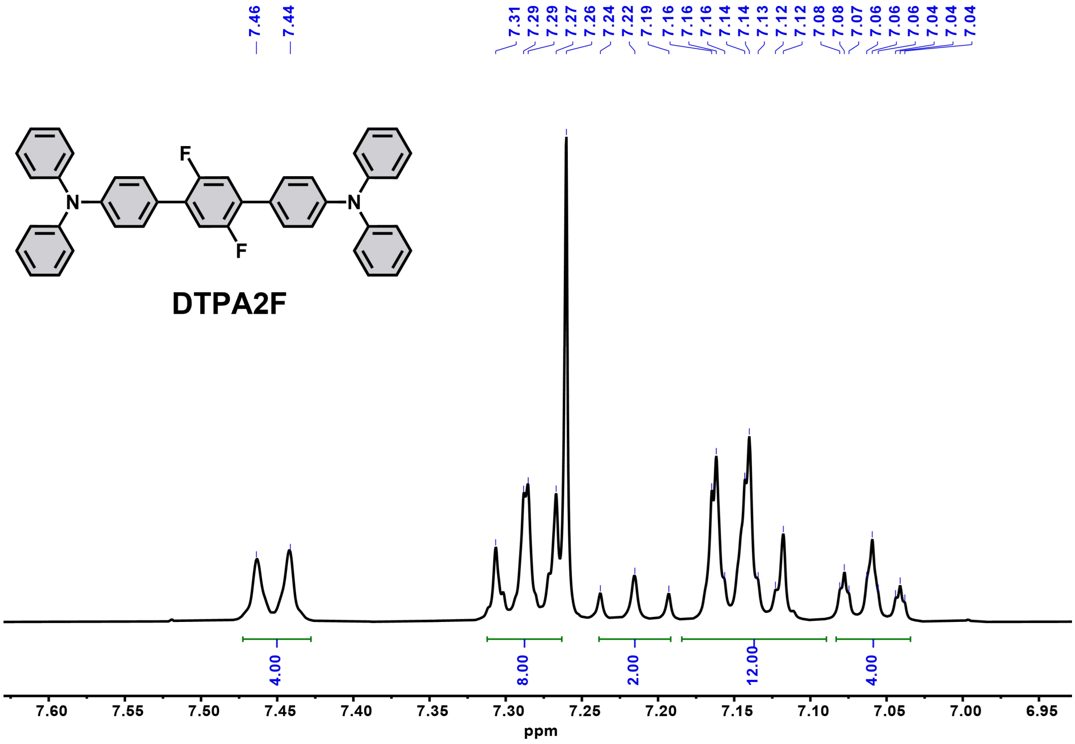

Figure S8. ^{1}H NMR spectrum of DTPA2F in $CDCl_3$ (25 °C, 400 MHz).

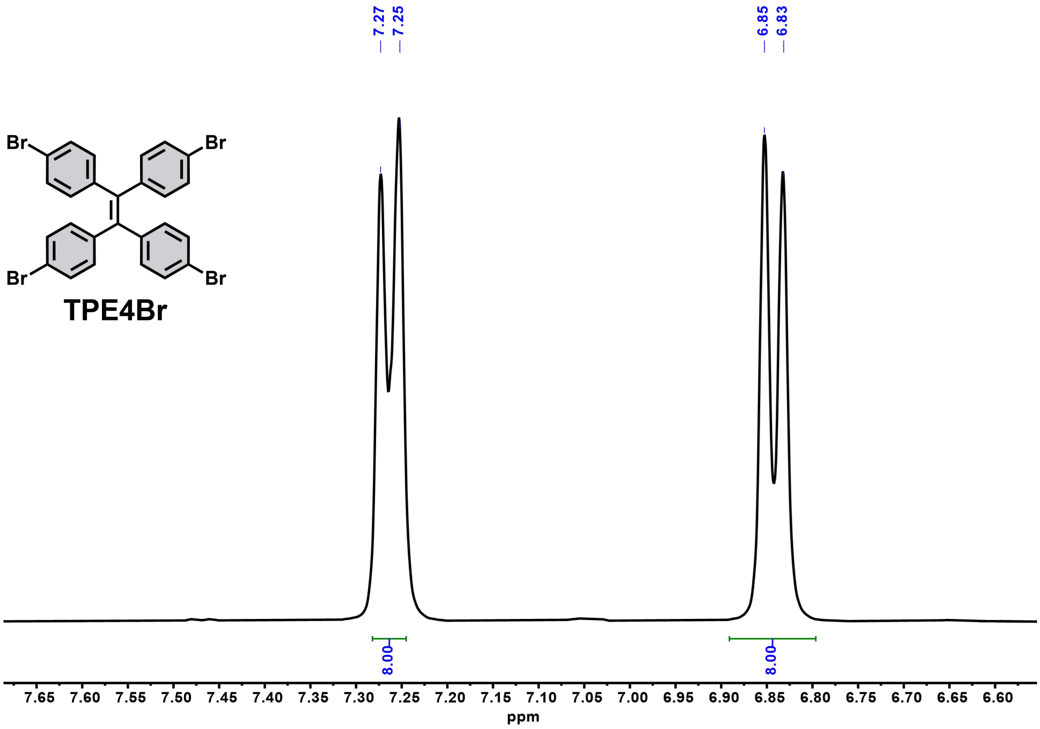

Figure S9. ^{1}H NMR spectrum of TPE4Br in $CDCl_3$ (25 °C, 400 MHz).

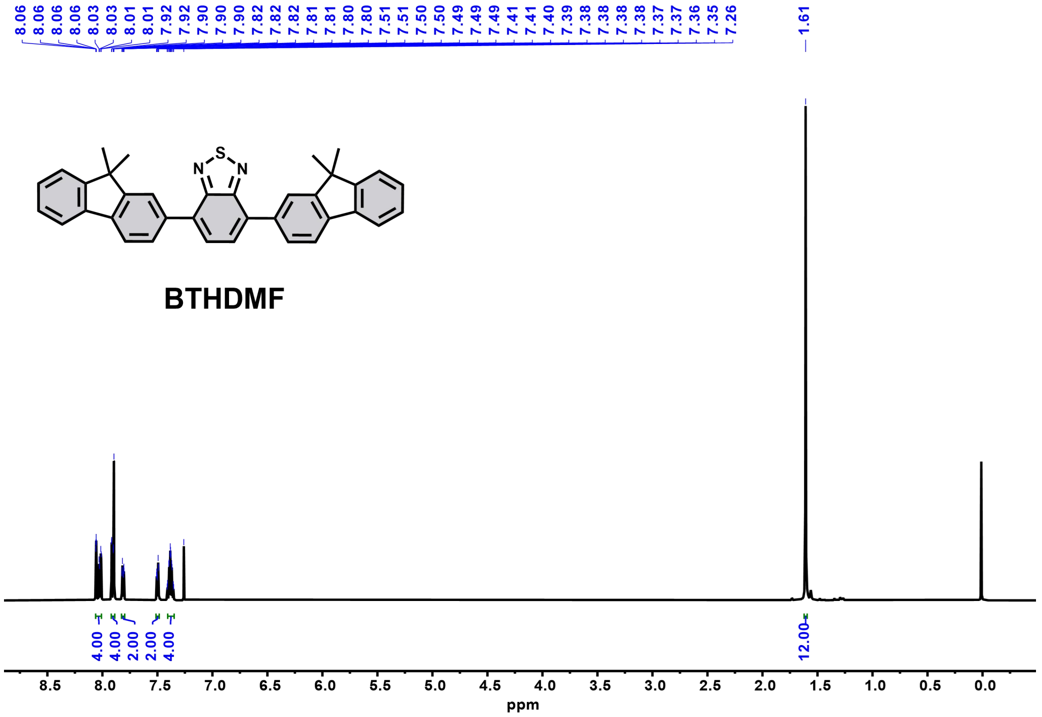

Figure S10. ^1H NMR spectrum of BTHDMF in $CDCl_3$ (25 °C, 500 MHz).

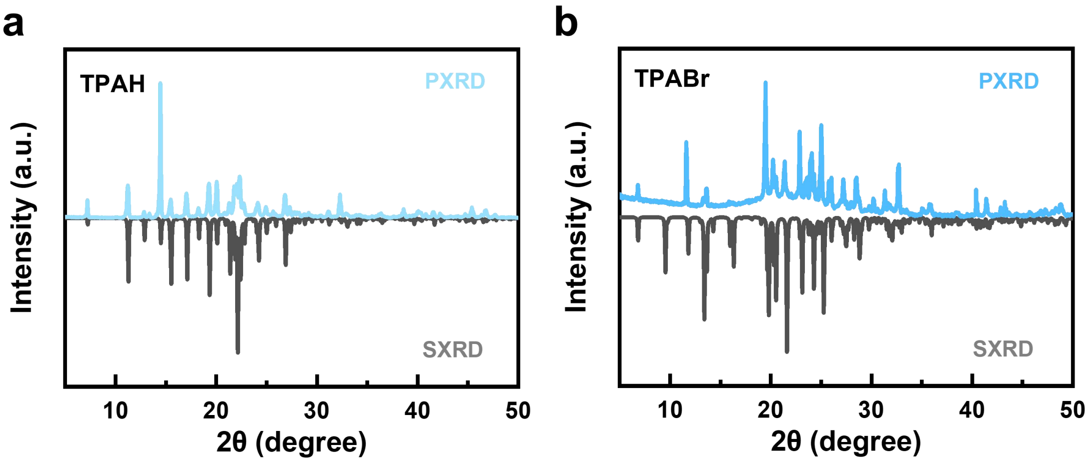

Figure S11. (a–b) XRD patterns of TPAH and TPABr crystalline powders compared with their simulated counterparts.

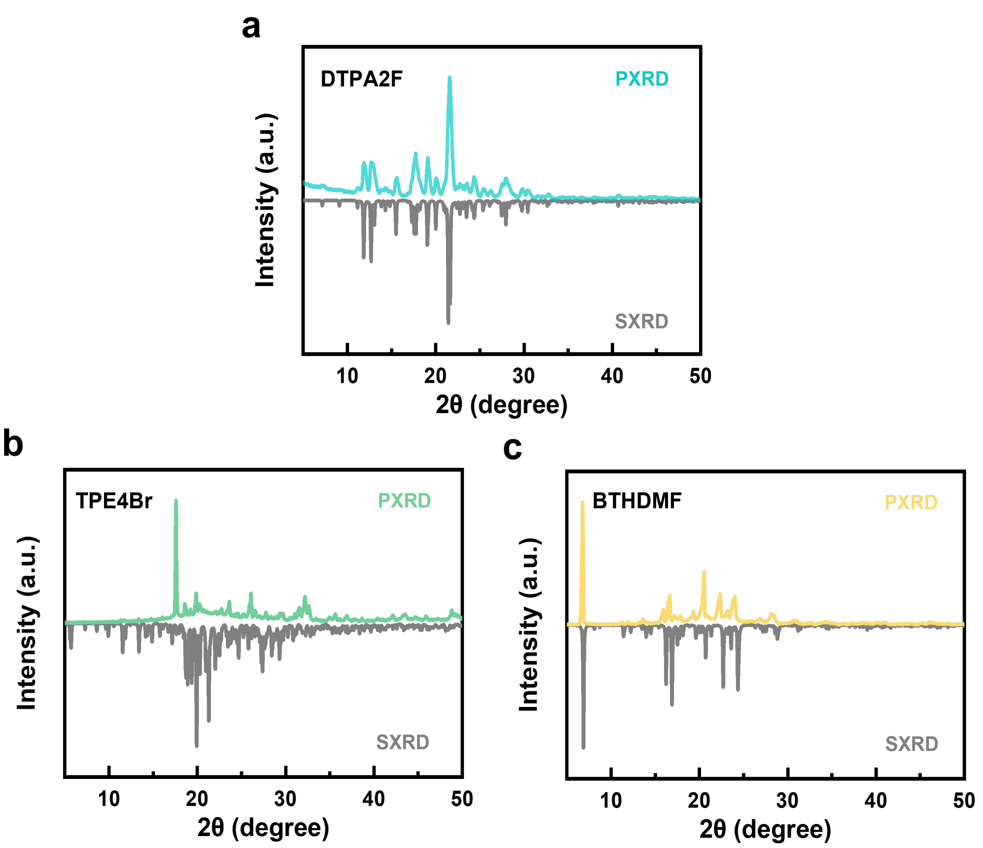

Figure S12. (a–c) XRD patterns of DTPA2F, TPE4Br and BTHDMF crystalline powders compared with their simulated counterparts.

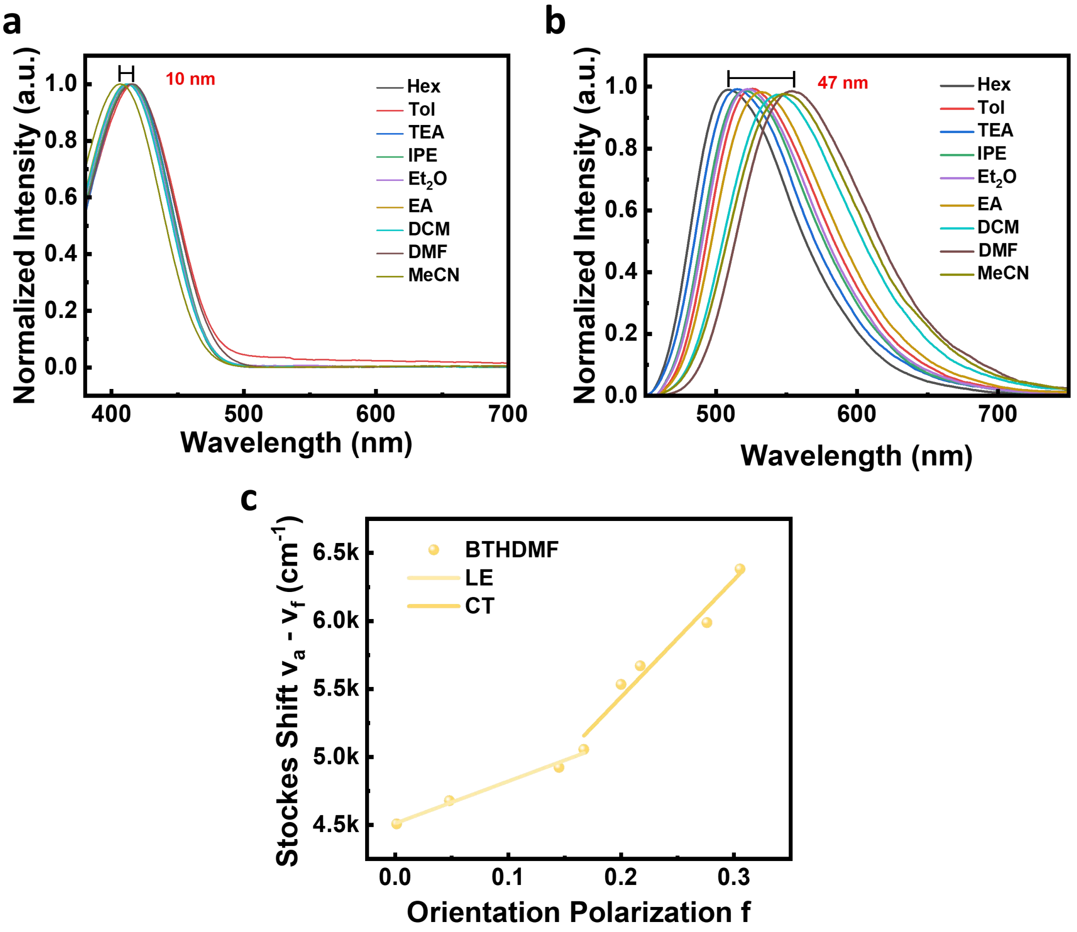

Figure S13. (a–b) UV–vis absorption spectra and normalized photoluminescence (PL) spectra of BTHDMF in solvents with varying polarity. **c** Lippert–Mataga plot of Stokes shift versus orientation polarizability (f).

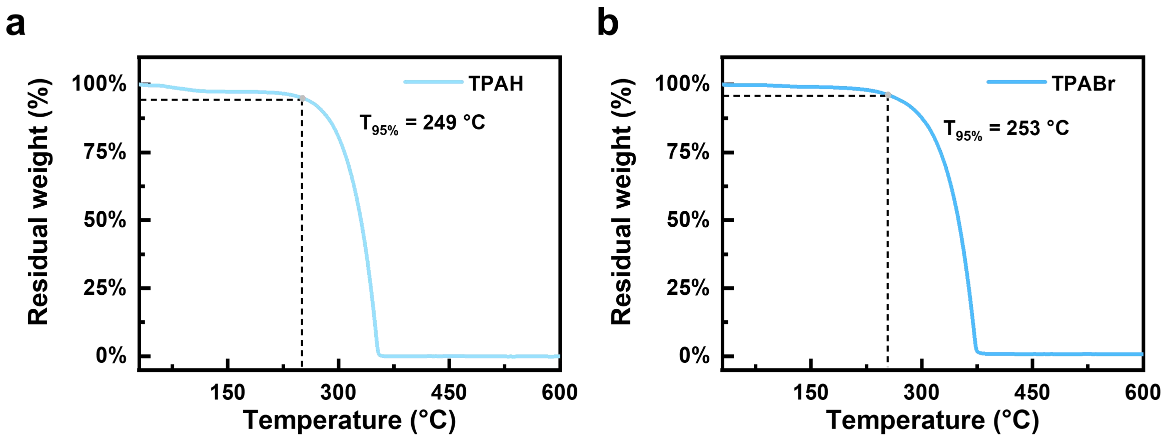

Figure S14. (a–b) Thermal properties of TPAH and TPABr crystalline powders.

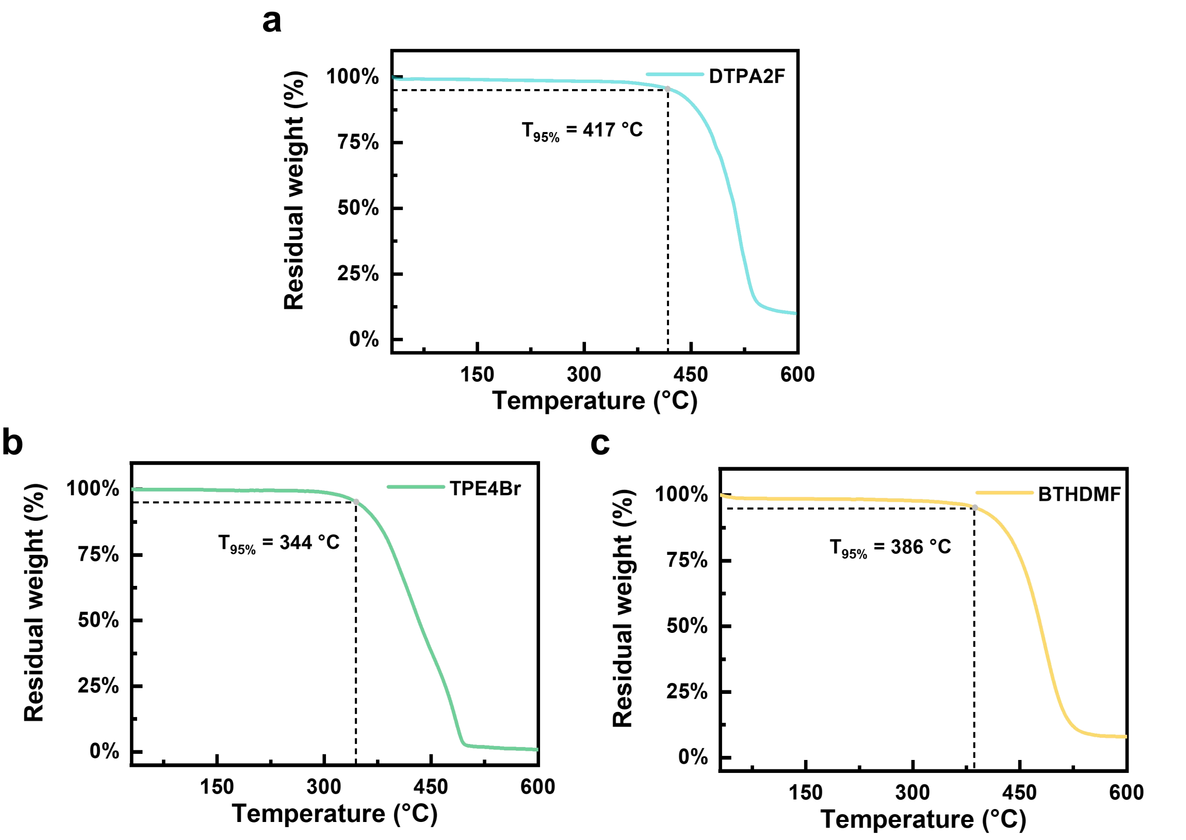

Figure S15. (a–c) Thermal properties of DTPA2F, TPE4Br and BTHDMF crystalline powders.

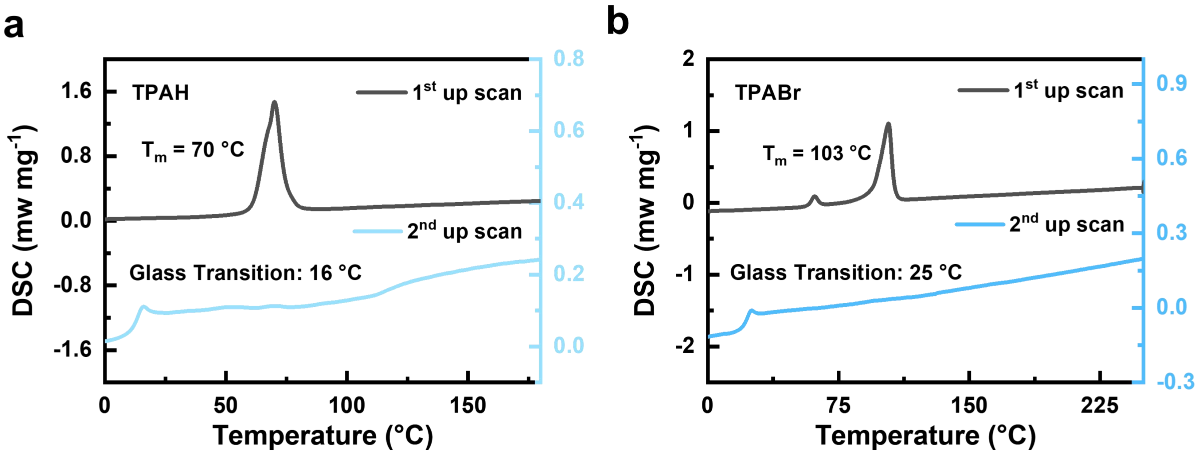

Figure S16. (a–b) DSC curves plot of TPAH and TPABr crystalline powders.

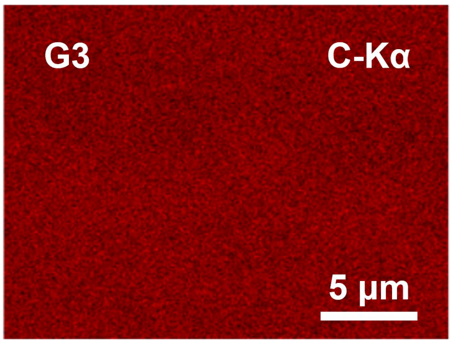

Figure S17. EDS elemental mapping of carbon (C-Kα) for G3 with the scale bar of 5 μm.

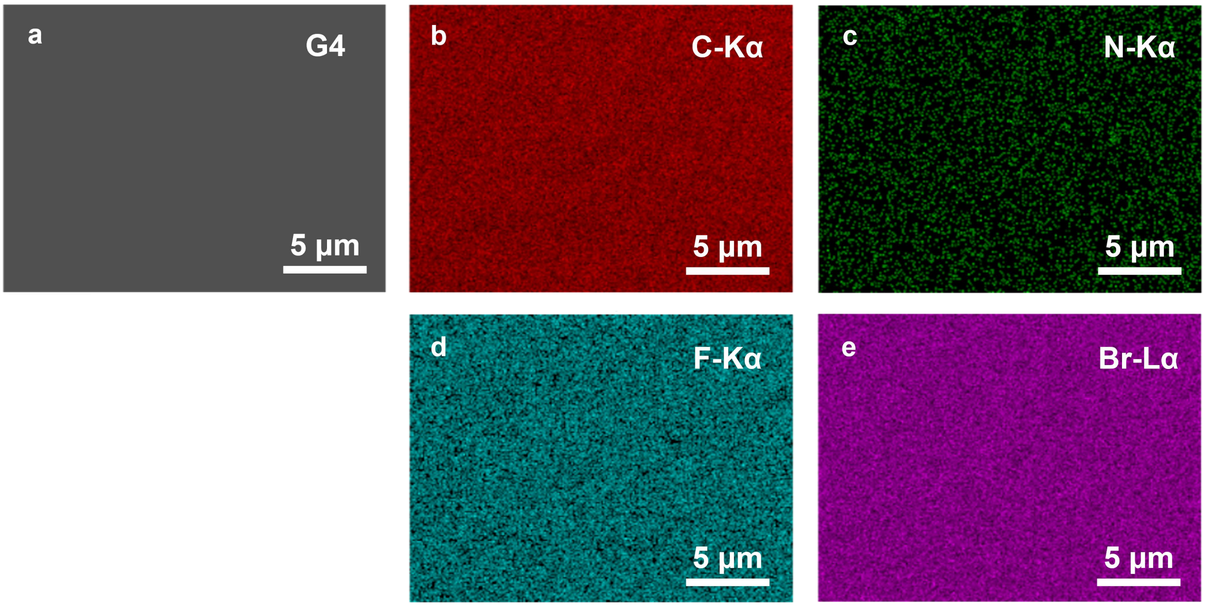

Figure S18. (a–e) SEM and EDS elemental mapping of G4 with the scale bar of 5 µm.

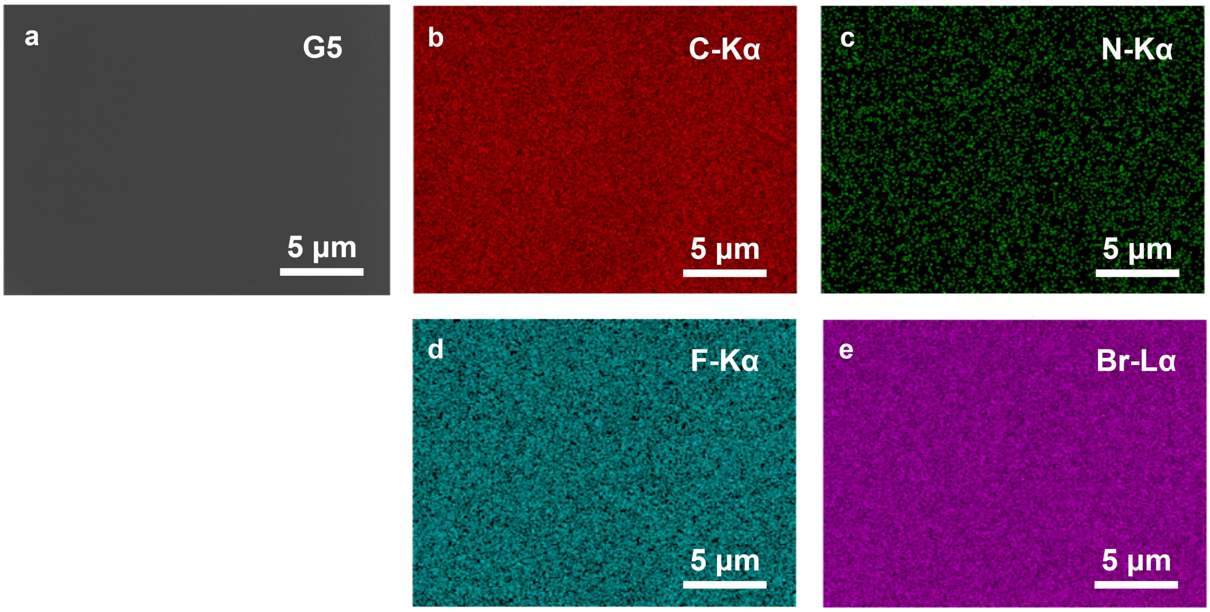

Figure S19. (a–e) SEM and EDS elemental mapping of G5 with the scale bar of 5 µm.

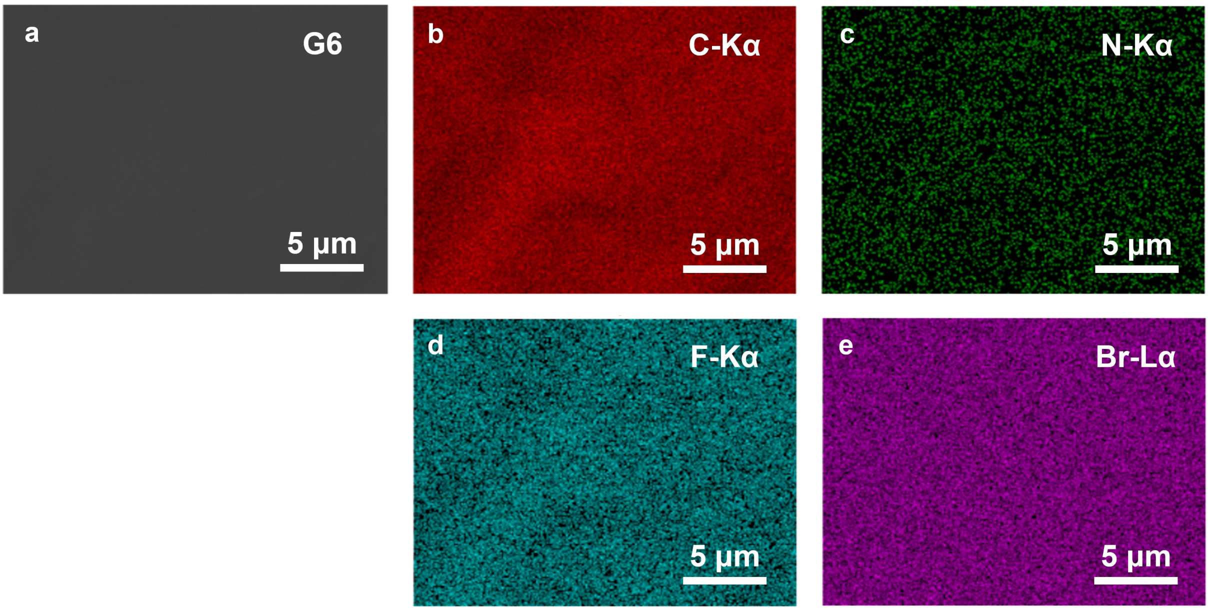

Figure S20. (a–e) SEM and EDS elemental mapping of G6 with the scale bar of 5 μm.

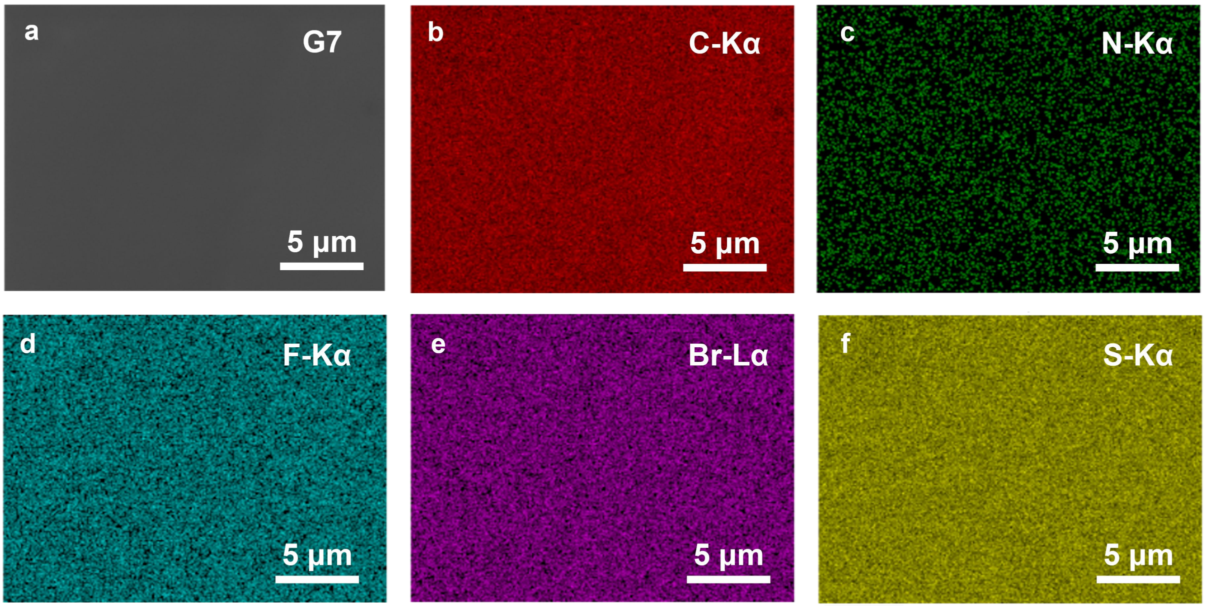

Figure S21. (a–f) SEM and EDS elemental mapping of G7 with the scale bar of 5 μm.

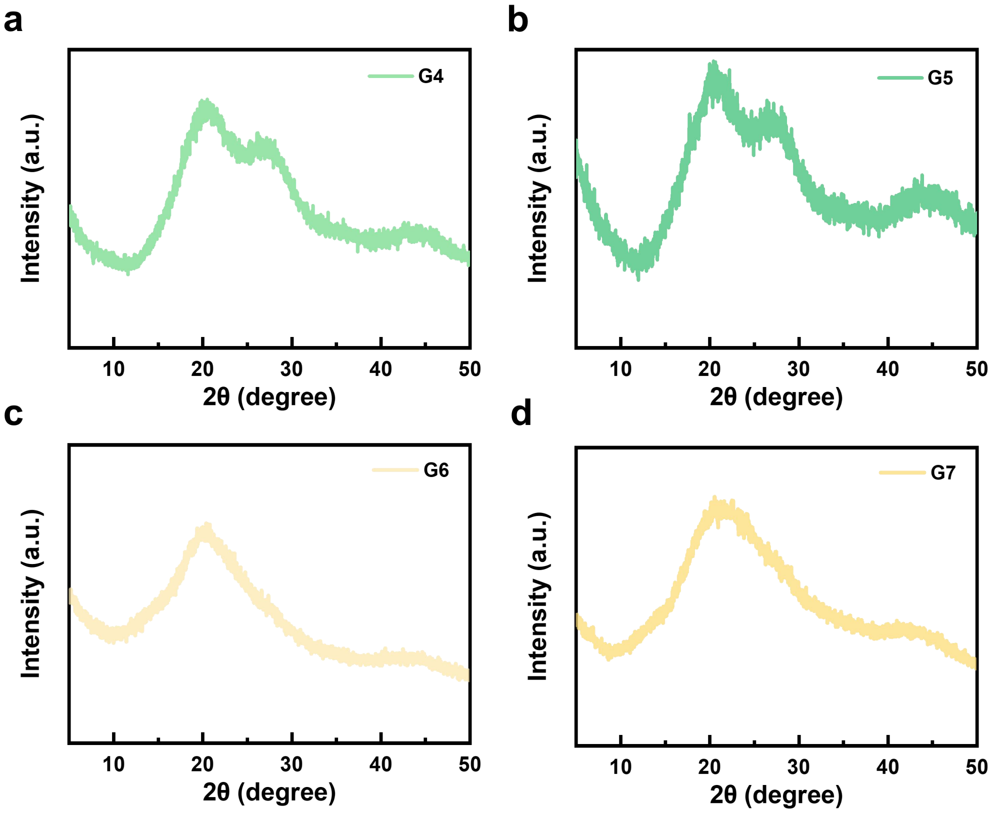

Figure S22. (a–d) XRD pattern of G4, G5, G6, and G7.

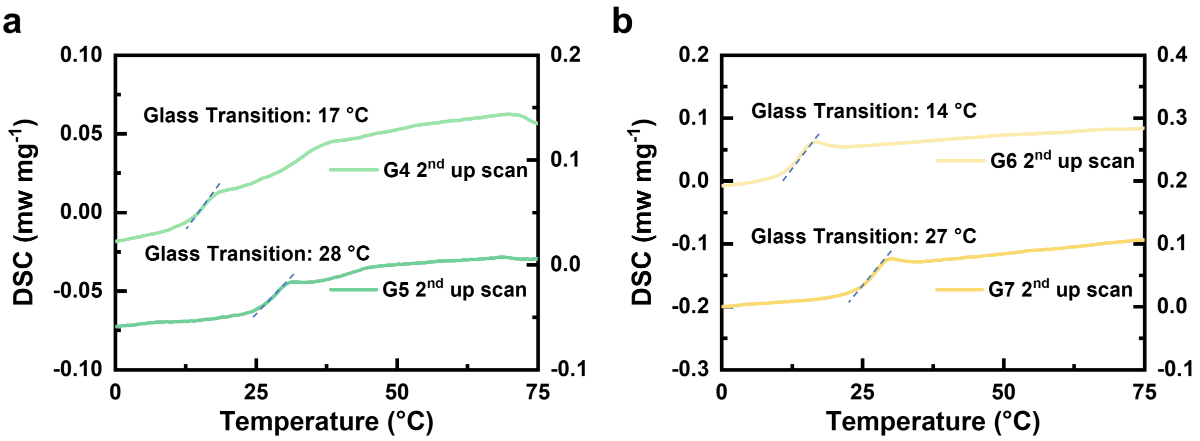

Figure S23 (a–b) DSC curves of glassy scintillators G4–G7 showing their respective glass transition temperatures.

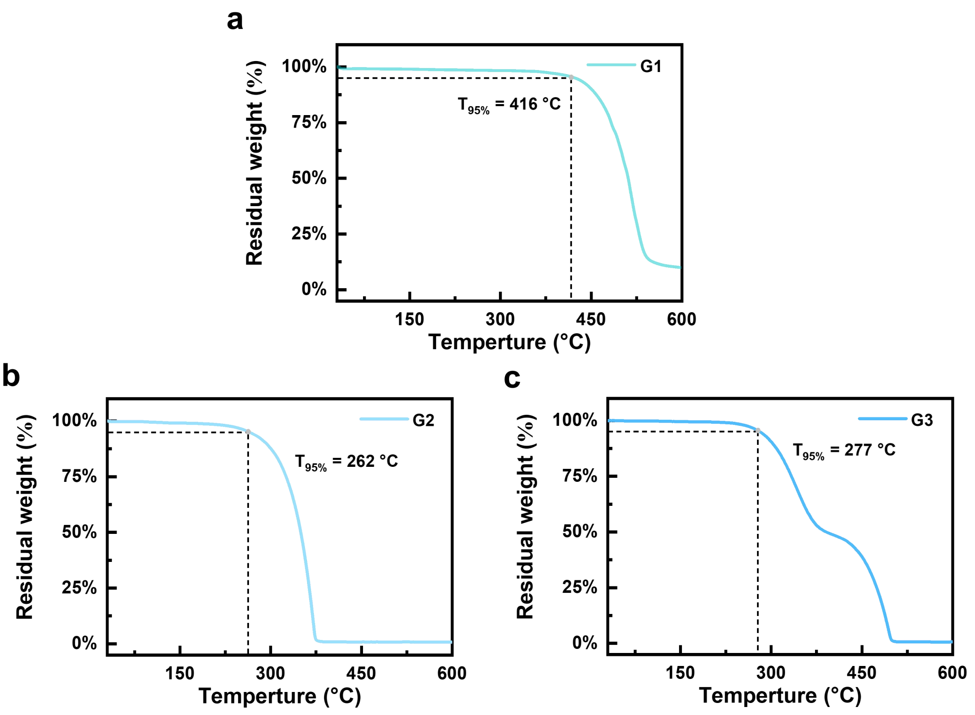

Figure S24. (a–c) Thermal properties of G1, G2, and G3.

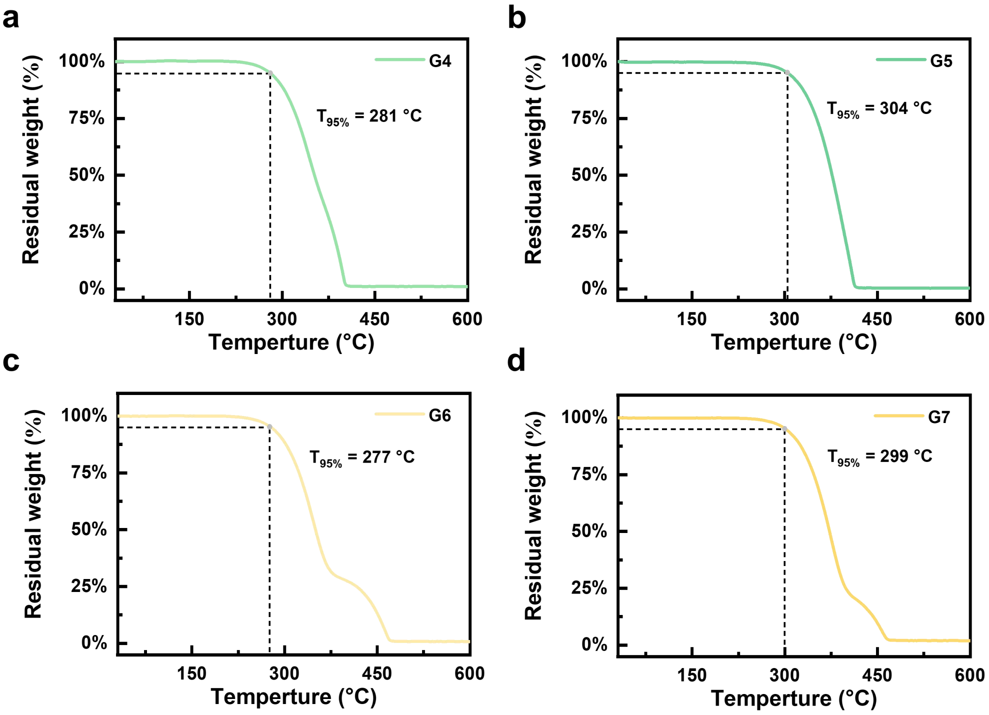

Figure S25. (a–d) Thermal properties of G4, G5, G6 and G7.

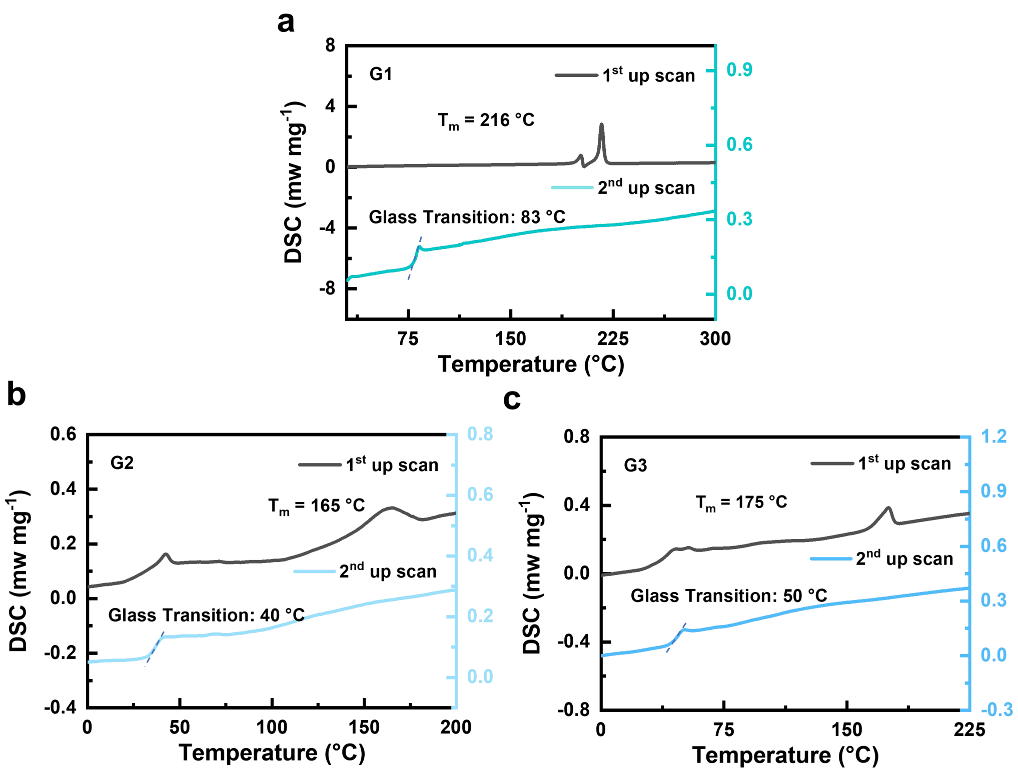

Figure S26. (a–c) DSC curves plot of G1, G2 and G3.

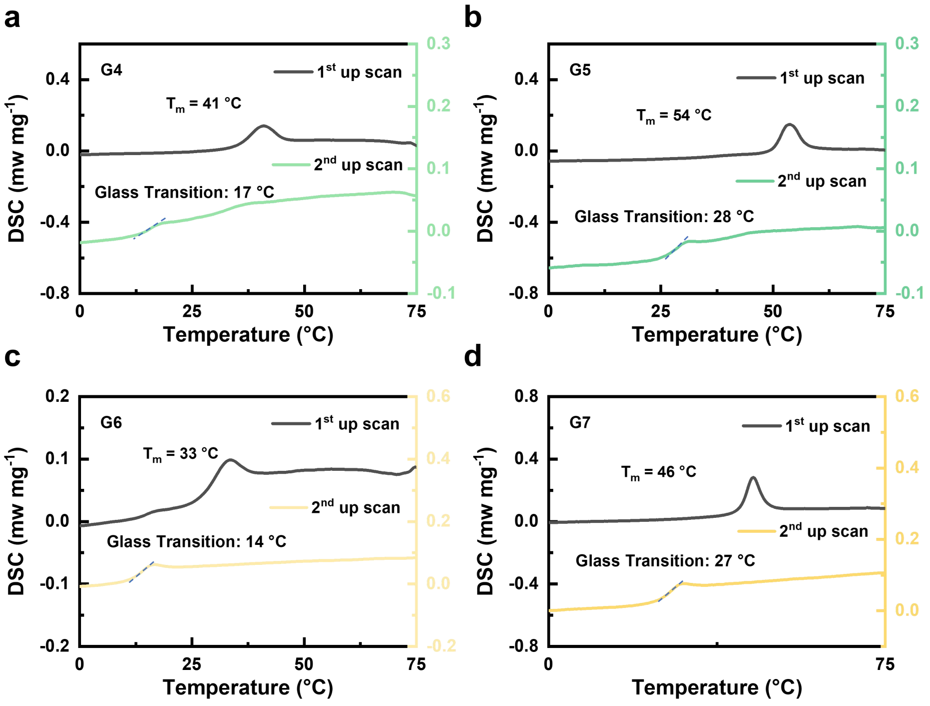

Figure S27. (a–d) DSC curves plot of G4, G5, G6, and G7.

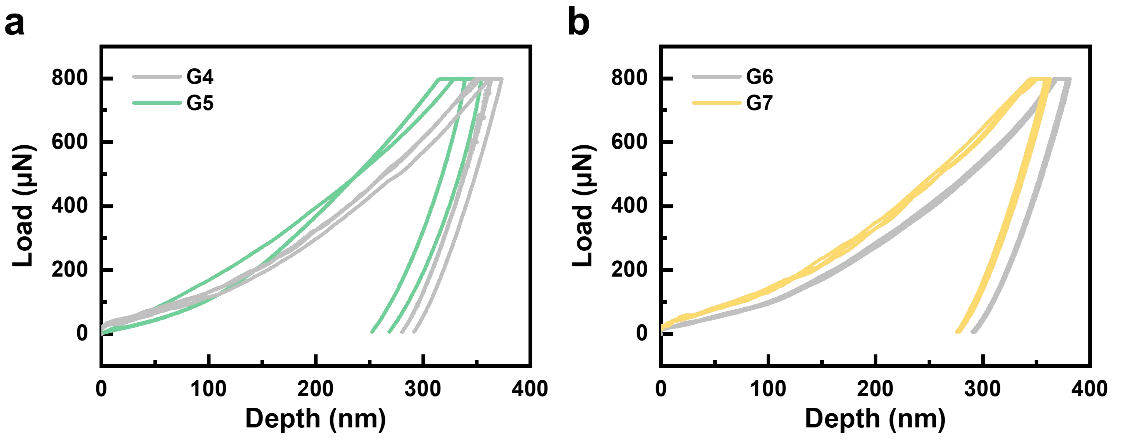

Figure S28. (a–b) Load–displacement curves of G4–G7 obtained from nanoindentation measurements.

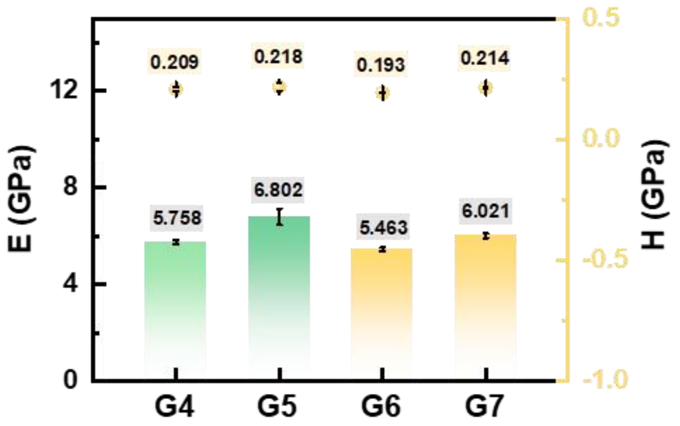

Figure S29. The charts of Young's modulus (*E*) and hardness (*H*) values for G4–G7.

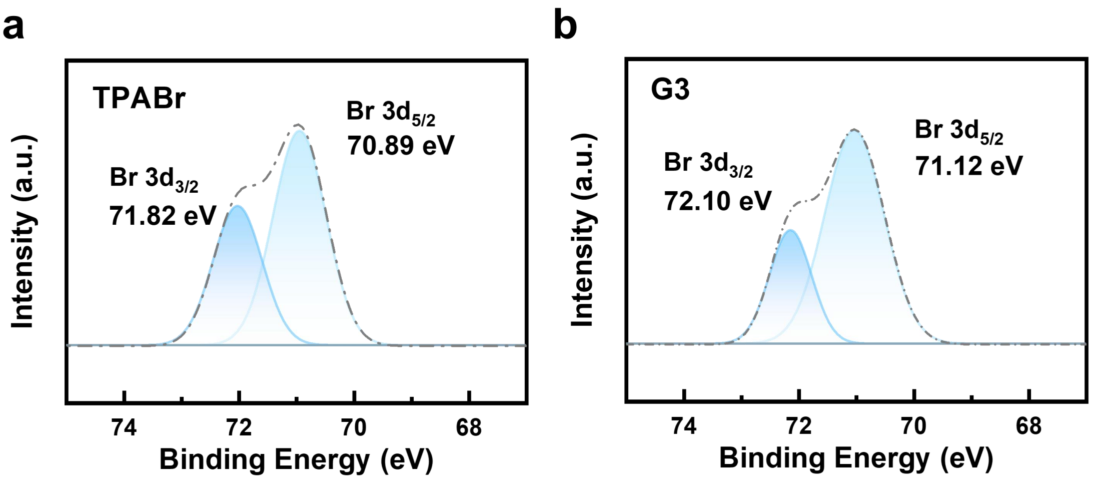

Figure S30. (a–b) The core-level XPS patterns of Br 3d of TPABr and G3.

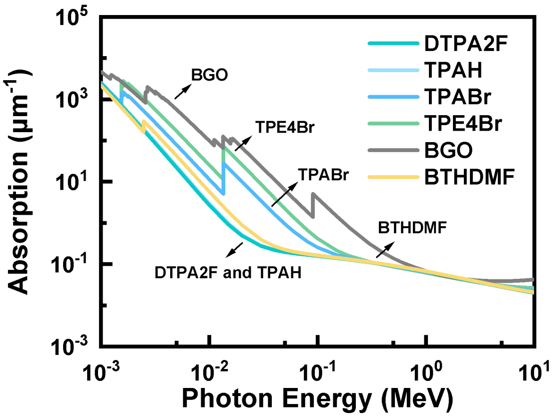

Figure S31. The X-ray absorption coefficients of TPAH, TPABr, DTPA2F, TPE4Br, BTHDMF and BGO.

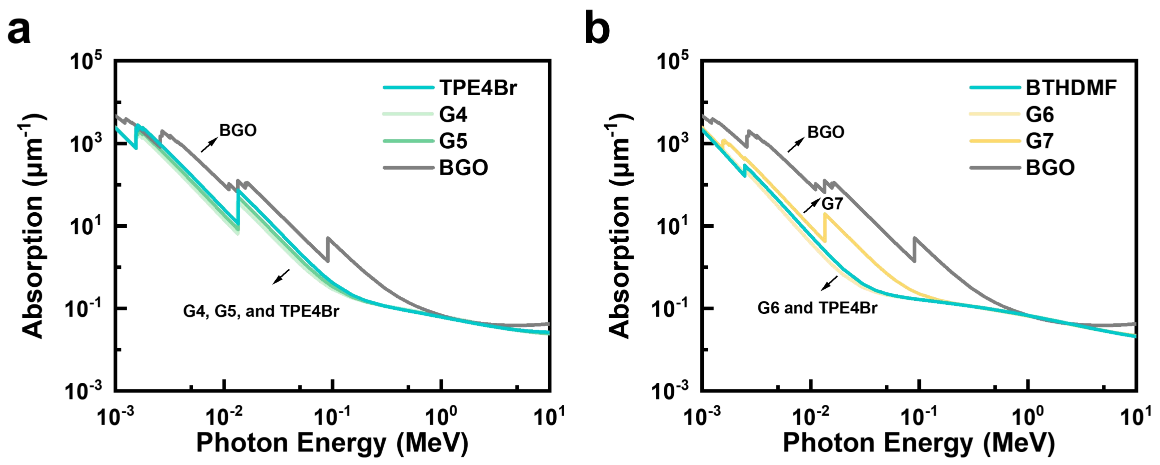

Figure S32. (a–b) X-ray absorption coefficients of TPE4Br, BTHDMF, BGO, and G4–G7 as a function of photon energy.

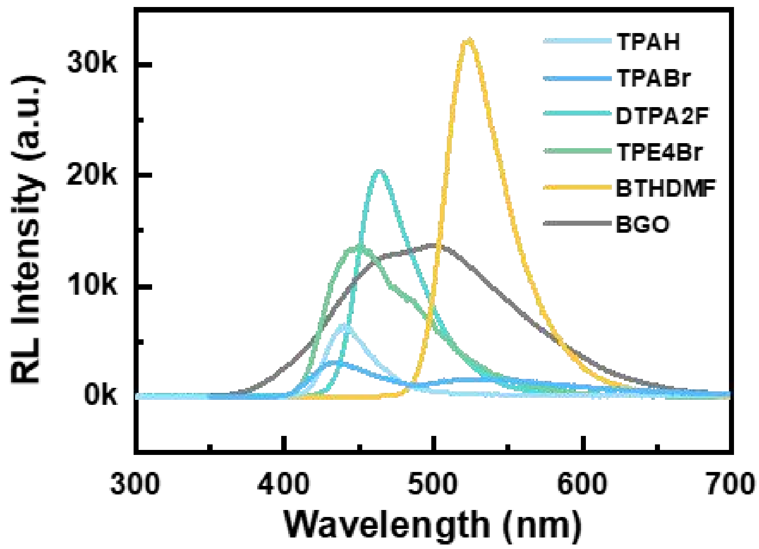

Figure S33. The RL spectra of TPAH TPABr, DTPA2F, TPE4Br, BTHDMF and BGO at the X-ray dose of 278 μGy s^{-1}.

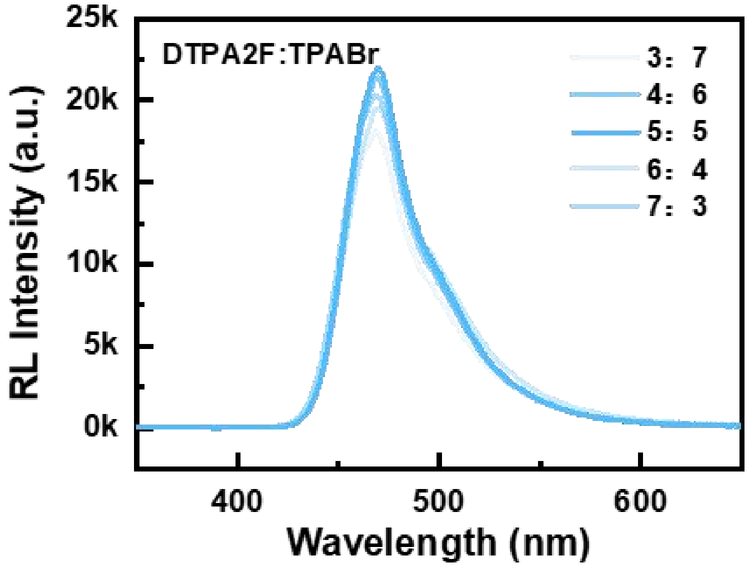

Figure S34. RL spectra of glassy samples with varying DTPA2F: TPABr ratios under X-ray irradiation at the X-ray dose of 278 μGy s^{-1}.

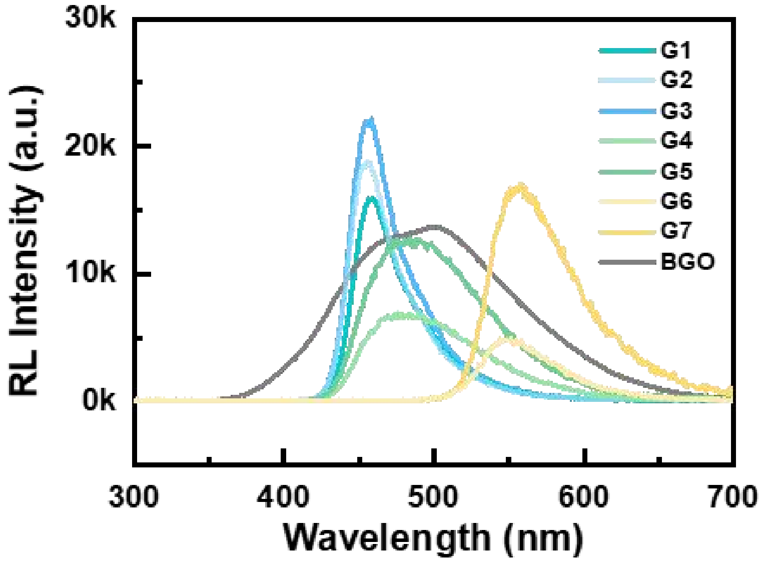

Figure S35. The RL spectra of G1–G7 and BGO at the X-ray dose of 278 μGy s^{-1}.

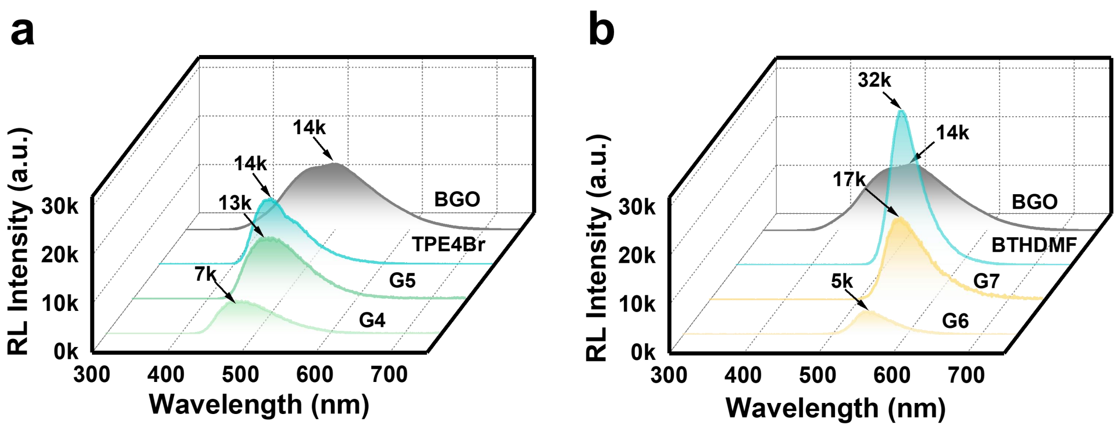

Figure S36. (a–b) RL spectra of G4–G7, TPE4Br, and BTHDMF under an X-ray dose of 278 μGy s^{-1}, with BGO as reference.

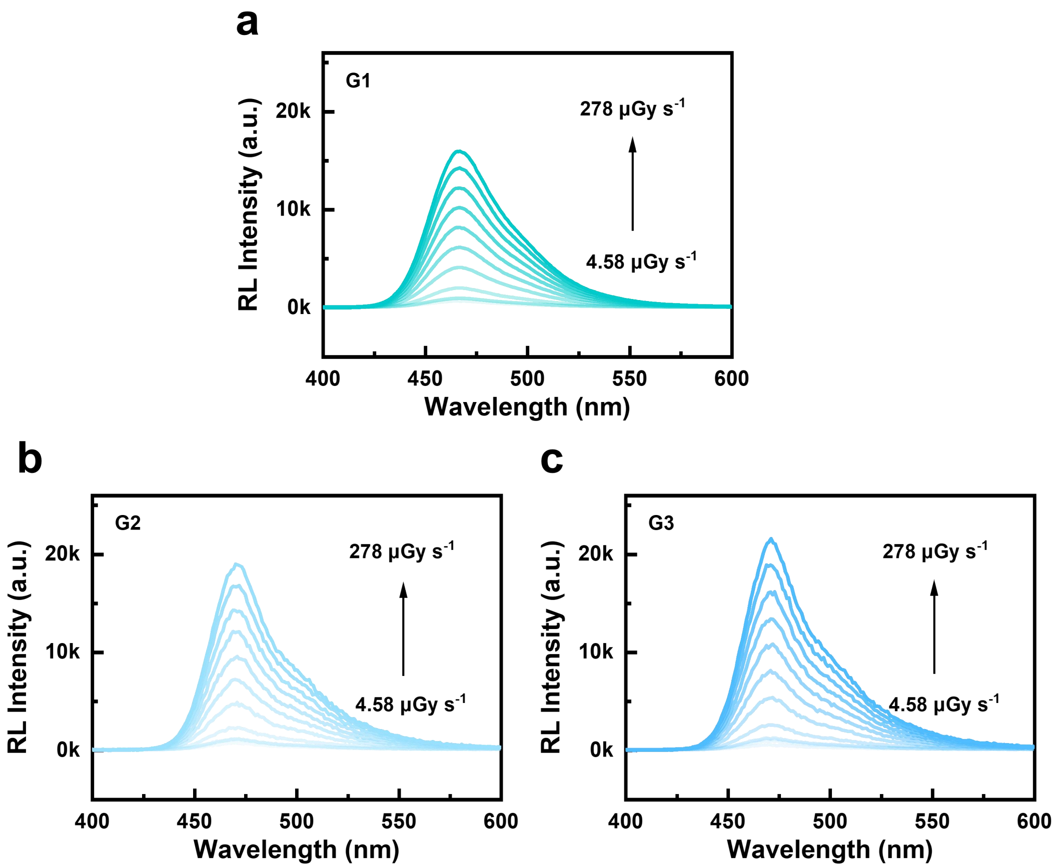

Figure S37. (a–c) The RL intensity of G1–G3 in the range of 4.58 μGy s^{-1} to 278 μGy s^{-1}.

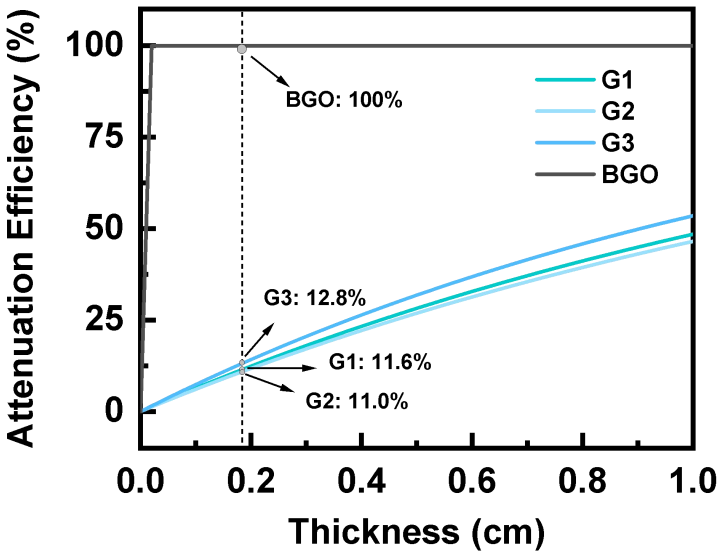

Figure S38. X-ray attenuation efficiencies as functions of material thickness for G1, G2, G3 and BGO.

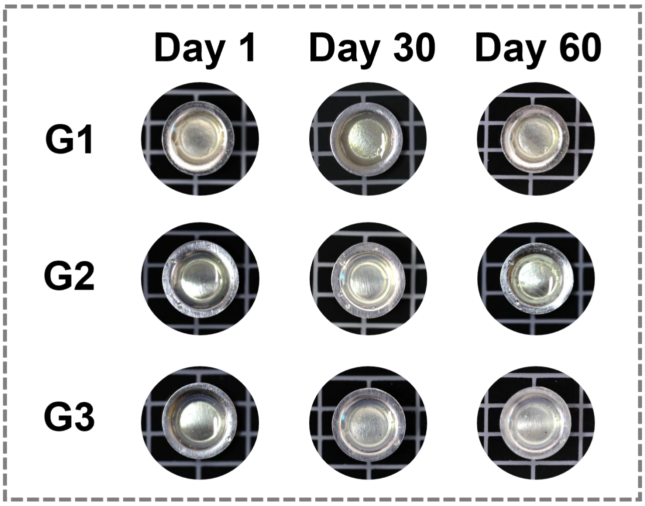

Figure S39. Optical photographs of representative glass samples (G1–G3) under UV irradiation after storage for 1, 30, and 60 days at 35 °C and 80% relative humidity, demonstrating their air stability.

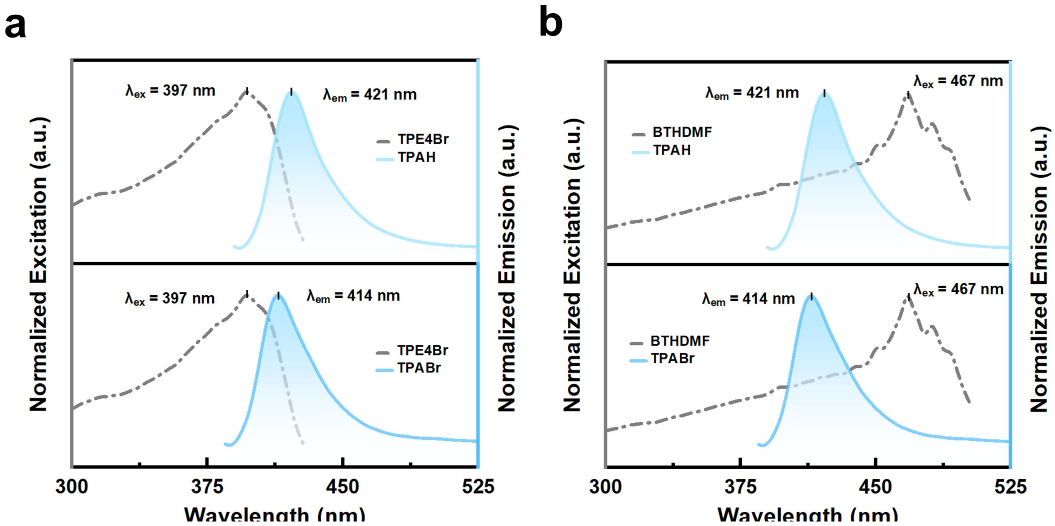

Figure S40. (a) Normalized excitation spectrum of TPE4Br and emission spectra of TPAH and TPABr. (b) Normalized excitation spectrum of BTHDMF and emission spectra of TPAH and TPABr.

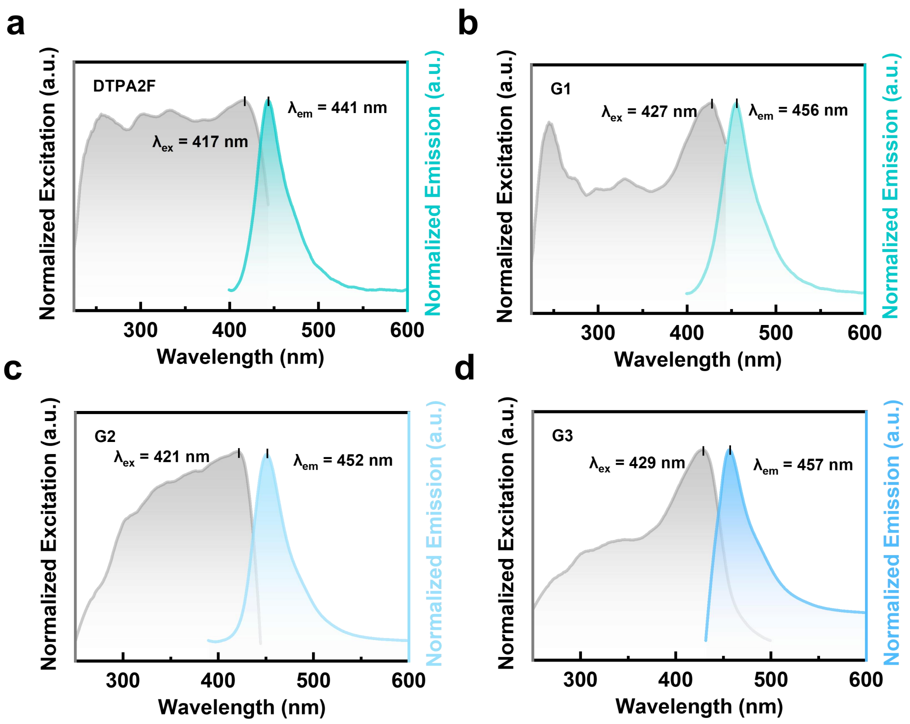

Figure S41. (a–d) Normalized excitation and emission spectra of DTPA2F, G1, G2, and G3.

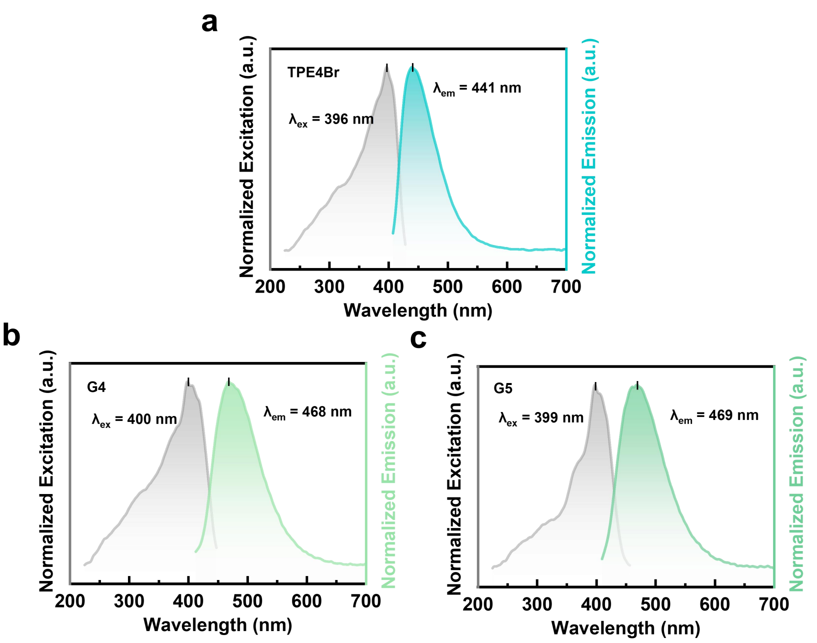

Figure S42. (a–c) Normalized excitation and emission spectra of TPE4Br, G4, and G5.

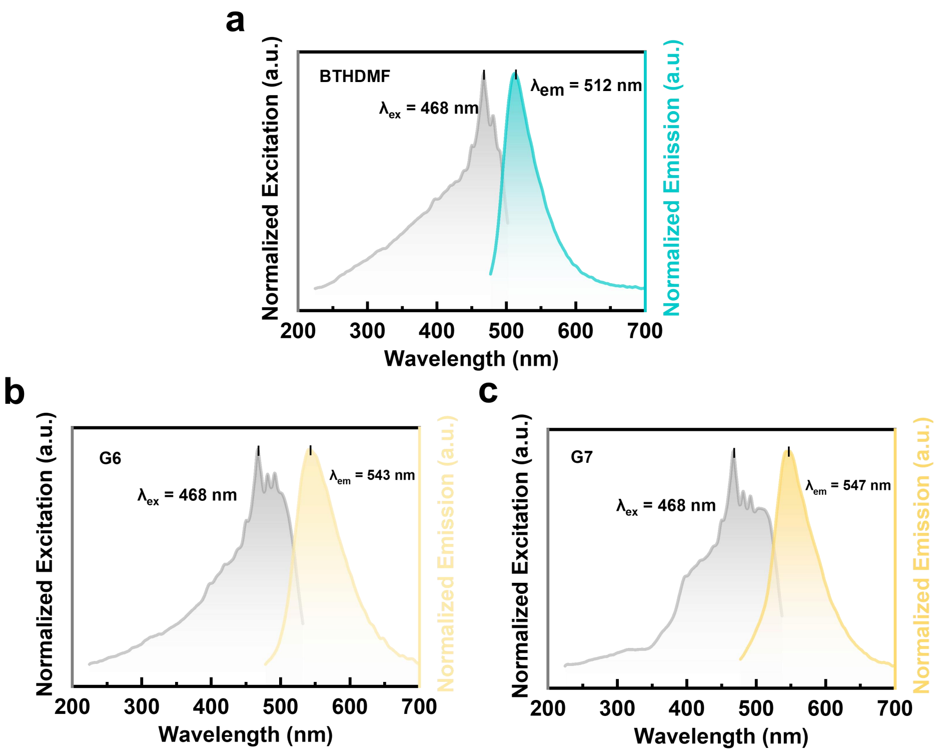

Figure S43. (a–c) Normalized excitation and emission spectra of BTHDMF, G6, and G7.

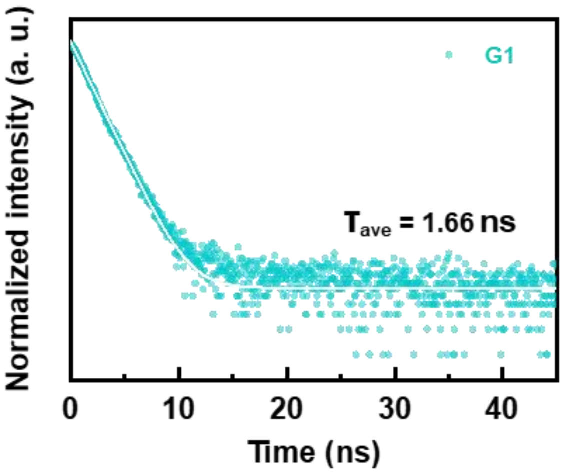

Figure S44. The lifetime decay curves of G1.

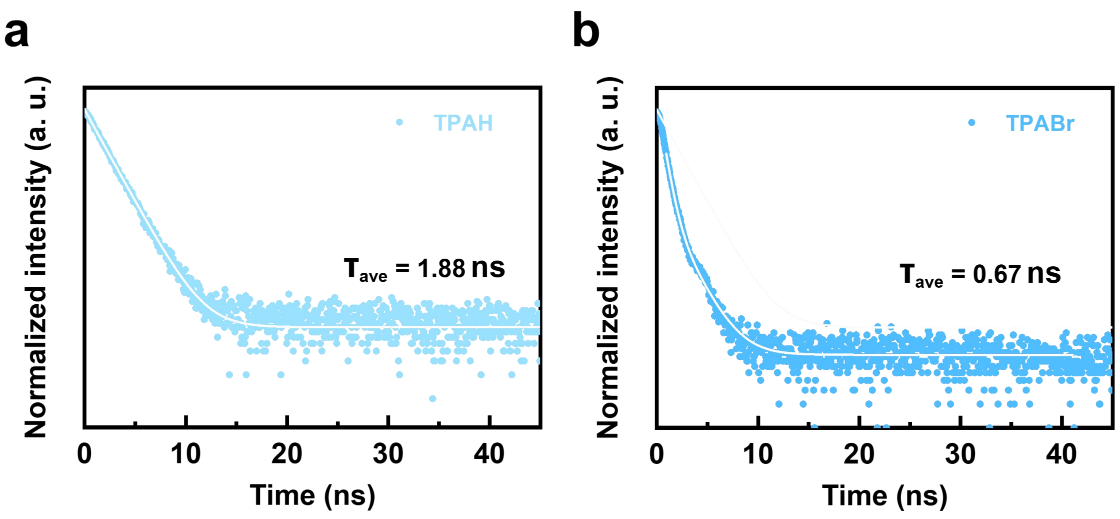

Figure S45. (a–b) The lifetime decay curves of TPAH and TPABr.

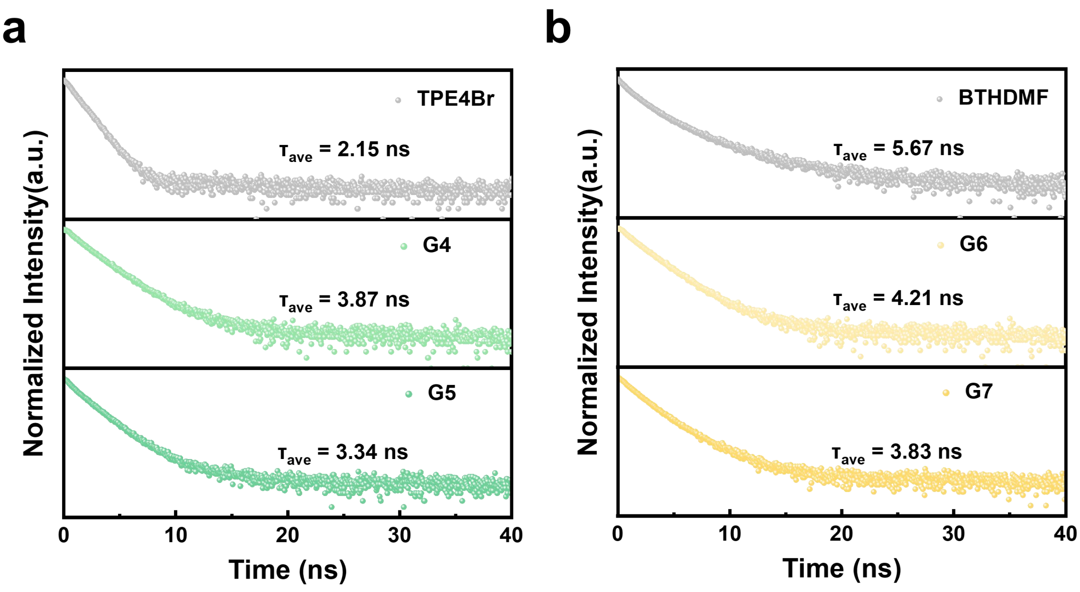

Figure S46. (a) Lifetime decay curves of TPE4Br, G4, and G5. (b) Lifetime decay curves of BTHDMF, G6, and G7.

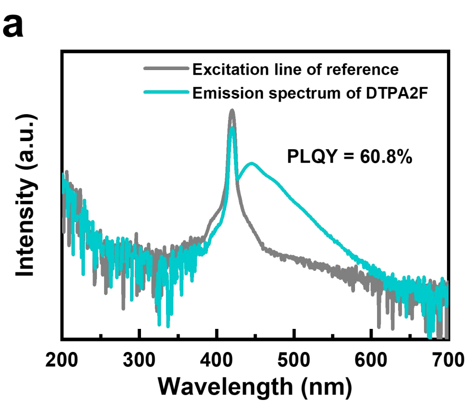

Figure S47. (a–b) The PLQY spectrum (reference line and emission spectrum) of DTPA2F and G1.

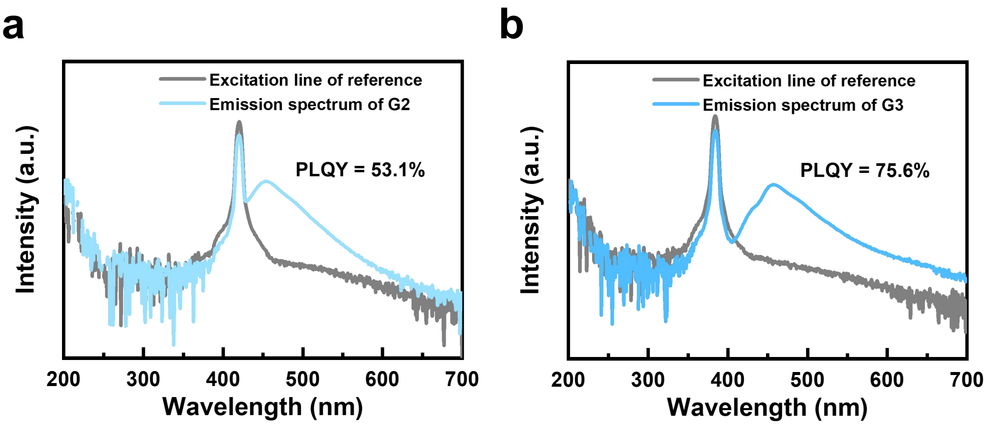

Figure S48. (a–b) The PLQY spectrum (reference line and emission spectrum) of G2 and G3.

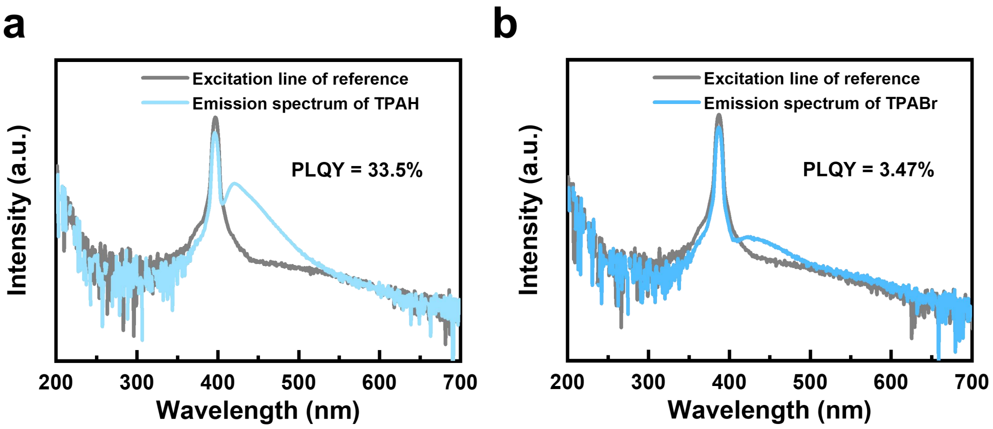

Figure S49. (a–b) The PLQY spectrum (reference line and emission spectrum) of TPAH and TPABr.

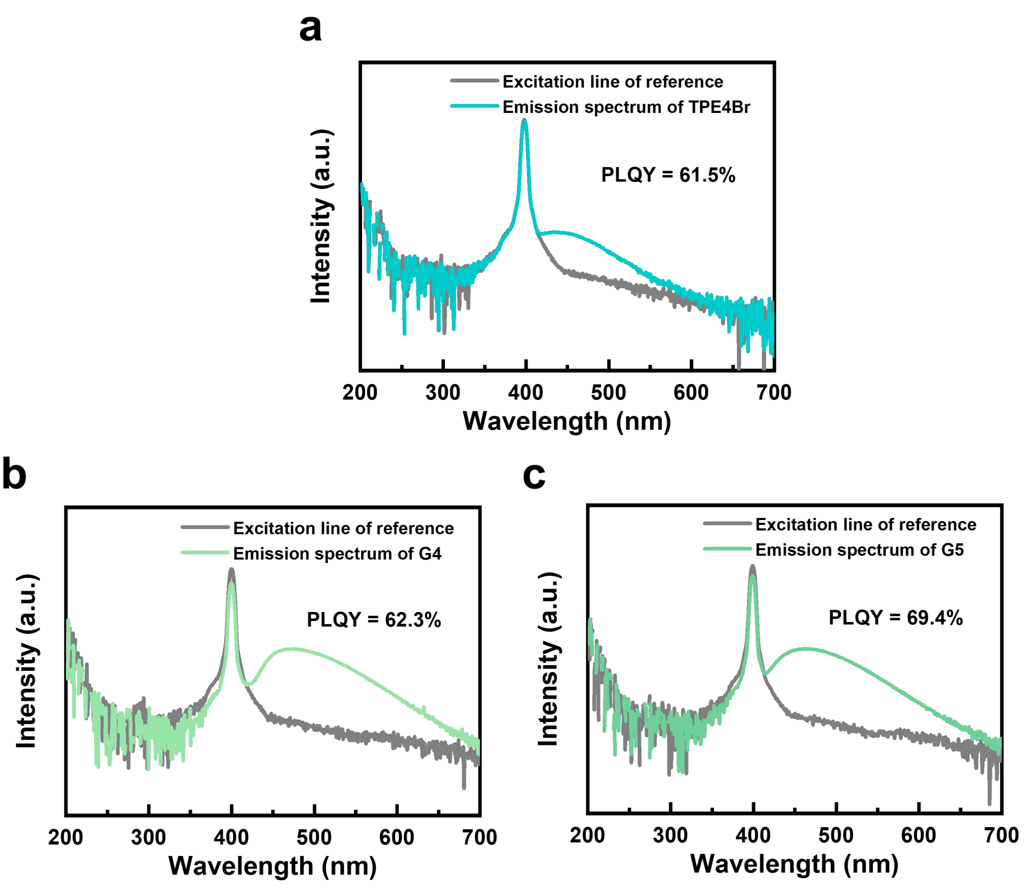

Figure S50. (a–c) The PLQY spectrum (reference line and emission spectrum) of TPE4Br, G4, and G5.

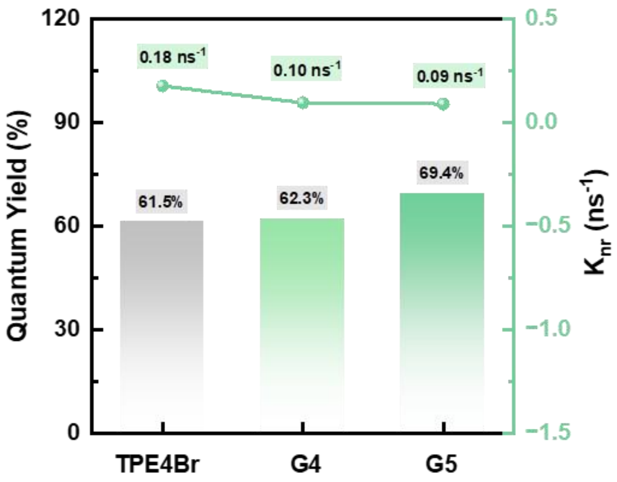

Figure S51. PLQY and K_{nr} of TPE4Br, G4, and G5.

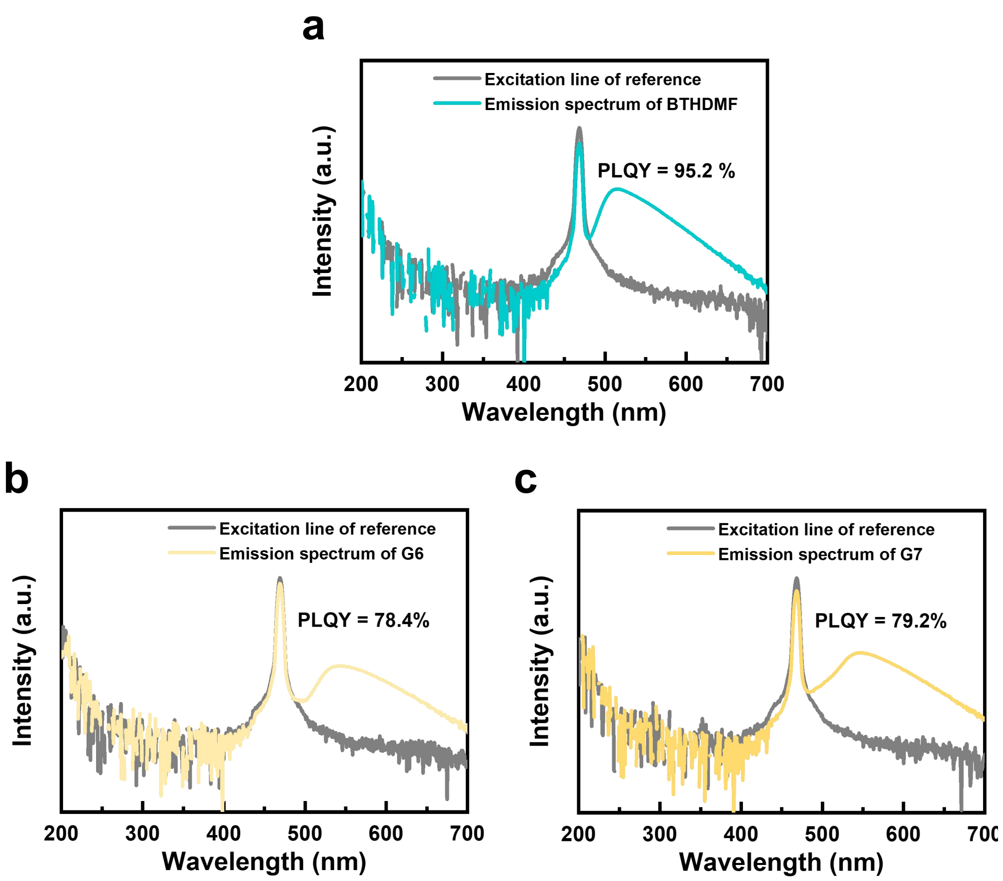

Figure S52. (a–c) The PLQY spectrum (reference line and emission spectrum) of BTHDNF, G6, and G7.

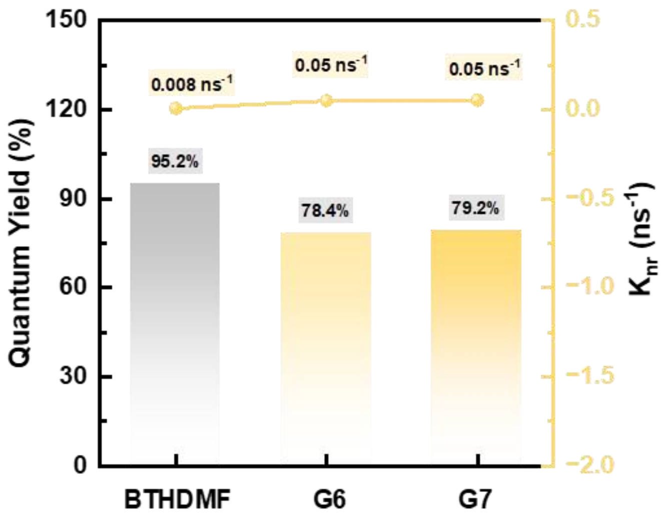

Figure S53. The PLQY spectra of BTHDMF, G6, and G7.

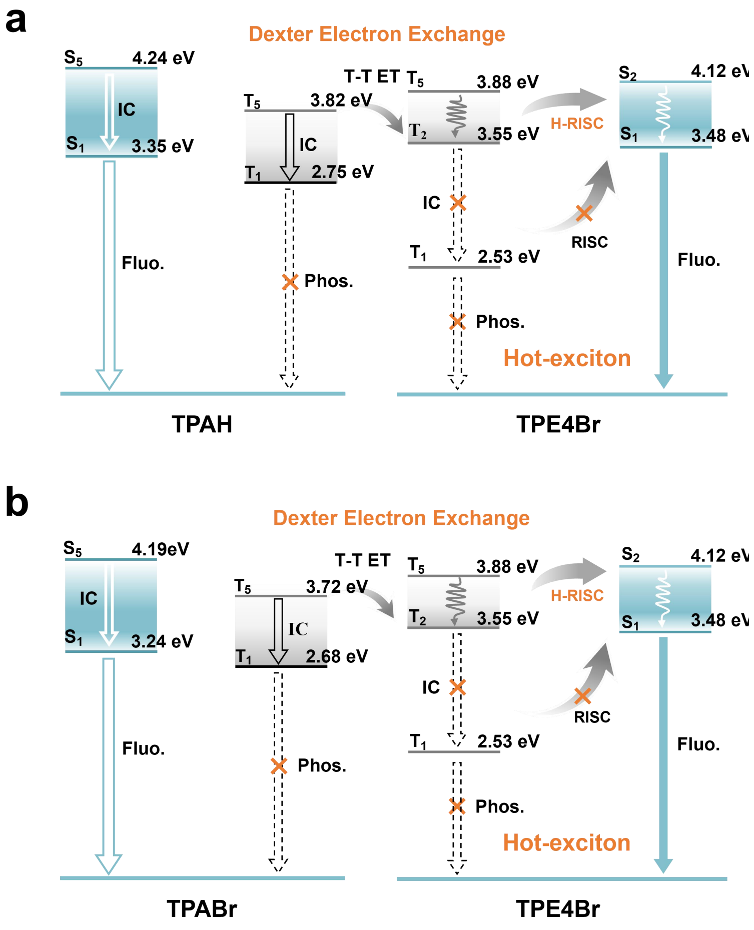

Figure S54. (a–b) Proposed photophysical pathways and energy transfer mechanisms in the G4 (TPAH–TPE4Br) and G5 (TPABr–TPE4Br) co-melting systems, respectively, including Dexter electron exchange, reverse intersystem crossing (RISC), and hybrid RISC (H-RISC) processes.

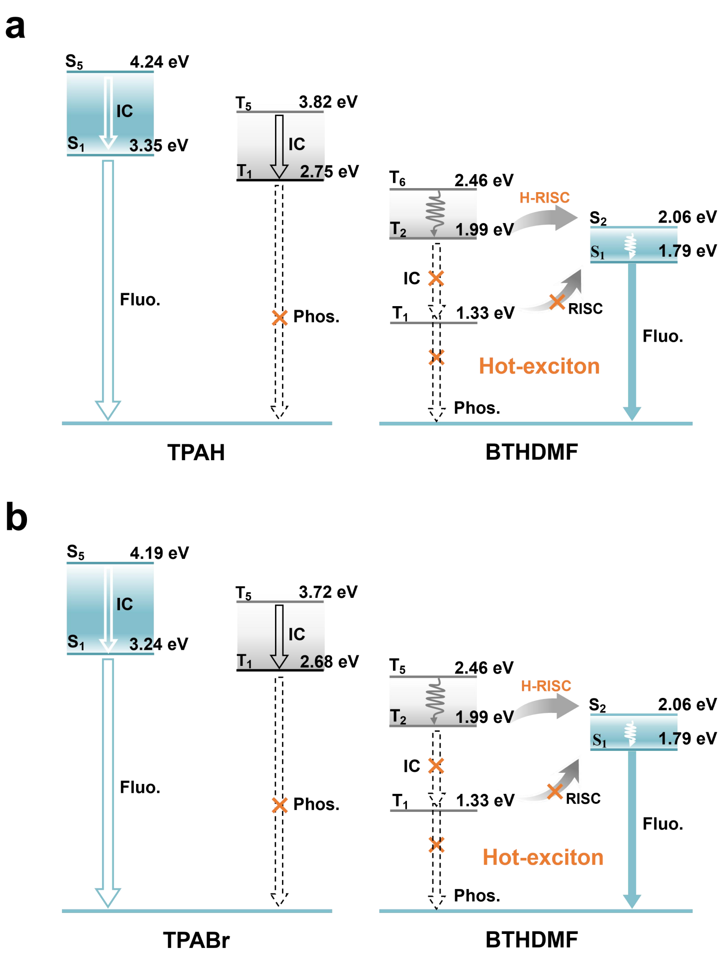

Figure S55. (a–b) Proposed photophysical pathways and energy transfer mechanisms in the G6 (TPAH–BTHDMF) and G7 (TPABr–BTHDMF) co-melting systems, respectively, including Dexter electron exchange, reverse intersystem crossing (RISC), and hybrid RISC (H-RISC) processes.

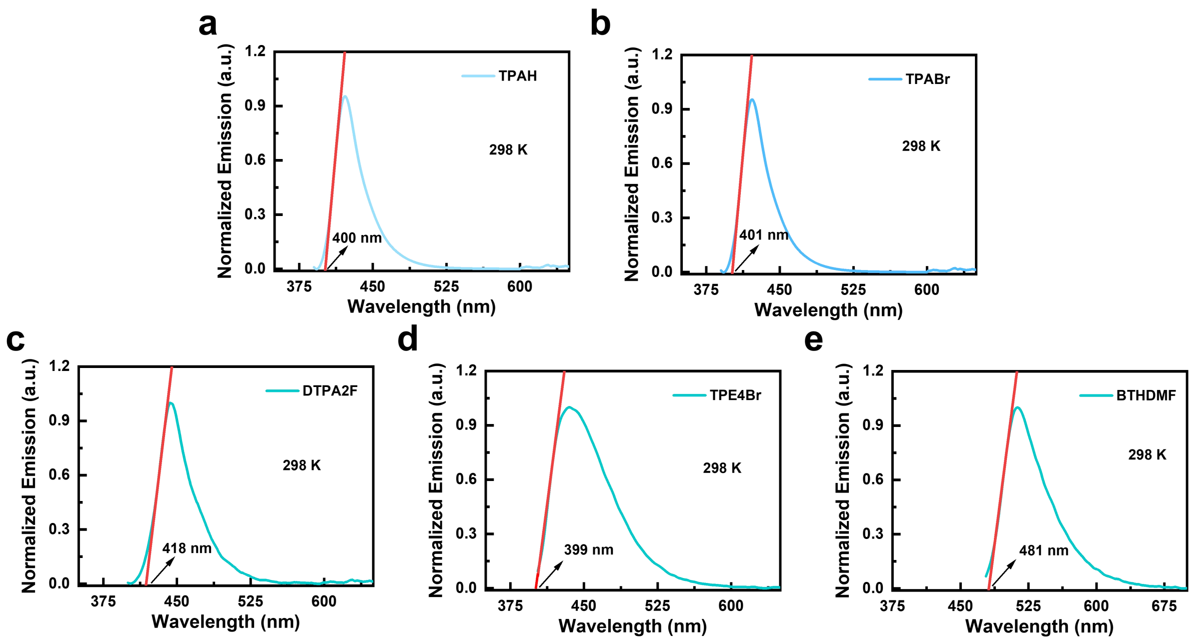

Figure S56. (a–e) The photoluminescence spectra at 298 K of TPAH, TPABr, DTPA2F, TPE4Br, and BTHDMF.

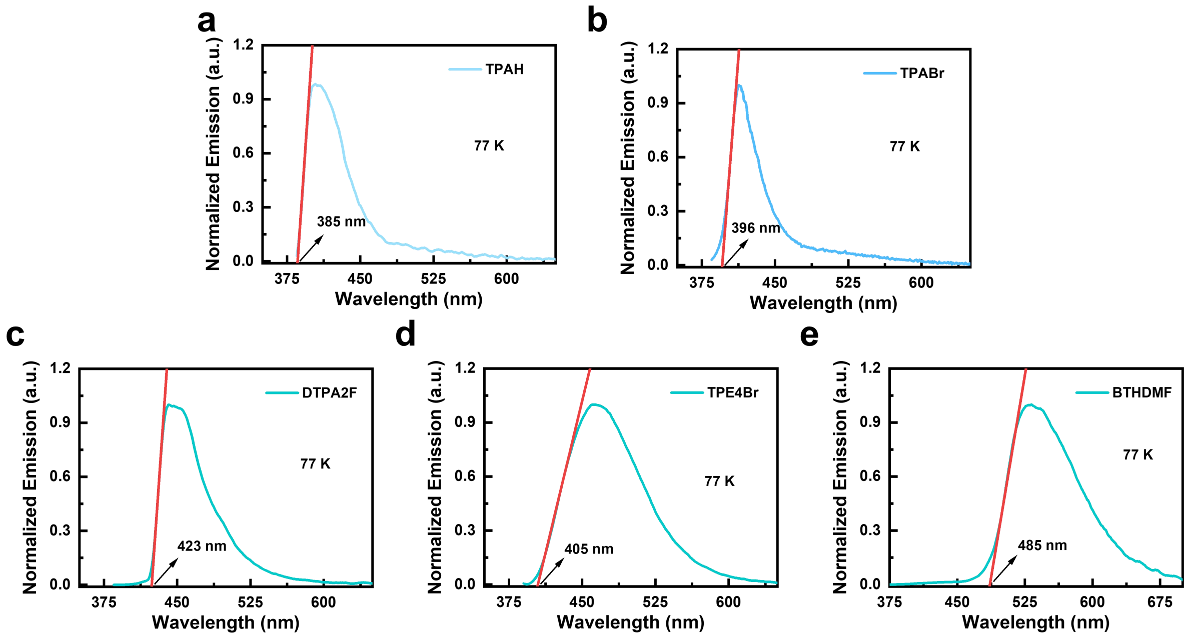

Figure S57. (a–e) The low-temperature photoluminescence spectra at 77 K of TPAH, TPABr, DTPA2F, TPE4Br, and BTHDMF.

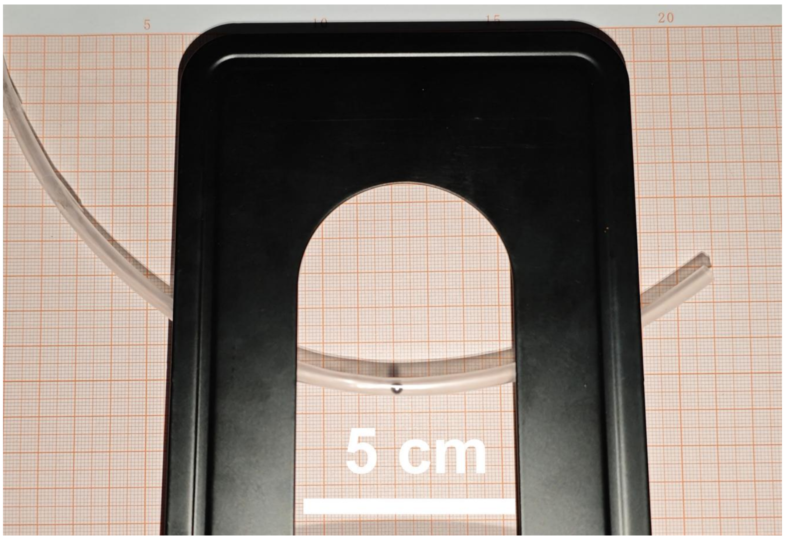

Figure S58. Photograph of a planar physical model simulating human blood flow using steel beads and tubing.

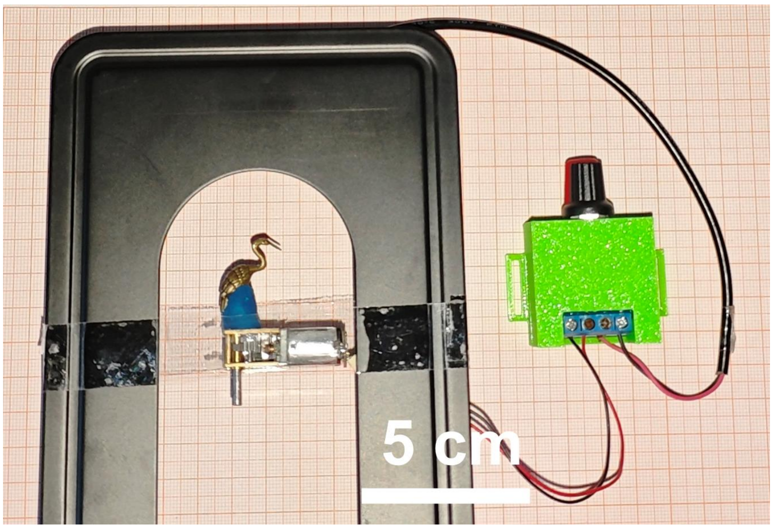

Figure S59. Photograph of the flat-panel experimental setup for acquiring X-ray images of a bird-shaped model. A mechanical motorized actuator is integrated to control the lateral positioning of the object.

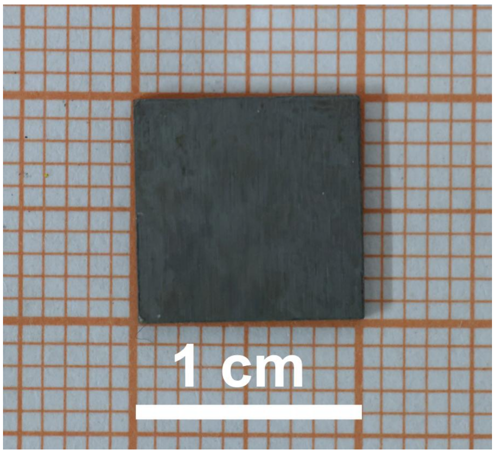

Figure S60. Optical photographs of tungsten foil.

Table S1. The crystallographic data of compounds TPAH, TPABr, DTPA2F, TPE4Br and BTHDMF.

	**TPAH**	**TPABr**	**DTPA2F**	**TPE-4Br**	**BTHDMF**
**CCDC number**	2428812	2428818	2357920	2435305	2435306
**Empirical formula**	$C_{24}H_{17}F_2N$	$C_{24}H_{16}BrF_2N$	$C_{42}H_{30}N_2F_2$	$C_{26}H_{16}Br_4$	$C_{36}H_{30}N_2S$
**Formula weight**	357.38	436.28	600.68	648.02	522.68
**Temperature/K**	293	293	296	296	293
**Crystal system**	monoclinic	Triclinic	Triclinic	monoclinic	triclinic
**Space group**	$P2_1/n$	P-1	P-1	$P2_1/n$	P1
***a*[Å]**	13.963(2)	7.6620(5)	10.0731(4)	20.506(3)	8.9366(5)
***b*[Å]**	8.3017(12)	9.9569(9)	12.6282(6)	9.890(13)	11.8199(6)
***c*[Å]**	16.541(3)	13.6989(13)	12.9970(6)	24.230(4)	13.6415(6)
**α [°]**	90	107.853(8)	100.540(4)	90	107.883(4)
**β [°]**	108.509(17)	92.544(6)	105.121(4)	91.100(4)	93.526(4)
**γ [°]**	90	100.752(6)	91.458(4)	90	102.120(4)
**Volume [Å^3]**	1818.2(5)	971.51(15)	1564.45(13)	4912.9(12)	1328.42(12)
**ρ_{calc} [gcm^{-3}]**	1.306	1.491	1.275	1.770	1.307
**μ[mm^{-1}]**	0.090	2.143	0.082	6.578	0.151
***F*(000)**	744	440	628	2521	552

$^a R_1 = \sum||F_0|-|F_c||/\sum|F_0|$. $^b wR_2 = \{\sum[w(F_0^2 - F_c)^2]/\sum[w(F_0^2)^2]\}^{1/2}$

Table S2. The X-ray dose rate versus voltage and current.

Glassy materials	Mass Ratio				
	**TPAH**	**TPABr**	**DTPA2F**	**TPE4Br**	**BTHDMF**
**G1**	0	0	100%	0	0
**G2**	50%	0	50%	0	0
**G3**	0	50%	50%	0	0
**G4**	50%	0	0	50%	0
**G5**	0	50%	0	50%	0
**G6**	67%	0	0	0	33%
**G7**	0	67%	0	0	33%

Table S3. The Young's moduli (*E*) of G1-G7.

Materials	E (GPa)			
	R1	R2	R3	Mean ± SD
**G1**	6.040	6.094	5.848	5.994 ± 0.129
**G2**	5.976	5.916	5.689	5.860 ± 0.151
**G3**	9.146	8.646	9.416	9.070 ± 0.391
**G4**	5.831	5.767	5.676	5.758 ± 0.078
**G5**	6.435	7.071	6.898	6.802 ± 0.329
**G6**	5.416	5.430	5.542	5.463 ± 0.069
**G7**	5.918	6.032	6.115	6.021 ± 0.099

Table S4. The Hardness (*H*) of G1-G7.

**Materials**	**H (GPa)**			
	**R1**	**R2**	**R3**	**Mean ± SD**
**G1**	0.137	0.133	0.132	0.134 ± 0.003
**G2**	0.251	0.277	0.238	0.255 ± 0.020
**G3**	0.280	0.279	0.312	0.290 ± 0.019
**G4**	0.213	0.213	0.200	0.209 ± 0.008
**G5**	0.204	0.237	0.213	0.218 ± 0.017
**G6**	0.192	0.194	0.194	0.193 ± 0.001
**G7**	0.212	0.217	0.212	0.214 ± 0.003

Table S5. The Young's moduli (E) and hardness (H) between organic co-melted glass and other reported or normal materials.

Materials	Composition	E (GPa)	H (GPa)	Ref.
**Organic co-melted glass**	G1	5.994 ± 0.129	0.134 ± 0.003	This work
	G2	5.860 ± 0.151	0.255 ± 0.020	
	G3	9.070 ± 0.391	0.290 ± 0.019	
**Polymer glass**	PS	1.9	0.1562	[12-15]
	ABS	2.7	0.1352	
	PET	3.6	0.2512	
**Organic–inorganic hybrid glass**	$(TPG)_2MnBr_4$	5.72	0.269	[12-15]
	$\alpha_G(PTP_{99}TPG_1)$	5.79	0.229	
	$\alpha_{G\text{-}C}(PTP_{99}TPG_1)$	5.52	0.260	
**Inorganic glass**	Sodium borosilicate	47.2	4.4	[12-15]
	Soda	70	4	
	Fused	72.5	9	

Table S6. The X-ray dose rate versus voltage and current.

Current (μA)	Voltage (kV)	Dose rate (μGy s^{-1})
5	20	4.58
5	30	9.64
5	40	11.91
5	50	17.38
10	50	34.75
20	50	69.50
30	50	104.25
40	50	139.00
50	50	173.75
60	50	208.50
70	50	243.25
79.9	50	278

Table S7. Distribution of singlet energy levels and corresponding spin-orbit coupling constants of TPAH, TPABr, DTPA2F, TPE4Br and BTHDMF molecules.

Molecular	Singlet energy levels (eV)				
	S_1	S_2	S_3	S_4	S_5
**TPAH**	3.35	3.72	3.91	4.15	4.24
**TPABr**	3.24	3.72	3.92	4.00	4.19
**DTPA2F**	2.96	3.20	3.67	3.67	3.83
**TPE4Br**	3.48	4.12	4.15	4.16	4.34
**BTHDMF**	1.79	2.06	2.43	2.45	2.75

Table S8. Distribution of triplet energy levels and corresponding spin-orbit coupling constants of TPAH, TPABr, DTPA2F, TPE4Br and BTHDMF molecules.

Molecular	Triplet energy levels (eV)				
	T_1	T_2	T_3	T_4	T_5
**TPAH**	2.75	3.19	3.40	3.61	3.82
**TPABr**	2.68	3.21	3.40	3.53	3.72
**DTPA2F**	2.50	2.79	3.18	3.18	3.37
**TPE4Br**	2.53	3.55	3.58	3.75	3.88
**BTHDMF**	1.33	1.99	2.26	2.35	2.46

Table S9. Reported performance of organic scintillators for X-ray imaging in the literature.

Composition	Imaging State	Resolution (lp mm^{-1})	Ref.
**G3**	Glassy state	30	This work
**CBP**	PDMS	14.3	[16]
**C4-I**	50 wt% CL	> 14.3	[17]
**DMAc-TRZ**	0.5 wt% in SO	16.6	[18]
**BIC**	5 wt% in PDMS	16.7	[19]
**9.10-DPA**	Single crystal	20	[20]
**DCB@C[3]A**	Microcrystalline	20	[21]
**Py2TTZ-I_2F_4**	Microcrystalline	26.8	[22]
**PAM-M4**	Glassy state	27	[23]
**D-$A_{0.5}$**	5 wt% in PSF	27.5	[24]
**PAM-4Br**	Glassy state	27.6	[25]
**HMB@mCPG**	Glassy state	30	[26]
**DTPA2F**	Glassy state	38.5	[27]

References

1 A.L. Spek, *J. Appl. Crystallogr*, 2003, **36**, 7-13.
2 L.J. Farrugia, *J. Appl. Crystallogr.*, 2012, **45**, 849-854.
3 G.M. Sheldrick, *Acta Crystallogr. C*, 2015, **71**, 3-8.
4 G.M. Sheldrick, *Acta Crystallogr. A*, 2015, **71**, 3-8.
5 A.D. Boese and J.M.L. Martin, *J. Chem. Phys.*, 2004, **121**, 3405-3416.
6 M. J. Frisch, G. W. Trucks, H. B. Schlegel, G. E. Scuseria, M. A. Robb, J. R. Cheeseman, G. Scalmani, V. Barone, G. A. Petersson, H. Nakatsuji, X. Li, M. Caricato, A. V. Marenich, J. Bloino, B. G. Janesko, R. Gomperts, B. Mennucci, H. P. Hratchian, J. V. Ortiz, A. F. Izmaylov, J. L. Sonnenberg, D. Williams-Young, F. Ding, F. Lipparini, F. Egidi, J. Goings, B. Peng, A. Petrone, T. Henderson, D. Ranasinghe, V. G. Zakrzewski, J. Gao, N. Rega, G. Zheng, W. Liang, M. Hada, M. Ehara, K. Toyota, R. Fukuda, J. Hasegawa, M. Ishida, T. Nakajima, Y. Honda, O. Kitao, H. Nakai, T. Vreven, K. Throssell, J. A. Montgomery, Jr., J. E. Peralta, F. Ogliaro, M. J. Bearpark, J. J. Heyd, E. N. Brothers, K. N. Kudin, V. N. Staroverov, T. A. Keith, R. Kobayashi, J. Normand, K. Raghavachari, A. P. Rendell, J. C. Burant, S. S. Iyengar, J. Tomasi, M. Cossi, J. M. Millam, M. Klene, C. Adamo, R. Cammi, J. W. Ochterski, R. L. Martin, K. Morokuma, O. Farkas, J. B. Foresman and D. J. Fox, Gaussian 16, Revision A.03, Gaussian, Inc., Wallingford CT, 2016.
7 W. Humphrey, A. Dalke and K. Schulten, *J. Mol. Graph.*, 1996, **14**, 33-38.
8 T. Lu and F. Chen, *J. Comput. Chem.*, 2012, **33**, 580-592.
9 H. Chen, M. Lin, Y. Zhu, D. Zhang, J. Chen, Q. Wei, S. Yuan, Y. Liao, F. Chen, Y. Chen, M. Lin and X. Fang, *Small*, 2024, **20**, 2307277.
10 H. Zhang, C. Li and Y. Duan, *Optik*, 2018, **157**, 635-643.
11 T. Huang, K. Li, J. Lei, Q. Niu, H. Peng and B. Zou, *Nano Res.*, 2023, **16**, 12680-12688.
12 Z. He, J. Luo, J. Chen, J. Wei, X. Miao, Z. Zhang, Q. Peng, X. Guo and D. Kuang, *Sci. Adv.*, 2025, **11**, eadu1982.
13 S. Li, R. Limbach, L. Longley, A.A. Shirzadi, J.C. Walmsley, D.N. Johnstone, P.A. Midgley, L. Wondraczek and T.D. Bennett, *J. Am. Chem. Soc.*, 2019, **141**, 1027-1034.
14 A.M. Hodge and T.G. Nieh, *Intermetallics*, 2004, **12**, 741-748.
15 W.O. Winer, A.E. Bergles, G.A. Klutke, K.K. Wang, I. Finnie, J.R. Welty, M.D. Bryant, H.T. Yang, Van C. Mow, F.A. Leckie and D. Gross, *Mech. Eng. Ser.*, edited by Vish Prasad (New York: Springer, 2011), ISSN 0941-5122, Electronic ISSN 2192-063X. https://link.springer.com/series/1161
16 H. Chen, M. Lin, C. Zhao, D. Zhang, Y. Zhang, F. Chen, Y. Chen, X. Fang, Q. Liao, H. Meng and M. Lin, *Adv. Opt. Mater.*, 2023, **11**, 2300365.
17 J.H. Wei, J.B. Luo, Z.L. He, Q.P. Peng, J.H. Chen, Z.Z. Zhang, X.X. Guo and D.B. Kuang, *Angew. Chem. Int. Ed.*, 2024, **63**, e202410514.
18 W. Ma, Y. Su, Q. Zhang, C. Deng, L. Pasquali, W. Zhu, Y. Tian, P. Ran, Z. Chen, G. Yang, G. Liang, T. Liu, H. Zhu, P. Huang, H. Zhong, K. Wang, S. Peng, J. Xia, H. Liu, X. Liu and Y.M. Yang, *Nat. Mater.*, 2022, **21**, 210-216.
19 Q. Sun, H. Wang, J. Li, F. Li, W. Zhu, X. Zhang, Q. Chen, H. Yang and W. Hu, *Small Struct.*, 2023, **4**, 2200275.
20 M. Chen, L. Sun, X. Ou, H. Yang, X. Liu, H. Dong, W. Hu and X. Duan, *Adv. Mater.*, 2021, **33**, 2104749.
21 G. Zhang, F. Chen, Y. Di, S. Yuan, Y. Zhang, X. Quan, Y. Chen, H. Chen and M. Lin, *Adv. Funct. Mater.*, 2024, **34**, 2404123.
22 Y. Chen, G. Zhang, F. Chen, S. Zhang, X. Fang, H. Chen and M. Lin, *Chem. Sci.*, 2024, **15**, 7659-7666.
23 X. Quan, G. Zhang, Y. Zhang, Q. Liao, H. Chen and M. Lin, *Adv. Opt. Mater.*, 2024, **12**, 2400113.
24 J.X. Wang, J. Yin, L. Gutiérrez Arzaluz, S. Thomas, W. Shao, H.N. Alshareef, M. Eddaoudi, O.M. Bakr and O.F. Mohammed, *Adv. Sci.*, 2023, **10**, 2300406.
25 Y. Guo, Z. Li, Q. Xin, D. Yiming, L. Yaofa, C. Hongming, Qing, Liao and Meijin Lin, *Sci. China Chem.*, 2025. https://doi.org/10.1007/s11426-025-2951-7
26 X. Fang, J. Zheng, J. Chen, X. Xu, Y. Ye, H. Chen and M.J. Lin, *Adv. Funct. Mater.*, 2025, e18212.
27 X. Yang, J. Chen, Y. Zhang, Y. Di, G. Zhang, S. Chen, H. Chen and M. Lin, *Chem. Sci.*, 2024, **15**, 18933-18942.
